# Health benefits and harms of older adult volunteering: mixed methods systematic review

**DOI:** 10.1093/geront/gnag043

**Published:** 2026-04-06

**Authors:** Nathan Williams, Marcelo Maghidman, Dai Pu, Debra Mitchell, Terry Haines

**Affiliations:** School of Primary and Allied Healthcare, Monash University, Frankston, Victoria, Australia; Department of Social Work, Monash University, Caulfield, Victoria, Australia; School of Primary and Allied Healthcare, Monash University, Frankston, Victoria, Australia; School of Primary and Allied Healthcare, Monash University, Frankston, Victoria, Australia; School of Primary and Allied Healthcare, Monash University, Frankston, Victoria, Australia

**Keywords:** Older adult, Aging, Volunteers, Health status, Quality of life

## Abstract

**Background and Objectives:**

Volunteering is recommended to promote healthy aging in older adults. This review investigated the benefits and harms of older adult volunteering on a range of health outcomes.

**Research Design and Methods:**

A systematic search of four databases (MEDLINE, CINAHL, AgeLine, PsycINFO) was conducted. Data were extracted and categorized according to health outcome. This review was registered on PROSPERO https://www.crd.york.ac.uk/PROSPERO/view/CRD42023456281RO.

**Results:**

The search yielded 182 papers related to a wide range of health benefits and harms. RCTs were assessed for risk of bias using CASP checklists. There were 11 RCT papers from five trials. Included RCTs had a high risk of bias. Benefits and harms were categorized into biological, psychological, social, and biopsychosocial intersect domains. Papers largely reported on multiple health benefits of volunteering. There were negligible reports of harms compared to benefits.

**Discussion and Implications:**

Volunteering is seemingly beneficial to older adults, though more evidence from experimental studies is needed to clarify discrepancies and substantiate current recommendations. Harms may be under-reported. Further clarity may allow for more older adults to participate in volunteering, while maximizing health benefits and avoiding harms. Additionally, further research could inform governments and volunteer organizations on how to implement and advertise programs appropriately.

## Background

The global older adult population continues to increase in number (World Health Organization, 2022). Healthy aging is a multifactorial concept that encompasses the physical, mental, and social health of older adults (World Health Organization, 2023). Governments are implementing public health strategies to facilitate older adults to age well (World Health Organization, 2023). Volunteering has been recommended as one way to encourage healthy aging (World Health Organization, 2024). Before the COVID-19 pandemic, nearly 15% of the world’s population were volunteering; many of these were retired and aged 65 years and over (International Labour Organization and United Nations Volunteers, 2025). The proportion of older adults who volunteer has been reported to have reduced during and after the COVID-19 pandemic (Dederichs, 2023; Grotz et al., 2020). This is concerning as volunteering may potentially improve a range of health outcomes for older adults (Anderson et al., 2014; Filges et al., 2020; Milbourn et al., 2018; Onyx & Warburton, 2003).

Facilitating healthy aging suggests that healthcare providers and stakeholders should consider biological factors, psychological factors, and social factors of health (Engel, 1977). Similarly, Rowe and Kahn’s concept of successful aging includes minimizing risk of disease and disability, maintaining physical and cognitive function, continuing engagement in life, and the intersect between these three domains (Rowe & Kahn, 1997). Regarding the Biopsychosocial model, biological factors include age and physical health; psychological factors include mental health and emotions; social factors include relationships and social supports. This Biopsychosocial Model of Health can be applied to categorize health benefits and harms to older adults who participate in volunteer work. Benefits may be a positive outcome in any of these areas of health, postvolunteering intervention (e.g., increased physical activity levels). Conversely, harms may be a negative outcome in any of these areas of health, postvolunteering intervention (e.g., increased stress and anxiety).

Previous work into the benefits of volunteering suggests a relationship between volunteering and improved health outcomes and there is reportedly substantial research available (Anderson et al., 2014; Filges et al., 2020; Milbourn et al., 2018). However, the quality of the evidence is reportedly poor (Nichol et al., 2023). Multiple reviews recommend that future research should include more randomized controlled trials (RCTs), rather than data from large data sets (Guiney & Machado, 2018; Von Bonsdorff & Rantanen, 2011). Multiple reviews only include one randomized controlled trial (Anderson et al., 2014; Milbourn et al., 2018). Reviews in this field tend to focus on benefits of those whom volunteers assist, or the effectiveness of a volunteer program, rather than benefits to the volunteer (Moore et al., 2021). One systematic review identified a positive association between volunteering and cognitive benefits (Sharifi et al., 2024). However, this review only identified one RCT, which focuses on physical cortical changes, rather than functional cognitive benefits. It limits its focus to cognitive outcomes, without exploring other possible benefits (Sharifi et al., 2024). Another systematic review suggested that improvements in mortality risk and depression favor volunteering (Filges et al., 2020), but there are other health outcomes that warrant investigating. Another systematic review on benefits (Chen et al., 2022) only focused on one type of volunteering: environmental volunteering. Therefore, the strength of the evidence to inform recommendations for older adults to volunteer could be improved. Furthermore, research has predominantly focused on the benefits associated with volunteering. This is not holistic, as there might be harms that are not being reported. No previous review has synthesized the adverse outcomes of volunteering, therefore, there is also a need to investigate the potential harms of older adult volunteering.

This review focused on formal volunteering programs. Formal volunteering programs require a level of organization by external parties to facilitate participation. A reported 30% of global volunteer activity occurs through organizations (United Nations Volunteers, 2018). This review sought to assist these organizations to better understand the benefits and potential risks that older adult participants may face when engaging in their programs. Formal volunteering programs have been defined across four domains; freedom of choice (e.g., being uncoerced or under no obligation), presence of remuneration (e.g., payment of a salary to participate), program structure [e.g., organized assistance in a classroom (formal), or helping a neighbor cross the street (informal)], and who the intended beneficiaries are (e.g., strangers, relatives, or oneself as well) (Cnaan et al., 1996). Programs can potentially be scaled across communities and as such can be conceptualized in ways similar to other public health programs. However, the decision to do so should be considered in light of the best available evidence of the benefits and potential harms of these programs.

This research aims to identify and synthesize the best available evidence regarding the benefits and harms to older adults who participate in formal volunteering.

## Methods

This systematic review was conducted following the Preferred Reporting Items for Systematic Reviews and Meta-Analysis (PRISMA) recommendations and was registered on PROSPERO https://www.crd.york.ac.uk/PROSPERO/view/CRD42023456281RO. A protocol was not prepared. The PROSPERO registration described investigation of two questions:

1. What are the health benefits and harms of older adult volunteering?
2. What are the enablers and barriers to older adult participation in volunteering?

This manuscript describes the results addressing the first question.

Databases Ovid MEDLINE, CINAHL, PsycINFO and AgeLine were systematically searched on June 16, 2023 with no date limits. The search strategy used for Ovid MEDLINE is included (Supplementary Material 1). An updated search with the same conditions was conducted on September 18, 2025.

### Inclusion criteria

Studies were selected if they included:

- Adults 50 and above, with a mean age (or majority percentage if mean not provided) of over 60
- Volunteering programs where volunteers freely choose to participate, no remuneration (except study reimbursement), a formal structure, not volunteering for short-term reasons, and intended beneficiaries being others and oneself also (Cnaan et al., 1996)
- Description of health benefits or harms for volunteers

### Outcomes

We included all health outcomes reported in included studies and grouped them into categories consistent with the biopsychosocial model of health (Engel, 1977). This model considers the biological, psychological, and social factors of health. There are some health outcomes that overlap across these three categories, such as overall health or quality of life. Outcomes were categorized into bio-, psycho-, social-, and intersect outcomes. Outcomes were combined where they were logically assessing the same health domain, despite different wording (e.g., self-purpose and purpose in life). Outcomes were combined where they could logically be categorized, with further detail subsequently provided for each particular outcome (e.g., the broader category of Self-Fulfillment contained outcomes relating to Self-Purpose and Self-Esteem).

### Data extraction

Included results were imported into the Covidence platform (Veritas Health Innovation, 2024) for screening. Title and abstract screening were completed by the lead author. A second reviewer screened papers concurrently with the lead author. Both team members continued independently screening and revealed results to each other sequentially until there was a consensus decision on 50 consecutive papers. Full text screening was completed by the lead author with assistance from team members. Key quantitative data extracted were study population, sample size, nature of volunteering intervention, outcomes, and measures used. Where relevant data were missing, the corresponding author was contacted via email to request the additional data.

### Risk of bias assessment approach

All included papers were assessed for risk of bias according to study design using CASP checklists (Critical Appraisal Skills Programme, 2023).

### Data analysis

For quantitative papers, numerical findings were interpreted based on whether they reported beneficial, harmful, or uncertain evidence for volunteering interventions. Significant results in favor of the volunteering intervention were presented as green ticks, significant results in favor of control were presented as red crosses, and results with nonsignificance were presented as orange question marks (see Supplementary Materials 5 to 7). Qualitative data were analyzed using content analysis (Forman & Damschroder, 2007) where participant responses and author observations were categorized into benefits or harms and further categorized into biological, psychological, social, or biopsychosocial intersect outcomes. Qualitative results in favor of volunteering were presented as green ticks, qualitative results where individual cases reported harms were presented as red Cs (see Supplementary Materials 5 to 7). Outcomes in each section (biological, psychological, social, or biopsychosocial intersect) were organized and presented by the highest number of RCTs for that outcome. Where no RCTs were included, outcomes were ordered by the highest number of highest quality studies according to evidence hierarchies (Commonwealth of Australia, 2009; Melnyk & Fineout-Overholt, 2022).

## Results

**Figure 1:**
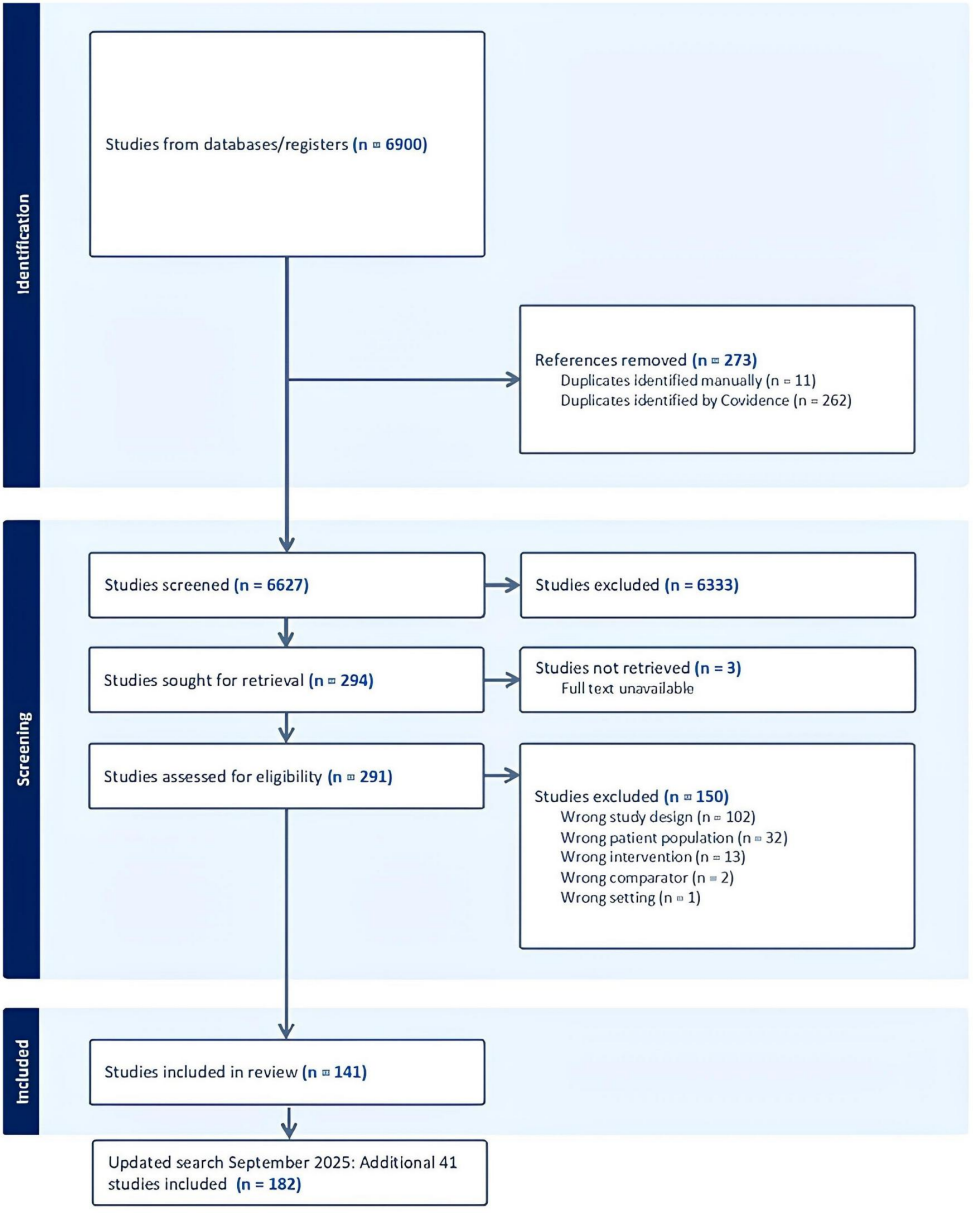
PRISMA diagram.

Flow of papers through our search strategy is presented (Figure 1). The initial search yielded 141 papers; the updated search yielded an additional 41 papers. Of the 182 total papers included in the final analysis, five were RCTs. One of these had outcomes described across five papers, and another two across two papers each, creating a total of 11 papers where outcomes from five RCTs were described. In addition, there were 86 cohort, 42 cross-sectional, 22 interview, ten mixed methods, five quasi-experimental, three focus group, and three case control papers. There were 91 papers from North America (including 88 from the United States); 33 from East Asia; 21 from Europe; 17 from Oceania; five from the Unite Kingdom; four from Southeast Asia; four from South America; three from West Asia; and fouracross different continents. Outcomes reported across these papers were categorized into bio-, psycho-, social-, and intersect biopsychosocial domains. Tables outlining detailed results from all 182 papers and a visual representation of results are included (Supplementary Materials).

### Outcome from risk of bias assessment

Detailed risk of bias assessment tables is included in Supplementary Material 3. All RCTs were assessed as being at high risk of bias. Other studies varied in their bias risk.

### Biopsychosocial health outcomes

There were 58 health outcomes that were categorized into Biological (28 outcomes), Psychological (17 outcomes), Social (8 outcomes), and Intersect Biopsychosocial (five outcomes) domains. A summary of each of these categorized outcomes is included below. There were a variety of studies with diverse samples and diverse volunteering interventions. For further detail, characteristics of each study are included in Supplementary Material 2 and a summary of results from each paper for each outcome is included in Supplementary Materials 4 to 7.

#### Biological outcomes

Biological health typically addresses physical health and often involves consideration of health conditions, physiological responses, and bodily health (Engel, 1977). Outcomes categorized as biological included physical health, activity, and fitness; strength; independence with activities of daily living (ADLs) and instrumental activities of daily living (IADLs); body weight/obesity; walking endurance and speed; stair climbing speed; chronic health condition incidence; risk of falls, hip fracture, objective measures of frailty and dysphagia; dynamic balance; mortality rate; cardiovascular health; blood sugar levels; Conserved Transcriptional Response to Adversity (CRTA) gene expression; epigenetic age; C-reactive protein (CRP) levels; healthier eating habits, improved sleep quality; reduced binge drinking; reduced smoking; and increased brain volume. A summary table of results for each outcome is included in Supplementary Material 4.

### Physical activity

Physical activity was mostly measured by amount of walking via accelerometry or with a global physical activity scale (see Supplementary Material 4). There were 24 papers that reported on physical activity. There were two RCT papers reporting significant improvements in physical activity for volunteers and two reported no significant differences between groups (Fried et al., 2004; Parisi et al., 2015; Pettigrew et al., 2020; Tan et al., 2006). However, three of these papers reported data from the same parent trial. There were 12 additional papers reporting significant associations between volunteering and increased physical activity; two of these both reported this significant association was only apparent in those who volunteered more than 100 h per year (Jirovec & Hyduk, 1999; Kim et al., 2020). A further five papers reported no significant associations (see Supplementary Material 4). There were three qualitative papers where volunteers reported improvements in their physical activity levels, “This (volunteering) keeps me active…and my physical body moving” (Landry, 2017).

### Walking speed

Walking speed was commonly measured by the time taken to walk 3 to 6 m at a usual pace. There were four papers that reported on walking speed. One RCT reported significant improvements in the volunteering group and another RCT reported no significant differences between groups. A further two cohort papers reported no significant associations between walking speed and volunteering (see Supplementary Material 4).

### Strength

Strength measures included hand grip strength, time of sit to stand transfers and chest press one-repetition maximum lift. There were five papers that reported on strength. One RCT reported significant improvements in the strength of volunteers and one RCT reported no significant improvements. An additional paper reported a significant improvement in strength of volunteers and two papers reported no significant improvements (see Supplementary Material 4).

### Physical health

Physical health was commonly measured with a single survey item with Likert scale responses. There were 13 papers that reported on physical health. There were four papers that reported significant improvements in the physical health of volunteers and five papers reported no significant improvements (see Supplementary Material 4). There were three qualitative papers where participants reported improvements in their physical health, “Personally, my health has improved so much because of…volunteering and being active. I don’t take any prescriptions” (Misener et al., 2010). In contrast, one RCT reported an improvement in physical health in favor of the control group, which could be interpreted as volunteering being harmful for participants’ physical health. However, both groups did make improvements in their physical health compared to baseline across the 12-week study (Yuen et al., 2008).

### Body weight/obesity

This outcome was commonly measured by body mass index. This included reduced central adiposity. There was one RCT (Pettigrew et al., 2020), one quasi-experimental (Hsiao et al., 2020) and two other papers that reported no significant associations between volunteering and decreased body weight (see Supplementary Material 4).

### Chronic health condition incidence

Number of chronic health conditions were commonly self-reported through questionnaires. There were five papers that reported significant associations between reduced incidence of chronic health conditions in volunteers; two of these papers both reported this association was only apparent for those who volunteered for 50 to 99 h per year (Jirovec & Hyduk, 1999; Kim et al., 2020). One RCT (Pettigrew et al., 2020) and three other papers reported no significant associations.

### Walking endurance

This outcome was measured by an ability to walk longer distances and number of blocks reportedly walked per week. There were two papers that reported on walking endurance. One RCT reported no significant differences between volunteer and control groups on walking endurance and one cohort paper reported no significant associations (see Supplementary Material 4).

### Physical fitness

Physical fitness was measured via resting heart rate or questionnaire. There were two papers that reported on physical fitness. One paper reported a significant improvement in fitness for volunteers and one RCT reported no significant improvement (see Supplementary Material 4).

### Sleep quality

This outcome was measured by sleep duration and sleep-related questionnaires. There were five papers that reported on sleep quality; one RCT and four additional papers all reported no significant associations between sleep quality and volunteering.

### Dynamic balance

Dynamic balance was measured by time taken to walk 6 m backwards. One RCT reported no significant differences between volunteer and control groups.

### Brain volume

One RCT investigated cortical and hippocampal volume in volunteers using magnetic resonance imaging. The authors reported significant increases in volume for men and no significant changes in women (see Supplementary Material 4).

### Cardiovascular health

This outcome included incidence of cardiovascular events (e.g., heart attack), hypertension risk, lipid dysregulation, and cholesterol levels. There were 15 papers that reported on cardiovascular health. Two papers reported significant associations between volunteering and improved cardiovascular health and two papers reported no significant associations. One cohort study reported an increase in lipid dysregulation for volunteers (Burr et al., 2016). The authors reported this may have been due to the physical activity involved in volunteering and that it may not be an accurate measure of cardiovascular disease risk (Burr et al., 2016). Four papers reported a significant association between volunteering and reduced hypertension; one of these reported this was only apparent for those who volunteered more than 200 h per year (Sneed & Cohen, 2013). Another two papers reported no significant associations between volunteering and hypertension risk. An elevated CRP level is a marker of immunological function, that is, said to be highly predictive of cardiovascular events (Bell et al., 2022). There were three papers that reported significantly reduced CRP levels in volunteers and one paper reported no significant associations (see Supplementary Material 4).

### Independence with IADLs

IADLs are more complex tasks relating to maintaining independence in the community, for example, paying bills and shopping. Volunteering was consistently associated with increased independence with IADLs, in four quantitative papers (see Supplementary Material 4).

### Mortality rate

Mortality was measured via all-cause mortality over time, using national databases and through interviews with loved ones. Results were predominantly in favor of reduced mortality rate amongst volunteers, with 16 papers reporting significant associations between volunteering and reduced mortality rate and one paper that reported no significant associations (see Supplementary Material 4).

### Independence with ADLs

Completing ADLs includes personal care tasks and personal day-to-day activities. This included outcomes described as Physical Function. Independence with ADLs was often measured by the SF-12 scale and self-ratings on ability to do certain ADL tasks. Results were predominantly in favor of volunteering benefiting one’s independence with ADLs. There were 22 papers that reported on independence with ADLs. There were 15 papers that reported significant associations between volunteering and increased independence with ADLs and five reported no significant associations. There were two qualitative papers where participants reported being more independent with their ADLs, “Volunteering has actually been my OT/PT. I have learned how to write better…who do you think taught me all those things? The children!” (Sellon, 2018).

### Frailty risk

Frailty was measured objectively using frailty scales and checklists. There were three papers that reported on frailty risk. There was one paper that reported a significant association between volunteering and decreased frailty risk; two papers reported no significant associations.

### Stair climbing speed

This outcome was measured as the time taken to climb a flight of stairs. There was one paper that reported no significant associations between volunteering and stair climbing speed.

### Falls risk

For this outcome, participants were asked the number of times they had fallen in the past year. One cross-sectional study reported that volunteering was significantly associated with a higher risk of falls (Moncayo-Hernández et al., 2024). This increased risk could be considered a potential harm of older adult volunteering. The authors reported that this increased risk may have been due to the participants living in cities where they may have encountered more environmental barriers more often due to their active lifestyles, which may have led to increased falls.

### Hip fracture risk

Risk of hip fracture was measured via hip fracture incidence and whether or not a hip fracture had occurred over time. One case control paper reported a significant association between volunteering and reduced risk of hip fractures.

### Blood sugar levels

Two papers reported no significant associations between volunteering and decreased blood sugar levels, although there was no mention of the participants having Type II diabetes, where this might be an expected outcome of possible increased physical activity in volunteers.

### Epigenetic age

Epigenetic age measures the biological age of cells and human tissue, as opposed to measuring one’s chronological age in years (Kim et al., 2025). This was measured by taking a biological sample that was compared to 13 DNA clocks. One paper reported significantly reduced epigenetic age in volunteers and one paper reported no significant associations between volunteering and epigenetic age.

### CRTA gene expression

CRTA activation has been reported to be involved with inflammation and has been seen in those exposed to adverse life circumstances (Seeman et al., 2020). One paper reported a significant decrease in CRTA gene expression in volunteers.

### Dysphagia risk

Dysphagia is difficulty swallowing and was measured using the “Dysphagia Risk Assessment for Community Dwelling Elderly” instrument (Mayers et al., 2024). One paper reported no significant association between volunteering and dysphagia risk.

### Healthy eating habits

Healthy Eating was measured using the Dietary Variety Score (Abe et al., 2022); one paper reported a significant association between volunteers and healthy eating habits.

### Smoking

Smoking status was measured with a single item on a questionnaire. There was one paper that reported no significant association between decreased smoking and volunteering.

### Binge drinking

This outcome was measured by asking the number of days where more than four drinks were consumed in one week. There was one paper that reported no significant association between decreased binge drinking and volunteering.

#### Psychological outcomes

Psychological health addresses one’s own mental health, cognitive abilities, beliefs, emotions, and behavior (Engel, 1977). Outcomes categorized as psychological included depression symptoms, life satisfaction, psychological wellbeing, psychological resources, self-fulfillment, intellectual activity, cognitive function, cognitive impairment incidence, cognitive capital, stress/anxiety, personal growth/learning, fear of falling, resilience, hopelessness, self-efficacy with ADLs, health satisfaction, attitude toward aging, lifestyle, faith development, and willingness to live. A summary table of results for each outcome is included in Supplementary Material 5.

### Depression symptoms

Symptoms of depression can include feeling low and empty, hopeless, changes in sleep and appetite, among others (World Health Organization, 2025). Depression symptoms were commonly measured with the Center for Epidemiologic Studies Depression Scale (Hong & Morrow-Howell, 2010) or the Geriatric Depression Scale (Hsiao et al., 2020). There were 37 papers that reported on depression symptoms. There were five RCT papers that reported improvements in depression symptoms with volunteering, one of which was a significant improvement (Warner et al., 2024); the other four were insignificant improvements (George & Singer, 2011; Jongenelis et al., 2022a; Yeung et al., 2025; Yuen et al., 2008). One of these papers was from the same trial as Warner (2024) at a later time point (Yeung et al., 2025). Papers from other study designs were largely in favor of improved depression scores in volunteers. There were 20 additional papers that reported significant improvements or significant associations between improved depression symptoms and volunteering. There were several papers where the reported results were significant in only certain subgroups: those who were not recently widowed (Johnson, 2013); those volunteering over 100 h per year (Kim et al., 2020); those volunteering 101 to 300 h per year (Webster et al., 2021); those volunteering no more than 100 to 799 h per year (Windsor et al., 2008); and men but not women (Mechakra-Tahiri et al., 2010). Additionally, there were 12 papers that reported an insignificant association or changes in depression scores for volunteers (see Supplementary Material 5).

### Life satisfaction

This outcome was commonly measured using the Satisfaction with Life Scale or a single item with Likert scale responses. There were 35 papers that reported on life satisfaction. There were four RCT papers that reported increased life satisfaction scores with volunteering, one of which was a significant improvement (Jongenelis et al., 2022a); the other three were insignificant improvements (Pettigrew et al., 2020; Yeung et al., 2025; Yuen et al., 2008). One of these papers was from the same trial as Pettigrew et al. (2020) at a later time point (Jongenelis et al., 2022a). There were 15 papers that reported positive associations between increased life satisfaction and volunteering. One of these reported this was only significant in those who did not volunteer for social awareness causes (Gil-Lacruz et al., 2019). There were 12 papers that reported insignificant associations between life satisfaction and volunteering (see Supplementary Material 5). There were three qualitative studies where volunteers reported an increased satisfaction with life, “it has changed my life a lot for the better” (Labegalini et al., 2015). There was one cohort study paper that reported a significant decrease in life satisfaction scores for volunteers who participated in “higher levels of volunteering” (Bjälkebring et al., 2021), however, the amount of hours, that is, considered a “higher level” is not specified by the authors.

### Psychological wellbeing

Psychological wellbeing included mental health, increased happiness, pleasure, positive affect, and improved eudemonic wellbeing. These were measured with scales and with single items on questionnaires with Likert scale responses (see Supplementary Material 5). There were 48 papers that reported on psychological wellbeing. There was one RCT paper that reported a significant increase in psychological wellbeing scores for volunteers compared to control (Jongenelis et al., 2022a) and two RCT papers reported nonsignificant improvements (Pettigrew et al., 2020; Yeung et al., 2025). One of these papers was from the same trial as Pettigrew et al. (2020) at a later time point (Jongenelis et al., 2022a). There were 22 papers that reported significant associations between volunteering and improved psychological wellbeing. One of these reported significance was only apparent for those who volunteered no more than 100 to 799 h per year (Windsor et al., 2008). Six other papers reported no significant associations. One paper reported a significant decrease in psychological wellbeing scores in social awareness volunteering, for example, environmental causes, and no significant relationships for other volunteering types (Gil-Lacruz et al., 2019). Another paper reported decreased psychological wellbeing in volunteers who felt overwhelmed, with no significant relationship for other volunteers (Jongenelis et al., 2022b). There were 15 qualitative papers where majority of volunteers reported improved psychological wellbeing, with some discrepancies in individual experiences (see Supplementary Material 5). For example, one participant felt upset when receiving negative feedback, “Sometimes you get criticized for trying to help and, that is, the most frustrating part. You give your time and you get criticized” (Misener et al., 2010); one participant felt upset from volunteering alongside disadvantaged children, “Your heart goes out to them…sometimes they will make you want to cry” (Varma et al., 2015).

### Self-fulfillment

Self-fulfillment included self-esteem, self-purpose, self-worth, self-compassion, self-efficacy, optimism, feelings about oneself, feeling of usefulness, psychological resources, and will to live. There were 41 papers that reported on self-fulfillment. There was one RCT paper that reported a significant increase in self-fulfillment scores for volunteers compared to control (Jongenelis et al., 2022a) and two RCT papers reported nonsignificant improvements (George & Singer, 2011; Pettigrew et al., 2020). One of these papers was from the same trial as Pettigrew et al. (2020) at a later time point (Jongenelis et al., 2022a). There were 16 other papers that reported significant associations between volunteering and greater self-fulfillment. One of these reported this significant improvement was only apparent for those who did not feel overwhelmed, whereas those who felt overwhelmed had a significant decrease in self-fulfillment (Jongenelis et al., 2022b). Another of these 16 papers reported significantly improved self-fulfillment only in those who volunteered at least 100 h per year (Kim et al., 2020). A further seven papers reported no significant relationships. A further 15 qualitative papers had volunteers who consistently reported increased self-fulfillment, “It gives me a purpose;” “Volunteering saved my life. I literally would be dead if I didn’t have this to do. (It is the) reason that I get up in the morning” (Landry, 2017).

### Cognitive functioning

This outcome included improved cognitive health, cognitive capital, improved memory, and improved episodic memory. There were 23 papers that reported on cognitive functioning. Three RCT papers reported nonsignificant improvements in cognitive functioning of volunteers (Brydges et al., 2021; Fried et al., 2004; George & Singer, 2011). One of these used data from the Fried et al. (2004) trial (Brydges et al., 2021). Another 15 papers reported significant positive associations between volunteering and higher cognitive function. One of these papers reported significance was only apparent with those who volunteered no more than 50 to 99 h per year (Kim et al., 2020); another reported significantly improved cognitive function was only apparent for unmarried women, whereas significantly reduced cognitive function was apparent for volunteers who were married men (Monserud, 2025). Four additional papers reported no significant associations. One qualitative paper had volunteers reporting maintained cognitive function, “My brain will degenerate slowly (as opposed to quickly) because it is used whenever I do my volunteer job” (Chen, 2016).

### Stress/anxiety

This outcome was measured using scales such as the Beck Anxiety Index. There were ten papers that reported on stress/anxiety. Two RCT papers both reported significant desirable reductions in stress symptoms of volunteers compared to control (George & Singer, 2011; Warner et al., 2024). Another paper reported a significant association between volunteering and reduced stress and one reported no significant association. Four qualitative papers had volunteers who reported reduced stress, “So in my mind, it is just all good and (volunteering) overrides (the bad)” (Sellon, 2023) and “(volunteering is) good therapy. It takes my mind off my worries…” (Warburton and Winterton, 2017).

However, there were discrepancies with individual experiences, with some volunteers reporting increased stress and anxiety with a new task and in some cases (e.g., hospice care) a confronting environment, “I feel very anxious. I wonder whether I will be able to perform” (Matsuda et al., 2024) (see Supplementary Material 5). One RCT paper reported desirable significantly decreased stress scores in the volunteering group at 6-months, however, the reverse was true at 12-months follow-up, where the control group was reportedly significantly less stressed (Yeung et al., 2025).

### Intellectual activity

Intellectual activities include completing puzzles or playing an instrument. There were consistent reports of significantly increased intellectual activity abilities and volunteering in three studies, including one RCT (see Supplementary Material 5).

### Personal growth/learning

This outcome included any new skills, personal growth or learnings, including recognition of lifelong learning. It was commonly measured using Ryff’s Psychological Wellbeing Scale and single items with Likert scale responses. There were nine papers that reported on personal growth/learning. One RCT reported significantly more personal growth in volunteers compared with control and another reported insignificant differences between groups (Jongenelis et al., 2022a; Pettigrew et al., 2020). These papers used data from the same trial at two different time points. An additional paper reported significant associations between volunteering and more personal growth and another reported no significant associations. There were five qualitative papers where volunteers consistently reported more personal growth experiences, volunteers said, “I learned a lot” (Labegalini et al., 2015) and “That experience…taught me something too” (Fields et al., 2023).

### Cognitive impairment incidence

There were five papers that reported significant associations between volunteering and reduced diagnoses of dementia and cognitive impairment (see Supplementary Material 5). One of these reported the significant association was only apparent for those who volunteered no more than 50 to 99 h per year (Jirovec & Hyduk, 1999).

### Resilience

Resilience refers to being able to cope with challenges and changes in life (Resnick et al., 2013). One paper reported significantly greater resilience in volunteers and another reported no significant association (see Supplementary Material 5).

### Hopelessness

One paper reported significantly reduced hopelessness in those who volunteered for at least 100 h per year and no significant association for those who volunteered less (Kim et al., 2020).

### Attitude toward aging

This outcome was commonly measured using scales or captured through interviews. There were 10 papers that reported on attitude toward aging. There were three papers that reported a significant association between volunteering and an improved attitude toward aging; one of these reported this was only significant for those who volunteered at least 100 h per year (Huo et al., 2021). One other paper reported no significant association. There were six qualitative papers where participants consistently reported an improved attitude toward aging with volunteering, one participant said when volunteering “you look at life differently … you feel younger as well” (Jones & Reynolds, 2019).

### Faith development

Faith development involves progression of one’s faith, meaning-making, and spiritual beliefs and practices (Myers et al., 2013). One paper reported significantly deeper faith development in volunteers and another reported a nonsignificant association between volunteering and religiosity (see Supplementary Material 6).

### Will to live

Two papers reported consistently significant associations between volunteering and an increased will to live.

### More health satisfaction

One paper reported that volunteers felt significantly more satisfied with their health (see Supplementary Material 5).

### Fear of falling

One paper reported no significant association between volunteering and fear of falling (see Supplementary Material 5).

### Lifestyle

One paper reported a significant association between being a volunteer and having a desirable less sedentary lifestyle.

#### Social outcomes

Social health addresses one’s peer and family relationships, interpersonal interactions, connectedness and support, living situation, environment, and culture (Engel, 1977). Outcomes categorized as social included social connectedness, social support, satisfaction with interpersonal relationships, social role, loneliness, compassion for others, driving ability, and environmental quality of life (QOL).

### Social connectedness

Social connectedness was commonly measured with scales such as the Short Form Survey, or single items with Likert scale responses. There were 41 papers that reported on social connectedness. There was one RCT paper that reported a significant increase in social connectedness in volunteers compared to control and three RCT papers reported nonsignificant differences. One of these reported on the data from Pettigrew et al. (2020) at a later time point (Jongenelis et al., 2022a) and one of these reported on data from Warner et al. (2024) at a later time point (Yeung et al., 2025). There were 12 papers that reported significant differences or associations in increased social connectedness of volunteers. One of these reported this significant result was only apparent in gay men and not lesbian women (Lyons et al., 2021). There were six papers that reported nonsignificant improvements in the social connectedness of volunteers. There were 18 qualitative papers where participants predominantly reported increased social connectedness with volunteering, “It’s like…an extended family” (Fraser et al., 2009). However, one paper reported a discrepancy in an individual experience, where a volunteer said because of the demographic of fellow volunteers, “we are going to lose some friends [who pass away]” (Misener et al., 2010) (see Supplementary Material 6).

### Social support

Support was often measured based on questionnaire items asking about the nature of relationships. It also included frequency of social contact, size of social network, and rating of friendships. There were 11 papers that reported on social support. One RCT paper reported increased social support for volunteers compared to the control group (Fried et al., 2004) and two RCT papers reported nonsignificant differences between groups; the latter two were from the same trial at two different time points. There were seven papers that reported a significant association between volunteering and increased social support. One of these papers reported this significant result was only apparent in gay men and not lesbian women (Lyons et al., 2021). Another paper reported no significant differences in social support for volunteers (see Supplementary Material 6).

### Loneliness

Loneliness was commonly measured using the UCLA Loneliness Scale. There were 18 papers that reported on loneliness. One RCT paper reported significantly desired reduced loneliness for volunteers compared with control and another reported a nonsignificant reduction in the loneliness of volunteers. The latter paper was from the same trial as the initial paper at a later time point (Warner et al., 2024; Yeung et al., 2025). Additionally, eight papers reported associations between reduced loneliness and volunteering. Several of these reported this significant result was only apparent for certain yearly hours of volunteering: those who volunteered at least 100 h per year (Jirovec & Hyduk, 1999; Kim et al., 2020) and those who volunteered 1–99 h per year (Akhter-Khan et al., 2023). Another five papers reported nonsignificant improvements or association between loneliness and volunteering. One paper reported a significant increase in loneliness for those volunteering for political causes (Lühr et al., 2022) (see Supplementary Material 6). There were two qualitative papers where participants consistently reported reduced loneliness with volunteering, “If you are active and alone there are so many things you can do (to feel less lonely). I mean there are all the charity, volunteer things you can join” (White et al., 2025).

### Compassion for others

This outcome was measured with a Compassion Scale and captured in interview responses. There were consistent results in seven papers that reported an association between volunteering and being more compassionate toward others. There was one paper that reported a significant association between volunteering and having more compassion for others. There were six qualitative papers where participants reported the same, “I want to help other people. I thought (volunteering) would be a good place to help other people” (Sellon, 2023).

### Environmental QOL

Environmental QOL addressed physical home environment and safety and access to services and transport. It was assessed using the WHO QOL-Bref measure. One paper reported no significant association between volunteering and Environmental QOL (see Supplementary Material 6).

### Satisfaction with interpersonal relationships

One paper reported no significant differences between volunteers and nonvolunteers in their satisfaction with interpersonal relationships (see Supplementary Material 6).

### Social role

Social role refers to one’s position in society and may include employment, member of a family and friendship group; it is likely influenced by culture (Shimanuki et al., 2007). One paper reported a significant association between volunteering and having a more active social role (see Supplementary Material 6).

### Driving ability

One paper reported a significant association between volunteering and continued driving ability.

#### Intersect biopsychosocial outcomes

The intersect of biopsychosocial health considers that not all health considerations fit in only one category and there is overlap in this model (Engel, 1977). Outcomes categorized as intersect biopsychosocial included health, QOL, pain, energy levels and fear of harm (see Supplementary Material 7).

### Quality of life

Overall QOL was measured using different QOL scales (see Supplementary Material 7). There were eight papers that reported on overall QOL. One RCT paper reported nonsignificant improvements in the quality of life of volunteers (Pettigrew et al., 2020). Additionally, six papers reported significantly improved quality of life in volunteers and one paper reported no significant association (see Supplementary Material 7).

### Health

This outcome referred to general health when it was not referred to as physical or mental or otherwise. This was often self-rated with a single item with Likert scale responses. There were 26 papers that reported on health. There were 24 papers that reported significantly improved health or a significant association between improved health and volunteering. Several of these reported significant results were only apparent in subgroups: those who participated in volunteering other than religious or social awareness causes (Gil-Lacruz et al., 2019); those who were not recently widowed (Johnson, 2013) and gay men but not lesbian women (Lyons et al., 2021). One paper reported a significant decrease in the health of volunteers compared to control, however, this may have been due to variance in the geographical regions of the participants in each group (Shimanuki et al., 2007). One additional qualitative study had participants who reported increased health with volunteering, “I think my health is improving since I started volunteering” (Hwan & Hussin, 2022).

### Pain

Pain was measured with single items on questionnaire with a yes/no response or numerical rating scale. There were six papers that reported on pain. Four papers reported no significant associations between pain and volunteering. Two qualitative papers consistently reported decreased pain with volunteering, “I felt no pain at all. It was just amazing…it was almost like a drug” (Lee et al., 2021).

### Energy levels

Energy levels were assessed through numerical rating, a single item with Likert scale responses, or through responses to interview questions. There were five papers that reported on energy levels. One paper reported a significant increase in feeling more full of energy in volunteers (Weziak-Bialowolska et al., 2024). In contrast, one paper reported a significant less desirable increase in “feeling tied up” with volunteering (Celdrán & Villar, 2007). Two papers reported no significant associations between volunteering and energy levels. However, one qualitative paper had participants who reported increased energy levels through volunteering, “I’ve noticed that you feel more lively…[with] those four years-olds there’s no time for laid back” (Varma et al., 2015).

### Fear of harm

A participant in one qualitative study expressed a fear of harm while volunteering with school children who engage in physical fights, “I know I’m going to have to break up some fights…these are not little love taps, they’re serious” (Varma et al., 2015).

## Discussion

Volunteering largely appears to be beneficial to the biopsychosocial health of older adults, with a risk of some minor harms. Papers largely report beneficial improvements in a range of health outcomes, although there is a lack of high-quality experimental studies. There is a need for additional experimental studies to support or dispute the multiple benefits reported and to further support the current recommendations for older adults to volunteer.

The number of reported harms were greatly outweighed by the reported benefits. It is possible that harms are being under-reported by volunteers, stakeholders, and in the literature. Quantitative studies may have used measures where harms were difficult or unable to be detected. Researchers may have also been biased by largely focusing on the benefits of volunteering. Harms were more often reported in qualitative studies, where the methodology may have allowed for participants to more fully share their individual perceptions, including their negative experiences. These harms were mostly reported in individual participants and were mostly discordant with other nonexperimental studies, which largely reported benefits. However, it would appear that although the majority of studies largely report on benefits, this may not apply to all older adults, as individuals within these groups reported harms. These inconsistencies further highlight the need for additional high-quality experimental studies to provide clarity on the suggested benefits and recommendations for older adults to volunteer, while also considering the risk of harm to individuals. Furthermore, it appears that the type of volunteering, and the amount of time spent volunteering and an associated sense of feeling overwhelmed, may be factors in making volunteering potentially harmful to older adults’ health. There is a need to further investigate how the type of volunteering activity and the amount of yearly time spent volunteering may affect health outcomes.

A concern in reviewing the papers reporting results from RCTs in this field was that five papers drew their data from the Baltimore Experience Corps^®^ Trial (Fried et al., 2004, 2013). Although reference to the earlier study is explicit in each paper, it could be argued that these repeated papers are examples of salami publication (Šupak Smolčić, 2013). Other issues of concern in included RCTs were that the initial analysis approach did not always account for cluster randomization (increasing the risk of generating confidence intervals that are too narrow and Type I errors) (Fried et al., 2004), and seemingly inaccurate reporting of per-protocol analyses as being “by allocated condition” (Jongenelis et al., 2022a; Pettigrew et al., 2020). In addition, there were two occasions where papers reported data from the same trial at two different time points, Warner (2024) and Yeung et al. (2025); and Pettigrew et al. (2020) and Jongenelis et al. (2022b). Despite these criticisms, the team behind the Experience Corps^®^ papers (Brydges et al., 2021; Carlson et al., 2015; Fried et al., 2004, 2013; Parisi et al., 2015; Tan et al., 2006) should be commended for being one of the few groups [alongside Jongenelis et al.(2022b), Pettigrew et al. (2020), Warner et al. (2024), and Yeung et al. (2025)], to have successfully conducted RCTs in this field. Limitations of this review include that there are included papers describing RCTs that overlap from the same parent trials. This may have led to a risk of inadvertently over-counting data and misrepresenting the results. This risk was mitigated by not pooling data and by transparently reporting when papers stemmed from the same parent trial. Furthermore, we were unable to investigate results in further detail other than how they were presented by the authors, for example, Bjälkebring et al. (2021) reported decreased life satisfaction with “higher levels” of volunteering, without stating specifically the hours that “higher levels” refers to. This review is not a summary of all harms, as some harms have not yet been investigated, for example, the impact of a volunteer receiving negative feedback from a supervisor. Majority of the included papers were in English, so it is possible that generalizability of the findings of this review may be limited. Different cultures may find different benefits or harms from volunteering that may not have been captured in this review. The results may not be representative of the full extent of benefits and harms. This may be due to obsequious response bias, where respondents provide answers, they perceive the researcher wants to hear, and due to survival bias, a selection bias where people who did not engage with the programs also do not agree to providing follow-up information that may reveal an experienced harm as a reason for their volunteering cessation.

Future research of older adult volunteering programs should focus on strengthening the research designs and elevating the level of evidence underpinning this field. A stronger evidence base may provide more persuasive arguments to policy-makers to support funding of programs enabling increased participation in older adult volunteering. Future research may build on this review by further synthesizing and analyzing the benefits and harms where there were conflicting results. Future research should report all outcomes from a single trial in one publication, rather than dividing or repeating outcomes across multiple individual publications, to avoid the inadvertent double-counting of data upon review. Economic evaluation of older adult volunteering programs may be beneficial for strengthening arguments for funding support. Future measurement of potential harms is important so that volunteer programs can be designed to better mitigate these risks and identify those individuals who may be more likely to experience potential harms. Further research of the barriers and enablers to older adult volunteering would also assist in the development of programs that could be scaled to maximize participation and benefits.

## Conclusion

Volunteering appears to be largely associated with a wide range of health benefits. Potential harms may be influenced by the type of volunteering and hours spent volunteering. Despite discourse about benefits of volunteering, RCTs investigating these benefits are limited. To add to the multiple quasi-experimental and nonexperimental studies suggesting volunteering is beneficial to older adults’ health, there is a need for additional high-quality experimental studies to substantiate current recommendations for older adults to volunteer. To maximize the apparent health benefits there is also need to investigate ways of maximizing older adult participation in volunteering.

## Supplementary Material

gnag043_Supplementary_Data

## Data Availability

Data will be made available upon contact with the corresponding author. This review was pre-registered on PROSPERO crd.york.ac.uk/prospero/display_record.php? RecordID=456281.
