# Supplementary material for: Health benefits and harms of older adult volunteering: mixed methods systematic review
Source: Gerontologist. 2026 Apr 6;66(5):gnag043. doi: 10.1093/geront/gnag043 (PMC13131962; doi:10.1093/geront/gnag043)
Supplement: gnag043_Supplementary_Data [file gnag043_supplementary_data.pdf]

Health Benefits and Harms of Older Adult Volunteering: Mixed Methods Systematic Review

Mr. Nathan Williams (Corresponding Author), GCert in Higher Education, School of Primary and Allied Healthcare, Level 3, Building G, Monash University, Peninsula Campus, Moorooduc Highway, Frankston, Victoria, Australia, 3199. Ph: 03 1800 666 274. Email: nathan.williams@monash.edu, ORCID 0009-0005-5277-7668.

Dr. Marcelo Maghidman, PhD, Department of Social Work, Monash University, Caulfield, Victoria, Australia, marcelo.maghidman@monash.edu, ORCID 0000-0002-2040-055X.

Dr. Dai Pu, PhD, School of Primary and Allied Healthcare, Monash University, Frankston, Victoria, Australia, debbie.pu@monash.edu, ORCID 0000-0002-6268-4378.

Dr. Debra Mitchell, PhD, School of Primary and Allied Healthcare, Monash University, Frankston, Victoria, Australia, debra.mitchell@monash.edu, ORCID 0000-0002-9343-075X.

Prof. Terry Haines, PhD, School of Primary and Allied Healthcare, Monash University, Frankston, Victoria, Australia, terry.haines@monash.edu, ORCID 0000-0003-3150-6154.

Supplementary Materials

Supplementary Material 1 – Search Strategy

**Population**	
1	(Older adj3 (adult* or person* or people* or man or woman or men or women or volunteer*)).mp. [mp=title, book title, abstract, original title, name of substance word, subject heading word, floating sub-heading word, keyword heading word, organism supplementary concept word, protocol supplementary concept word, rare disease supplementary concept word, unique identifier, synonyms, population supplementary concept word, anatomy supplementary concept word]
2	aged.mp.
3	elderly.mp.
4	ageing.mp.
5	aging.mp.
6	geriatric.mp.
7	senior.mp.
8	seniors.mp.

9	late* life.mp.
10	exp Aged/
11	exp Aging/
12	exp Geriatrics/
**Intervention**	
13	(Volunt* adj1 work*).mp.
14	volunteering.mp.
15	volunteerism.mp.
16	(volunt* adj1 program*).mp.
17	"social good".mp.
18	"purposeful activity".mp.
19	"meaningful activity".mp.
20	altruis*.mp.
21	Volunteers/
22	Hospital Volunteers/
23	Altruism/
**Outcomes**	
24	benefit*.mp
25	risk*.mp.
26	enable*.mp.

27	barrier*.mp.
28	drive*.mp.
29	obstacle*.mp.
30	"quality of life".mp.
31	"health status".mp.
32	"personal satisfaction".mp.
33	motivation.mp.
34	loneliness.mp.
35	"mental health".mp.
36	mortality.mp.
37	"life expectancy".mp.
38	depression.mp.
39	"psychosocial functioning".mp.
40	"falls".mp.
41	"balance".mp.
42	"fitness".mp.
43	"endurance".mp.
44	"strength".mp.
45	frailty.mp.
46	self concept.mp.

47	memory.mp.
48	cognition.mp.
49	pain.mp.
50	anxiety.mp.
51	emotions.mp.
52	well-being.mp.
53	exp Risk/
54	exp "Quality of Life"/
55	exp Health Status/
56	exp Personal Satisfaction/
57	Motivation/
58	Loneliness/
59	Mental Health/
60	Mortality/
61	exp Life Expectancy/
62	Depression/
63	Psychosocial Functioning/
64	Accidental Falls/
65	exp Postural Balance/
66	exp Physical Fitness/
67	exp Physical Endurance/

68	exp Muscle Strength/
69	Frailty/
70	exp Self Concept/
71	exp Memory/
72	exp Cognition/
73	Social Cognition/
74	Social Isolation/
75	Social Identification/
76	Pain/
77	Anxiety/
78	exp Anxiety Disorders/
79	exp Emotions/
80	1 or 2 or 3 or 4 or 5 or 6 or 7 or 8 or 9 or 10 or 11 or 12
81	13 or 14 or 15 or 16 or 17 or 18 or 19 or 20 or 21 or 22 or 23

82	24 or 25 or 26 or 27 or 28 or 29 or 30 or 31 or 32 or 33 or 34 or 35 or 36 or 37 or 38 or 39 or 40 or 41 or 42 or 43 or 44 or 45 or 46 or 47 or 48 or 49 or 50 or 51 or 52 or 53 or 54 or 55 or 56 or 57 or 58 or 59 or 60 or 61 or 62 or 63 or 64 or 65 or 66 or 67 or 68 or 69 or 70 or 71 or 72 or 73 or 76 or 77 or 78 or 79
83	80 and 81 and 82

Supplementary Material 2 – Characteristics of Included Papers

Grades of Evidence (Commonwealth of Australia, 2009; Melnyk & Fineout-Overholt, 2022):

I - Evidence obtained from a systematic review of all relevant randomised control trials.

II - Evidence obtained from at least one well designed randomised control trial.

III - Evidence obtained from well-designed controlled trials without randomisation.

IV - Evidence obtained from well-designed cohort studies, case control studies, interrupted time series with a control group, historically controlled studies, interrupted time series without a control group or with case- series

V - Evidence obtained from systematic reviews of descriptive and qualitative studies

VI - Evidence obtained from single descriptive and qualitative studies

VII - Expert opinion from clinicians, authorities and/or reports of expert committees or based on physiology

**Authors (Year)**	**Design, Grade of Evidence (I – VII)**	**Outcome and Measure**	**n, Participants**	**Setting, Country**	**Age, Mean (SD) when provided or proportion (%)**	**Gender, Women (%)**	**Time Point**	**Volunteering Intervention**	**Quotes/ Notes**
Abe et al. (2022)	Cohort, IV	- Physical activity, time spent walking<br><br>- Healthy eating habits, Dietary Variety Score<br><br>- Intellectual activity, four	6 168	Community, Japan	73.5 (5.3)	50.7	2 years	Volunteer groups	

		items form the TMIG-IC							
Abe et al. (2023)	Cohort, IV	- Disability Incidence, Long Term Care Insurance System (level of nursing support)<br><br>- All cause Mortality	9 090, frail older adults	Community, Japan	76.0 (6.6)	55.1	6 years	Volunteer clubs	
Akhter-Khan et al. (2023)	Cohort, IV	- Loneliness, Modified GHQ and CES-D	3 572, no initial reported loneliness	Community, Indonesia	60.0 (5.39)	52.8	15 years	Volunteer labor e.g. cleaning local village	
Ayalon (2008)	Cohort, IV	- Mortality rate, year of death from National Deaths Record	5 055	Community, Israel	Volunteers: All aged 60+ years, majority 60 to 69 years (45.7%)<br><br>Non-volunteers: All aged 60+ years, majority 60 to 69 years (47.0%)	Volunteers: 51.9<br><br>Non-volunteers: 56.5	7 years	Any ongoing volunteering, e.g. Israeli equivalent of Salvation Army, Citizen's Police	
Barron et al. (2009)	Cohort, IV	- Walking distance, number of blocks walked per week<br><br>- Energy levels, rated 0 to 10	174	Elementary schools, United States	Demographic data divided by health status:	Excellent/ Very Good: 87.9<br><br>Good: 92.6	1 year	Assisting in elementary schools	

		- Grip strength, dynamometer<br><br>- Sit to stand speed, 5 times sit to stand test<br><br>- Walking speed, timed 4m walk at usual pace<br><br>- Stair climbing speed, time taken to walk up and down a flight of stairs			Excellent/ Very Good: 68.1 (6.5)<br><br>Good: 69.2 (6.4)<br><br>Fair/ Poor: 70.0 (8.0)	Fair/ Poor: 90.5			
Bell et al. (2022)	Cohort, IV	- CRP levels, blood test	5 540	Community, United States	66.1 (9.8)	41.7	4 years	Any religious, educational, health-related or charitable volunteering in past year	
Bell & Ferraro (2025)	Cohort, IV	- Heart attack risk, no. of months before incidence of first heart attack	3 676, no previous cardiac disease	Community, United States	67.0 (9.79)	59.81	12 years	Any religious, educational, health-related or charitable volunteering in past year	
Bjalkebring et al. (2021)	Cohort, IV	- Life satisfaction, Satisfaction with Life Scale	5 913	Community, Sweden	63.3 (1.6)	56.8	4 years	Voluntary work/ volunteering	
Breheny et al. (2020)	Interview, VI	- Attitude towards aging, see quotes	6	Community, New Zealand	Range: 68 to 90 years	83.3	Not stated	Age Concern – volunteer visiting service to other older adults	One participant said they "visit two 95-year-olds and they live in

									their own home and I visit them once a week for one and half hours and they're delightful. You know, how many 95-year olds live in their own home? With no help…”
Brown et al. (2009)	Mixed Methods, IV	Health, SF-12 and interviews<br><br>Physical health, SF-12<br><br>Physical function, SF-12<br><br>Social Connectedness, SF-12 and interviews<br><br>Mental health, SF-12<br><br>Pain, SF-12	40	Community, United States	68.6 (6.3)	40.0	1 week	Habitat for Humanity – building new homes and home maintenance	“I've had a lot of problems. But I'm still healthy.”<br><br>“I couldn’t do much of the heavy work. I just socialized.”
Brydges et al. (2021)[a]	RCT, II	- Cognitive function, Stroop task	Volunteering: 181<br><br>Control: 155	Elementary schools, United States	Volunteering: 661. (5.5)<br><br>Control: 66.7 (5.8)	Volunteering: 81.2<br><br>Control: 82.3	1 to 2 years	Volunteering: Experience Corps program – assisting elementary school children in the classroom, 15	

								hours per week for 2 years<br><br>Control: Low activity volunteering programs, e.g. city festival or event, shorter-term commitment	
Burr et al. (2016)	Cohort, IV	- Hypertension risk, sphygmomanometer<br><br>- Central adiposity, waist circumference<br><br>- CRP levels, blood test<br><br>- Lipid dysregulation, blood test	7 803	Community, United States	68.6 (10.0)	57.2	2 years	Any religious, educational, health-related or charitable volunteering in past year	
Cao et al. (2021)	Focus Group, VI	- Stress/ Anxiety, see quotes	70, low income culturally diverse people	Community, United States	76.8 (8.5)	60.0	Not stated	Formal volunteering	“He asks a lot of information about Bhutan and he shares with us so that will also help us to cope with the stress. We are interested in talking with our friends. It helps us to laugh and

		- Personal growth							to manage our stress.”<br><br>“I learned about transportation in this group, I have been here for two years now.”
Carlson et al. (2015)[a]	RCT, II	- Brain and hippocampus volume, MRI	Volunteering: 65<br><br>Control: 58	Elementary schools, United States	Volunteering: 67.7 (6.2)<br><br>Control: 66.7 (5.9)	Volunteering: 72.6<br><br>Control: 72.7	2 years	Volunteering: Experience Corps program – assisting elementary school children in the classroom<br><br>Control: Low activity	
Carney et al. (1987)	Cohort, IV	- Self-Purpose, Purpose in Life Scale	15	Elementary school, United States	Not stated	Not stated	9 weeks	Assisting elementary school students in the classroom	
Carr et al. (2018)	Cohort, IV	- Loneliness, 3 items from the Leave Behind Questionnaire	5 882, widowed	Community, United States	67.9 (8.4)	53.2	8 years	Any religious, educational, health-related or charitable volunteering in past year	
Celdran & Villar (2007)	Cross Sectional, IV	All single item questions with Likert scale responses:<br><br>- Self-fulfilment	88	Community, Spain	68.6 (8.4)	29.5	2 weeks	Care services for older adults, museum guides, teachers, retired managers mentoring young entrepreneurs	

		- Feeling more physically active<br><br>- Feeling useful<br><br>- Feeling ‘tied up’<br><br>- Volunteering feeling too effortful							
Chang et al. (2022)	Cross Sectional, IV	Happiness, single question rated 0 to 100<br><br>Health, single question rated 0 to 100<br><br>Physical health, SF-12 version 2<br><br>Mental health, SF-12 version 2	3 692	Community, Taiwan	76.2 (6.5)	56.7	4 years	Community volunteering in the past month	
Chen (2016)	Interview, VI	- Cognitive function, see quotes	31	Community, Taiwan	Range 60 to 93 years, majority 60 to 69 years (41.9%)	45.2	Not stated	Volunteering regularly for at least 2 years	“My brain will degenerate slowly (as opposed to quickly) because it is used whenever I do my volunteer job and learn how to resolve problems there.”

		- Self-Purpose<br><br><br><br><br><br><br><br><br><br><br><br>- Personal growth<br><br><br><br><br><br><br><br><br><br><br><br><br><br><br><br><br><br><br><br><br><br><br>- Attitude towards aging							Being able to use pre-existing skills provides volunteers with a sense of purpose<br>- “I used to study mechanical engineering… so I learned how to use these tools.”<br><br>"In the classroom… you will forget soon if you don’t use what you were just taught. There is no such problem for what I learn in my volunteer work.”<br><br>“I learned and accumulated many skills about how to build strong bridges.”<br><br>“…because you are a volunteer, you have to learn a lot.”<br><br>“Many disabled persons I call to greet on the phone are

		- Compassion for others							younger than me and live in an institution. Interacting with them makes me think a lot about my later life."<br><br>"Looking at (those more unwell), I am lucky because I am still healthy."<br><br>"(I) have grown to be more empathetic."
Cheung & Kwan (2006)	Cross Sectional, IV	- Self-Esteem, 5 questions with Likert scale responses<br><br>- Life satisfaction, 5 questions with Likert scale responses<br><br>- Health, 3 questions with Likert scale responses	719	Community, Hong Kong	79.9 (SD not provided)	79.9	Several months in 1998	Volunteering at a social service center	
Chiao (2019)	Cohort, IV	- Cognitive status, Short Portable Mental Status Questionnaire	899	Community, Taiwan	All aged 60+ years. Age for volunteering group not stated	Not stated for volunteering group	14 years	Volunteer work. This study presented results for both volunteering and social participation	

								separately, however demographic data were combined	
Cho & Xiang (2023)	Cohort, IV	- Loneliness, UCLA-3	5 000	Community, United States	74.7 (0.2)	56.4	12 years	Any religious, educational, health-related or charitable volunteering in past year	
Chu & Koo (2023)	Cross Sectional, IV	- Life satisfaction, Satisfaction with Life Scale	186	Community, Taiwan	All 65+ years, majority 65 to 69 (38.2%)	78.5	1 month	Any formal volunteering	
Cohen-Mansfield & Jensen (2017)	Interview, VI	- Happiness	48	Elementary and all-aged schools, Israel	71.4 (5.9)	58.3	Not stated	Assisting elementary and all-aged school students in the classroom (Senior Volunteer Programs)	89% of respondents reported feeling more joy after attending the program<br><br>One individual reported feeling upset at the end of the program, "the child will leave, and it will bother me that I will never see him again." (Harm)
Connolly & O'Shea (2015)	Cross Sectional, IV	- Social connectedness, single item question wit	261	Community, Ireland	All aged 55+ years. 23% aged 75+ years	77.0	6 weeks	Senior Helpline – trained older adult volunteers offer a telephone listening	

		Likert scale responses<br><br>- Self-worth, two questions with Likert scale responses<br><br>- Quality of Life, two questions with Likert scale responses<br><br>- Health, single item question wit Likert scale responses						service to other older adults<br><br>Fáilte Isteach – conversational English classes with immigrants	
Correa et al. (2022)	Cohort, IV	- Cognitive functioning, MMSE, Clock Drawing Test, Verbal Fluency, CERAD Word List Recall	291	Community, Brazil	71.3 (6.2)	89.0	2 years	Volunteering for formal organizations and for the benefit of people outside of participants' nuclear family in the past month	
Costenoble et al. (2023)	Cohort, IV	- Frailty, Fried Frailty Phenotype	322, not frail	Community, Belgium	83.04 (2.78)	58.0	1 year	Any of ten volunteering categories	
Davila (2018)	Cross Sectional, IV	- Frequency of social contact, single item with Likert scale responses<br><br>- Size of social network, single	2 057	Community, Spain	75.6 (7.2)	53.7	2011	Any volunteer or charity work	

		item with responses 0 to 7							
De Souza et al. (2011)	Quasi-Experimental, III	- Physical health, WHO QOL-Bref<br><br>- Psychological wellbeing, WHO QOL-Bref<br><br>- Social relations, WHO QOL-Bref<br><br>- Environmental QOL, WHO QOL-Bref<br><br>- Global QOL, WHO QOL-Bref	Volunteers: 166<br><br>Control: 33	Community, Brazil	Volunteers: 68.2 (5.9)<br><br>Control: 70.8 (6.5)	Volunteers: 87.3<br><br>Control: 78.8	6 months	Volunteering through Non-Governmental Organizations	
Dulin et al. (2012)	Cohort, IV	- Happiness, two items from Lyubomirsky and Lepper's measure of subjective happiness	1 028	Community, New Zealand	63.0 (4.1)	Not stated	2 years	Any volunteer activity	
Fields et al. (2023)	Interview, VI	- Loneliness<br><br>- Social Connectedness	18, trained callers	Community via phone, United States	73.2 (5.9)	100.0	12 weeks	Phone calling service to other older adults during COVID-19	"They will be helping me so I won't be lonely either, 'cause I live alone."<br><br>"I would like having a friend…my age and we could laugh and talk about how we

		-Self-Purpose<br><br>-Personal growth							were raised and how we behaved when we were growing up.”<br><br>“It gave me something to do. Gave me something very constructive, and I feel like it was very important.”<br><br>“That experience…taught me something too.”
Fletcher et al. (2025)	Cohort, IV	- Episodic Memory, SENAS	2 245, no diagnosis of dementia or poor health	Community, United States	74.1 (8.0)	63.0	1 year	Any religious, educational, health-related or charitable volunteering in past year	
Fraser et al. (2009)	Mixed Methods, IV	- Social connectedness, see quotes<br><br>- Self-Esteem, Collective Self Esteem Scale	21	Bronx Zoo, United States	60.0 (SD not stated)	Not stated	2006	Exhibit Explainers or Tour Guides at Bronx Zoo, New York	“It’s like a family…an extended family.”<br><br>“We love each other’s company. We see each other outside of the zoo too. We have become brothers and sisters.”

Fried et al. (2004)[a]	RCT, II	- Cognitive activity, questionnaire items including number of books, number of high/ medium/ low cognitive activities completed in the past month, hours of television watched<br><br>- Physical activity, grip strength, walking speed, number of blocks walked per week, flights of stairs climbed per week<br><br>- Social support, number of people in social circle, number of people who would check on you, who you can rely on	Volunteering: 69<br><br>Control: 56	Elementary schools, United States	All aged 60+ years<br><br>Volunteering: Majority 71 to 75 years (30.9%)<br><br>Control: Majority 66 to 70 years (40.0)	Volunteering: 88.6<br><br>Control: 94.8	4 to 8 months	Volunteers: Experience Corps program – assisting elementary school children in the classroom<br><br>Control: Placed on waiting list and did not volunteer	
Fujii et al. (2024)	Cross Sectional, IV	- Life Satisfaction, LSS	4 498, healthy and independent	Community, Japan	73.6 (5.4)	52.8	Oct 2019 – Mar 2021	Any volunteering. Study investigated a range of productive activities and reported results according to each.	

Gagliardi et al. (2020)	Mixed Methods, IV	- Physical activity, Physical Activity Scale for the Elderly<br><br>- Positive and negative affect, Positive and Negative Affect Scale<br><br>- Life satisfaction, single item with Likert scale responses<br><br>- Social connectedness, Lubben Social Network Scale	19	Community, Italy	75.7 (5.1)	42.1	1 year	Environmental volunteering e.g. cleaning and maintenance in local parks	
Ge et al. (2025)	Cross Sectional, IV	- Life satisfaction, Single question from CLASS survey	9 828, no cognitive impairment	Community, China	Not provided. All participants aged 60+.	49.7	Data from 2020 survey	Eight types of community volunteering	
George & Singer (2011)	RCT, II	- Depression, Beck Depression Inventory<br><br>- Cognitive function, MMSE<br><br>- Stress/ Anxiety, Beck Anxiety Index<br><br>- Self-Purpose, single item with	Volunteering : 8<br><br>Control: 7<br><br>People with mild to moderate Dementia	The Intergeneration al School, United States	Volunteering : 85.7 (6.0)<br><br>Control: 81.4 (8.2)	Volunteering : 87.5<br><br>Control: 85.7	5 months	Volunteering: Mentors and classroom assistants for kindergarten and elementary school students, total 20 hours<br><br>Control: Attended educational seminars on	

		Likert scale responses<br><br>- Sense of usefulness, single item with Likert scale responses						healthy aging, total 12 hours	
Gil-Lacruz et al. (2019)	Cohort, IV	- Life satisfaction, single item with Likert scale responses<br><br>- Happiness, single item with Likert scale responses<br><br>- Health, single item with Likert scale responses	1 699	Community, Chile, Mexico, Spain	All aged 60+ years, majority aged 61 to 65 years (36%)	53.0	16 years	Volunteering in four categories: Social awareness, professional and political, education and leisure or religious	
Gonzales et al. (2019)	Cohort, IV	- Health, single item with Likert scale responses<br><br>- ADLs, number of tasks requiring assistance with<br><br>- IADLs, number of tasks requiring assistance with<br><br>- Depression, CES-D 8	682	Community, United States	71.7 (11.4)	64.5	2 years	Any religious, educational, health-related or charitable volunteering in past year	

Greenfield & Marks (2004)	Cross Sectional, IV	- Positive affect, participants were asked how frequently they felt cheerful, in good spirits, extremely happy, calm and peaceful, satisfied, and full of life. Likert scale responses.<br><br>- Self-Purpose, Ryff's Purpose in Life Index	373	Community, United States	69.7 (3.0)	57.0	1995	Formal volunteering in the past month in hospital, nursing home, or other healthcare-oriented volunteer work; school or other youth-related volunteer work; volunteer work for political organizations or causes; volunteer work for any other organization, cause, or charity	
Griep et al. (2017)	Cohort, IV	- Cognitive functioning, Copenhagen Psychosocial Questionnaire II<br><br>- Dementia incidence, presence or otherwise of anti-dementia treatment	7 222	Community, Sweden	67.0 (1.7)	50.3	4 years	Voluntary (unpaid work) in society, relief organization, religious organization, political party or non-profit organizations	
Guiney et al. (2021)	Cross Sectional, IV	- Cognitive functioning, MoCA<br><br>- Social connectedness, items from Thomas' social engagement scale	91	Community, New Zealand	69.8 (2.9)	69.0	Not stated	Volunteering at least once per month	

		- Physical activity, New Zealand Physical Activities Questionnaire Short Form							
Hambisa et al. (2022)	Cohort, IV	- Continued driving ability, single item with driving or not driving response	4 055, women	Community, Australia	86.7 (Not stated)	100.0	5 years	Volunteer work for any community or social organizations e.g. community welfare, fundraising	
Han et al. (2017)	Cohort, IV	- Cardiovascular disease-related incidents (e.g. heart attack, stroke), self-reported and exit interviews<br><br>- Mortality, exit interviews	10 841	Community, United States	67.1 (9.6)	60.6	4 years	Any religious, educational, health-related or charitable volunteering in past year	
Han et al. (2019)	Interview, VI	- Pleasure, see quotes<br><br>- Social connectedness	8	Community, United States	73.1 (5.7)	75.0	1 month	Stepping Stones – assisting with activities for people with Dementia	“It just makes me feel complete”<br><br>“Having social interaction is good for me especially after retirement… it’s a social thing”

		- Self-Purpose<br><br>- Compassion for others<br><br>- Self-Compassion							“It gave me something fun to do in my retirement that’s been important”<br><br>“It makes me feel like I’ve done something worthwhile that afternoon.<br><br>“Volunteering in the program made me extremely patient with old people and myself”
Han et al. (2020)	Cohort, IV	- Cognitive functioning, Telephone Interview for Cognitive Status	9 697	Community, United States	62.1 (6,9)	54.5	16 years	Any religious, educational, health-related or charitable volunteering in past year	
Han & Zhang (2025)	Interview, VI	- Social Connectedness	25	Community via Zoom, United States	71.8 (5.19)	52.0	During COVID-19	Any formal volunteering	“Got to meet people.”<br>“I think that's (volunteering is) how I met people when I came back here.”
Han & Park (2024)	Cohort, IV	- Frailty, Authors’ own constructed frailty index	34 986	Community, United States	65.4 (9.8)	Not provided	22 years	Any religious, educational, health-related or charitable	

								volunteering in past year	
Harris & Thoresen (2005)	Cohort, IV	- Mortality rate, data from National Death Index	7 527	Community, United States	76.8 (Not stated)	62.0	6 years	Any volunteer work such as helping in charity work, working in a shop for a non-profit organization, working in a hospital or nursing home or doing community work without pay	
Hayward & Krause (2014)	Cohort, IV	- Functional disability, 15 item ADL and IADL Index described by the authors	1 152, religious leaders	Community, United States	77.5 (6.2)	63.1	4 years	Voluntary lay leadership within a church	
Hidalgo et al. (2013)	Cross Sectional, IV	- Life satisfaction, Scale of Satisfaction with Life<br><br>- Health, SF-36<br><br>- Social connectedness, SF-36<br><br>- Social support, single item question ranked from minimum to maximum support from family and friends	176, members of Federation of Analdusian Organization for Senior Citizens	Community, Spain	64.6 (10.0)		1 month	Current volunteer at the Federation of Analdusian Organization for Senior Citizens or any other organization	

		- Self-Esteem, Rosenberg's Scale of Self-Esteem<br><br>Happiness, single item question with Likert scale responses							
Ho et al. (2018)	Cross Sectional, IV	- Physical activity, average daily physical activity level on wrist accelerometry	738	Community, United States	71.9 (7.2)	54.0	1 year	Volunteering	
Hong & Morrow-Howell (2010)	Quasi-experimental, III	Depression, CES-D 9<br><br>Health, single item with Likert scale responses<br><br>Physical function, multiple items of function (e.g. ease of walking, carrying, climbing stairs) with Likert scale responses	Volunteers: 167<br><br>Control: 167	Elementary schools, United States	Volunteers: 64.8 (7.4)<br><br>Control: 65.1 (7.2)	Volunteers: 86.2<br><br>Control: 83.9	2 years	Volunteers: Experience Corps program – assisting elementary school children in the classroom<br><br>Control: Matched participants from Health and Retirement study	
Hsiao et al. (2020)	Quasi-experimental, III	- Self-compassion, Self-Compassion Scale	Volunteering: 36<br><br>Control: 36	Community, Taiwan	Volunteering: 71.5 (7.3)<br><br>Control: 72.8 (7.7)	Volunteering: 75.0<br><br>Control: 92.0	1 year	Environmental volunteering e.g. recycling stations	

		- Compassion for others, Compassion Scale<br><br>- Depression, Taiwan Geriatric Depression Scale<br><br>- Happiness, Chinese Happiness Index<br><br>- Physiological health, medical records containing height, weight, blood pressure, and laboratory results such as blood test, total cholesterol, low-density lipoprotein cholesterol, triglycerides, blood glucose, C-reactive protein, and cortisol							
Huang (2019)	Cross Sectional, IV	- Life satisfaction, one item ranked 1 to 10<br><br>- Happiness, one item with Likert scale responses	1 076	Community, 5 countries in Asia	All above 65 years	Not stated	1 year	Active member of a voluntary organization, for example, educational, religious, environmental	

		- Health, one item with Likert scale responses							
Hung et al. (2022)	Cross Sectional, IV	- Physical activity, wrist accelerometry<br><br>- Grip strength, average of 2 hand dynamometer measures	113, able to walk independently and volunteering at least 1 hour per week<br><br>Participants were divided into two groups: Non-intensive (< 15 hrs per week): 52<br><br>Intensive (16+ hrs per week): 61	Community, Taiwan	Non-intensive (< 15 hrs per week): 70.7 (7.3)<br><br>Intensive (16+ hrs per week): 73.6 (6.9)	Non-intensive (< 15 hrs per week): 78.8<br><br>Intensive (16+ hrs per week): 73.8		Community environmental volunteering, including collecting waste, categorizing the garbage, cleaning and repairing mendable objects, and tearing down unfixable objects, selling repaired objects for charity	
Hunter & Linn (1981)	Quasi-Experimental, III	- Life satisfaction, Life Satisfaction Scale<br><br>- Depression, Items taken from analyses by Derogatus et al.<br><br>- Anxiety, Items taken from analyses by Derogatus et al.	Volunteers: 53<br><br>Non-volunteers: 49	Community, United States	Volunteers: 74.0 (6.4)<br><br>Non-volunteers: 73.5 (5.5)	Volunteers, Mean (SD): 1.6 (0.5)<br><br>Non-volunteers, Mean (SD): 1.4 (0.5)<br><br>(Where male =1, female = 2)		Volunteering at Veterans Administration Hospital, mean 15 hours per week	

		- Self-esteem, Items from Rosenberg, Coopersmith and Brown’s scales<br><br>- Will to live, Ellison’s Will to Live Scale							
Huo et al. (2021)	Cohort, IV	- Depression, CES-D 8<br><br>- Self-Perception of Aging, Attitudes Towards Aging subscale of the Philadelphia Geriatric Center Morale Scale	9 017	Community, United States	74.2 (6.6)	59.0	4 years	Any religious, educational, health-related or charitable volunteering in past year	
Huo, Kim & Wang (2023)	Cohort, IV	- Depression, CES-D 8<br>- Life Satisfaction, Likert scale 1 to 5	1 074, bereaved parents	Community, United States	68.2 (10.5)	65.0	2 years	Any religious, educational, health-related or charitable volunteering in past year	
Huo, Kim & Haghighat (2023)	Cohort, IV	- Self-Perception of Aging, 8 items from Attitude Towards Aging Scale	10 183, Black and White older adults	Community, United States	Black people: 63.4 (9.1)<br><br>White people: 67.8 (9.5)	Black people: 66.0<br><br>White people: 59.0	8 years	Any religious, educational, health-related or charitable volunteering in past year	

Huo & Kim (2022)	Cohort, IV	- Life satisfaction, 5 items with Likert scale responses<br><br>- Attitude towards aging, 8 items taken from Attitude Toward Own Aging Scale	10 441	Community, United States	Volunteers: 66.4 (8.9)<br><br>Former Volunteers: 68.2 (10.6)<br><br>Non-Volunteers: 68.0 (10.1)	Volunteers: 60.0<br><br>Former Volunteers: 64.0<br><br>Non-Volunteers: 57.0	10 years	Any religious, educational, health-related or charitable volunteering in past year	
Hwan & Hussin (2022)	Interview, VI	- Happiness<br><br>- Health<br><br>- Stress/ Anxiety	10, people with End-Stage Renal Disease	Community, Malaysia	67.4 (4.6)	30.0	Not stated	Volunteering for more than 3 months, e.g. fundraising, recycling	Volunteering "is very good, and working together, everyone is very happy"<br><br>"I think my health is improving since I started volunteering"<br><br>"Doing the recycling job makes me relax."
Ide et al. (2023)	Cohort, IV	- Functional decline, Certification for insurance benefits eligibility	51 968	Community, Japan	74.0 (6.0)	53.6	6 years	Any volunteering	
Infurna et al. (2016)	Cohort, IV	- Cognitive impairment prevalence, Telephone Interview for Cognitive Status	13 262	Community, United States	71.3 (8.3)	58.0	14 years	Any religious, educational, health-related or charitable volunteering in past year	

Jiang et al. (2019)	Cohort, IV	- Life satisfaction, Satisfaction with Life Scale	1 591	Community, Australia	66.6 (1.5)	48.0	8 years	Volunteering in the past 6 months	
Jiang (2022)	Cohort, IV	- Depression, CESD-9	3 128	Urban and Rural Communities, China	Urban: 70.9 (2.5)<br><br>Rural: 70.6 (2.1)	Urban: 44.6<br><br>Rural: 39.4	2 years	Any formal volunteering, for example, charity or environmental causes	
Jirovec & Hyduk (1999)	Cohort, IV	- Mortality, All cause mortality at follow-up<br><br>- Number of chronic conditions, total number of self-reported conditions<br><br>- Cognitive function, TICS-M<br><br>- Physical function, >3 self-reported limitations with physical function (e.g. walking a flight of stairs) or activities with daily living (e.g. dressing, bathing)<br><br>- Pain, single yes/no question	12 998	Community, United States	68.1 (9.6)	58.1	8 years	Any religious, educational, health-related or charitable volunteering in past year	

		- Self-rated health, Single question with Likert scale responses<br><br>- Physical activity frequency, Participants were asked their frequency of engaging in vigorous, moderate and light physical activity in the past year. 1 or more occasions of vigorous or moderate physical activity was considered frequent physical activity<br><br>- Sleep problems, 4-item Jenkins Sleep Questionnaire<br><br>- Positive affect, Self-rated 6-item scale<br><br>- Life satisfaction, 5-item Satisfaction with Life Scale							

		- Optimism, LOT-R<br>- Purpose in Life (Self-Purpose), Ryff's Psychological Wellbeing Scale<br><br>- Depressive symptoms, CESD-8<br><br>- Loneliness, UCLA Loneliness Scale<br><br>- Frequency of social contact, self-reported frequency of meeting, phoning, or writing/emailing children, other family, or friends							
Johnson (2013)	Cohort, IV	- Health, single item with Likert scale responses<br><br>- Depression, CES-D 8	8 659, widowed people, results also reported as widowed and recently widowed (within the last 2 years)	Community, United States	Female: 71.0 (6.5)<br><br>Male: 71.6 (6.8)	46.9	6 years	Any religious, educational, health-related or charitable volunteering in past year	

Jones & Reynolds (2019)	Interview, VI	- Social connectedness<br><br>- Attitude towards aging<br><br>- Psychological wellbeing	6	Community, England	65.2 (3.4)	83.3	Not stated	Charity shop volunteering	"I just like the…interaction with other people as opposed to just what would amount to spending quite a few days a week just on my own at home."<br><br>"(When volunteering) you look at life differently, you just thank the Lord [for] what you've got … you feel younger as well."<br><br>"I feel better in myself, making your brain work isn't it?"
Jongenelis, Jackson, Newton et al. (2022)[b]	RCT, II	- Depression, CES-D 20<br><br>- Psychological wellbeing, Warwick-Edinburgh Mental Wellbeing Scale	Volunteering: 201<br><br>Control: 244	Community, Australia	70.4 (6.1)<br><br>(Demographic data for each group not presented)	56.0<br><br>(Demographic data for each group not presented)	6 months	Volunteering: Any type of formal volunteering, minimum 1 hour per week<br><br>Control: Not asked to volunteer, not required not to volunteer	

		- Self-Esteem, Rosenberg Self-Esteem Scale<br><br>- Self-Efficacy, General Self-Efficacy Scale<br><br>- Self-Purpose, Ryff's Psychological Wellbeing Scale<br><br>- Personal Growth, Ryff's Psychological Wellbeing Scale<br><br>- Life satisfaction, single item with Likert scale responses<br><br>- Social connectedness, Social Provisions Scale							
Jongenelis, Jackson, Warburton et al. (2022)	Cohort, IV	- Psychological wellbeing, CES-D 20, Warwick-Edinburgh Mental Wellbeing Scale<br><br>- Eudemonic wellbeing, Ryff's Psychological Wellbeing Scale	108	Community, Australia	69.9 (Not stated for full sample)	64.0	6 months	Any formal volunteering	

		- Psychological resources, Rosenberg’s Self-Esteem Scale, General Self-Efficacy Scale, Social Provisions Scale							
Jung et al. (2023)	Cross Sectional, IV	- Quality of Life, CASP-12	1 741	Community, Singapore	69.9 (7.2)	52.9	2016 to 2017	Unpaid help to any groups, clubs or organizations, such as by raising money, organizing events, visiting people, secretarial work, campaigning, and more in the past 12 months	
Kail & Carr (2020)	Cohort, IV	- Cognitive functioning, Telephone Interview for Cognitive Status and a single item on self-rated memory with Likert scale responses	27 485	Community, United States	66.7 (9.9)	60.0	16 years	Any religious, educational, health-related or charitable volunteering in past year	
Kim, Halvorsen, et al. (2025)	Cohort, IV	- Epigenetic age acceleration, Five DNA methylation measures	2 605	Community, United States	74.9 (7.4)	57.0	4 years	Any religious, educational, health-related or charitable volunteering in past year	

Kim, Shiba, et al. (2025)	Cohort, IV	- Hypertension prevalence, Blood pressure readings	18 847	Community, United States	65.6 (10.6)	57.0	10 years	Any religious, educational, health-related or charitable volunteering in past year	
Kim et al. (2020)	Cohort, IV	- Mortality, exit interviews and National Death Index<br><br>- Incidence of chronic conditions, self-reported diagnoses<br><br>- Overweight/ obesity, BMI<br><br>- Cognitive functioning, TICS-M<br><br>- Physical functioning, recorded as having physical limitations with 4 or greater functional activities e.g. climbing one flight of stairs, or ADLs e.g. dressing.	12 998, divided into time spent volunteering<br><br>50 to 99 hrs per year: 1 150<br><br>100+ hrs per year: 1 990	Community, United States	50 to 99 hrs per year: 67.6 (9.5)<br><br>100+ hrs per year: 68.4 (9.0)	50 to 99 hrs per year: 63.0<br><br>100+ hrs per year: 61.1	8 years	Any religious, educational, health-related or charitable volunteering in past year	Note: 0 – 49 hrs per week group had insignificant results for all outcomes, so only data on the other two groups are presented.

		- Pain, single item with dichotomous response<br><br>- Health, single item with Likert scale responses<br><br>- Binge drinking, single item with number of days of 4 or more alcoholic drinks in the past three months<br><br>- Smoking, single item with dichotomous response<br><br>- Physical activity, at least one occasion per week of moderate to vigorous physical activity<br><br>- Sleep problems, Jenkins Sleep Questionnaire<br><br>- Positive affect, 6 item scale from Midlife in the United States study							

		- Life Satisfaction, Satisfaction with Life Scale<br><br>- Purpose in Life, Ryff's Psychological Wellbeing scale<br><br>- Optimism, LOT-R<br><br>- Depression, CESD-8<br><br>- Hopelessness, 4 items with Likert scale responses<br><br>- Loneliness, UCLA Loneliness Scale<br><br>- Frequency of social contact, number of times per week participants met, wrote or phoned family or friends							
Kim & Pan (2025)	Cross Sectional, IV	- Cognitive performance, CERAD Word List and Praxis, Brave man story from the East Boston Memory	2 293	Community, United States	75.3 (7.2)	57.0	1 year, 4 months	Any religious, educational, health-related or charitable volunteering in past year	

		Test, Logical Memory from the Wechsler Memory Scale Fourth Edition (WMS-IV), Animal Fluency, and TICS							
Kim & Yoon (2020)	Cohort, IV	- CRP concentration, blood test<br><br>- Sleep quality, single item on sleep duration, sufficient sleep measured as at least 6 hours sleep on average	1 124	Community, United States	69.3 (7.9)	52.0	5 years	Any religious, educational, health-related or charitable volunteering in past year	
Klinedinst & Resnick (2014)	Cross Sectional, IV	- Depression, Patient Health Questionnaire-2<br><br>- Usefulness, single item with Likert scale responses<br><br>- Physical activity, Yale Physical Activity Survey	127, residents of continuing care retirement community, no cognitive impairment	Independent or assisted living areas of a continuing care retirement community, United States	87.8 (6.6)	77.2	2014	Volunteering internally or externally from the community, for example, delivering mail to residents, working on food committee, delivering meals on wheels outside the community	
Konrath et al. (2012)	Cohort, IV	- Mortality, measured as either alive or	10 317	Community, United States	69.2 (0.5)	51.6	4 years	Volunteering in the past 10 years	

		deceased at follow up							
Krause (2009)	Cohort, IV	- Health, two items with Likert scale responses, participants self-rated their current health and health one year ago	681, practicing Christians	Community, Coterminous United States	Not stated	Not stated	6 years	Christian church-based volunteering	
Kritz et al. (2021)	Interview, VI	- Social connectedness, see quotes<br><br>- Compassion for others	Volunteer Program Completers: 4<br><br>Extenders: 3	Community, Australia	Completers: Range 75 to 83 years<br><br>Extenders: Range 70 to 78 years	Completers: 75.0<br><br>Extenders: 100.0	4 months	Walking group peer leaders	"I have enjoyed it and getting to know a few more people, instead of staying by myself, because I will quite often stay by myself. So, it has made me come out."<br><br>"I think the sense of being able to help people, makes me feel good."
Kuang et al. (2023)	Cohort, IV	- Gait speed, 3 metre walk test<br><br>- Difficulty walking, self-reported difficulty walking "across the room" and "one block"	2 460	Community, United States	75.0 (7.1)	54.0	1 year, 2 months	Any volunteering. Low volunteering considered as volunteering < 3 times per year	

Labegalini et al. (2015)	Interview, VI	- Life satisfaction, see quotes<br><br>- Social connectedness<br><br>- Personal growth<br><br>-Happiness	12	Community, Brazil	68.0 (Not stated), Range 60 to 79 years	100.0	2 months	Pastoral de Criança – 'Children's Pastoral Care' – providing care and support to disadvantaged children	"…changed a lot my life for the better"<br><br>"…is very good, because it is a time to get out of the House, chat and meet people"<br><br>"I learned a lot"<br><br>"I feel happier by helping a person"
Lakomy (2023)	Cohort, IV	- Quality of Life, CASP-12 scale	13 525	Community, 11 European countries	65.6 (SD not stated)	62.9	4 years	Any volunteer or charity work	
Lam et al. (2023)	Cross Sectional, IV	- Life satisfaction, Satisfaction with Life Scale<br><br>- Depression symptoms, CESD-10<br><br>- Cognitive functioning, MoCA<br><br>- Hand grip strength, dynamometer	341	Community, Hong Kong	70.4 (7.3)	Not stated	Not stated	Fundraising, food preparation, driving, garden maintenance, management, teaching, clerical work, befriending, coaching, art media production, personal care	
Landry (2017)	Interview, VI	- Life satisfaction, see quotes<br><br>- Physical activity	24	Community, United States	74.8 (7.2)	62.5	3 months	Volunteering for charitable, religious or healthcare	"I find it very fulfilling."

		- Happiness<br><br>- Self-Purpose						organizations, for minimum 1 year, at least 12 times in the past year	“This keeps me active. Keeps my mind going and my emotions going and my physical body moving.”<br><br>"When you give back and you help people, it makes me feel good.”<br>“Happy, joyous and free. I enjoy it so much… I just love it.”<br><br>After retirement “I found I had nothing to do…I just needed something to do. It (volunteering) was a perfect thing.”<br>“It gives me a purpose.”<br>“Volunteering saved my life. I literally would be dead if I didn’t have this to do. (It is the) reason that I get up in the morning.”

		- Stress/ anxiety							"I was very nervous at first because you're dealing with death…things that are not pleasant." (Harm)
Larkin et al. (2005)	Mixed Methods, IV	- Life satisfaction, Life Satisfaction Rating Scale<br><br>- Positive affect, see quotes<br><br>- Self-Purpose, see quotes	16	Community and schools, United States	8 participants aged 55 to 66 years, 8 participants aged 67+ years	Not stated, authors report genders were balanced	Not stated	Youth mentoring	Qualitative themes included opportunities to:<br><br>"Renew positive emotions."<br><br>"Reinforce meaning through being appreciated and recognized as a valuable person."
K. Lee et al. (2021)	Cohort, IV	- Physical health, single item with Likert scale responses<br><br>- Self-Purpose, 7 items from Ryff's Psychological Wellbeing Scale<br><br>- Cognitive health, Telephone	472, cognitively impaired	Community, United States	73.3 (6.3)	60.2	8 years	Any religious, educational, health-related or charitable volunteering in past year	

		Interview for Cognitive Status							
Y. Lee et al. (2021)	Interview, VI	- Happiness, see quotes<br><br>- Social connectedness<br><br>- Pain<br><br>- Self-Purpose	43	Community, United States and South Korea	Korea: 63.0 (Range 61 to 73 years)<br><br>US: 74.0 (Range 60 to 84 years)	Korea: 100.0<br><br>US: 87.5	6 months	Beautiful Story Grandma (Korea) and Foster Grandparents (US) programs	Volunteering "helps them (older adults) live a happy life."<br><br>Volunteering "makes them (older adults) active in society."<br><br>"I felt no pain at all. It was just amazing … It was almost like a drug."<br><br>"It (volunteering) gives me a purpose to get up and out of the house every day."
Lee et al. (2011)	Cohort, IV	- Mortality rate, National Death Index data	6 408, those who drive and those who do not drive	Community, United States	This paper also focused on driving status. Data are grouped by:<br><br>Volunteer Drivers: 75.0 (5.0)	Volunteer Drivers: 49.0<br><br>Volunteer Non-Drivers: 79.0<br><br>Non-Volunteer Drivers: 37.0	2 years	Any religious, educational, health-related or charitable volunteering in past year	

					Volunteer Non-Drivers: 78.0 (6.0)<br><br>Non-Volunteer Drivers: 75.0 (5.0)<br><br>Non-Volunteer Non-Drivers: 79.0 (7.0)	Non-Volunteer Non-Drivers: 69.0			
Lee (2019)	Cohort, IV	- Life satisfaction, Life Satisfaction Index	208	Community, United States	94.4 (7.6)	73.6	8 years	Volunteer work	
Lee (2023)	Cohort, IV	- Loneliness, single item with Likert scale responses<br><br>- Quality of Life, CASP-12	32 839	Community, 28 European countries	73.5 (6.6)	58.0	4 years	Voluntary or charity work	
Lee (2024)	Cross Sectional, IV	- Quality of Life, CASP-12 scale<br><br>- Life satisfaction, single item with Likert scale responses<br><br>- Self-rated health, single	37 239	Community, 27 European countries	74.9 (7.3)	55.0	2017	Any volunteer or charity work in the past year	

		item with Likert scale responses<br><br>- Depression, EURO-D scale<br><br>- Loneliness, UCLA-Loneliness scale							
Lee et al. (2025)	Cohort, IV	- Likelihood of subsequent exercise, Participants indicated the frequency with which they engaged in exercise in the past year	13 771	Community, United States	72.7 (10.4)	67.5	12 years	Any volunteer activity	
Lee & Kim (2014)	Cross Sectional, IV	- Depression, CES-D	3 968	Community, South Korea	74.5 (6.7)	57.1	2010	Volunteering groups	
Li & Ferraro (2005)	Cohort, IV	- Depression, CES-D	1 669	Community, United States	72.4 (8.2)	62.0	5 years	Volunteering in church, synagogue, or other religious organization, school or educational organization, political group or labor union, senior citizen group or any other national or local	

								organization in the past year	
Li et al. (2013)	Cohort, IV	- Depression, CESD-10<br><br>- Life satisfaction, ten items derived from the Life Satisfaction Index-A<br><br>- Health, single item with Likers scale responses<br><br>- Functional impairment, IADL scale	1 847	Community, Taiwan	65.9 (7.7)	48.1	8 years	Volunteer activity	
Lim et al. (2023)	Cohort, IV	- Depression, CES-D 8<br><br>- Friendship, measured via three further questions:<br>-Number of close friends<br>-Frequency of contact with friends (with Likert scale responses)<br>-Positive friendship quality (2 items with Likert scale responses)	4 532	Community, United States	68.0 (9.2)	60.7	8 years	Any religious, educational, health-related or charitable volunteering in past year	

Lim et al. (2025)	Cohort, IV	- Cognitive function, Langa-Weir Classification Scale	15 548	Community, United States	63.1 (10.0)	53.3	10 years	Charity work	
Ling et al. (2023)	Cross Sectional, IV	- Mental well-being, WHO-5	193	Community, China	Aged 51 to 60: 34.7% Aged 61+: 65.3%	77.0	1 month	Any volunteering	
Liu et al. (2020)	Cross Sectional, IV	- Depression,<br><br>- Physical health, single item with Likert scale responses<br><br>- Physical function, IADL scale<br><br>- Attitude towards aging, Attitudes to Aging Questionnaire	10 792	Community, China	70.7 (8.1)	52.1	2014	Volunteering via community patrols, caring for the elderly or children, environmental, escort chat, applying professional skills, and other volunteer activities	
Luhr et al. (2022)	Cohort, IV	- Life satisfaction, single item rated 0 to 10<br><br>- Loneliness, one item with Likert scale responses	3 736	Community, Germany	All 65 to 75 years old	Not stated	31 years	Political and Non-Political volunteering	
Lum & Lightfoot (2005)	Cohort, IV	- Depression, CES-D	7 322	Community and Care Homes, United States	77 (Not stated)	60.0	7 years	100+ hours of formal volunteering for any religious or	

		- Health, single item with Likert scale responses<br><br>- ADLs, 5 item scale based on ability to perform certain functional tasks<br><br>- IADLs, 5 item scale based on ability to perform certain functional tasks<br><br>- Mortality rate, measured as deceased, living in nursing home or living in community at follow-up						charitable organization in the past year	
Lyons et al. (2021)	Cross Sectional, IV	- Health, single item question with Likert scale responses rating their own health<br><br>- Social connectedness, Participants rated how connected they felt to the LGBTI community on a scale of 1 to 4	754, Lesbian and gay older adults	Community, Australia	All aged 60+ years, majority 60 to 64 years (43.5%)	32.2	4 months	Volunteering or giving unpaid help, in the form of time, service or skills, through an organization or group	

		- Social Support, 12-item Interpersonal Support Evaluation List<br><br>- Psychological wellbeing/ distress, K10 Scale<br><br>- Mental wellbeing, Short Warwick Edinburgh Mental Well-Being Scale							
Matsuda et al. (2024)	Interview, VI	- Anxiety<br><br>- Personal Growth	8	Community, Japan	55.1 (Range 69 to 78 years old)	87.5	2 months	Supporting 'frailty check-up' clinics (assisting with administration, advice, encouragement, simple measurement tasks)	"I feel very anxious. I wonder whether I will be able to perform this volunteer activity."<br><br>"I have a sense of anxiety about whether I will be able to keep pace…" (Harm)<br><br>"We don't have a place where we can become nervous. This is the only place I can dare put myself in such situations. I

		- Happiness							really appreciate it.”<br>“I think it is important to improve ourselves as (volunteers).”<br><br>“I was very happy when I heard it… It is nice to receive such compliments.”
Matthews & Nazroo (2021)	Cohort, IV	- Depression, CES-D 8<br><br>- Life satisfaction, Satisfaction with Life Scale<br><br>- Loneliness, UCLA Loneliness Scale<br><br>- QOL, CASP-19	3 740	Community, England	Volunteers:<br>Female: 71.4 (Not stated)<br>Male: 72.4 (Not stated)<br><br>Non-volunteers:<br>Female: 74.1 (Not stated)<br>Male: 74.5 (Not stated)	Volunteers: 59.4<br><br>Non-volunteers: 57.8	2 years	Formal volunteering by raising money, leading a group or committee, organizing activities or events, visiting, befriending or mentoring, teaching, counseling, secretarial work, providing transport, representing, campaigning, other practical help or other help within an organizational setting	Note: This analysis compared formal volunteers with informal volunteers. No analysis compared formal volunteer with non-volunteers. Demographic data presented Volunteers (combined formal and informal volunteers) and Non-Volunteers.

Mayers et al. (2024)	Cross Sectional, IV	- Depression, GDS-15<br>- Loneliness, UCLA LS3<br>- Frailty, KCL<br>- Sleep Quality, PSQI<br>- Dysphagia risk, DRACE<br>- Physical Activity, PASE	500, no diagnosis of dementia	Community, Japan	73.6 (8.9)	50.0	1 month	Any volunteering	
Mechakra-Tahiri et al. (2010)	Cross Sectional, IV	- Depression, ESA-Q (diagnostic tool developed by the research team)	2 670, no cognitive impairment	Community, Canada (Quebec)	All aged 65+ years, majority 65 to 69 years	59.8	2 years	Regular volunteer work	
Misener et al. (2010)	Interview, VI	- Happiness	20	Community, United States	72.0 (Not stated)	25.0	Not stated	Volunteering for local sports organizations	"I think I make a difference. I have confidence in what I do."<br><br>One participant felt upset after receiving criticism in their role, "Sometimes you get criticized for trying to help and that is the most frustrating part. You give your time and you get criticized." (Harm)

		- Physical activity							“I think you’ve got to keep busy. It’s so important as a person gets older. It’s great that I’m still volunteering. I think otherwise, you’d start feeling sorry for yourself, sitting at home doing nothing.”
		- Physical health							“Personally, my health has improved so much because of sports and volunteering and being active. I don’t take any prescriptions.”
		- Social connectedness							“We’ve built up real trust and loyalty within the group, and we’re there for each other in more ways than you can imagine.”
		- Attitude towards aging							“Because we are in an organization like this with so many seniors, we

									are going to lose some friends [who pass away].” (Harm)<br><br>“I like their enthusiasm, it makes me feel young.”
Moncayo-Hernandez et al. (2024)	Cross Sectional, IV	- Falls risk, ‘Have you fallen in the past year? Yes/ No’	17 687, majority of participants resided in cities with environmental barriers that may result in falls	Community, Colombia	69.3 (7.2)	56.2	2015	Volunteering groups in social welfare services, senior centers, children’s home centers, schools or universities, healthcare providers, churches or temples.	
Monserud (2025)	Cross Sectional, IV	- Cognitive functioning, CCCE<br><br>- Depression, CES-D	13 099	Community, Mexico	63.7 (9.6)	57.7	2018	Volunteering for a non-profit organization without pay or compensation	
Morrow-Howell (1999)	Cross Sectional, IV	- Physical health, single item question with Likert scale responses<br><br>- Social connectedness, single item question with	289	Community, United States	71.0 (6.7)	86.0	5 months	OASIS – Providing education to other older adults	

		Likert scale responses<br><br>- Personal growth (“learn more”), single item question with Likert scale responses							
Morrow-Howell et al. (2009)	Cross Sectional, IV	Life satisfaction, single question with Likert scale responses<br><br>- Social connectedness, two questions with Likert scale responses<br><br>- Personal growth, two questions with Likert scale responses<br><br>- Health, single question with Likert scale responses	401	Community, United States	All aged 51+ years, majority 71 to 80 years (41.4)	65.6	7 months	13 different volunteer programs that had a name, had a goal to improve specific human or environmental affairs and specifically recruited older adults, including tutoring, assisting with instrumental activities, mentoring, policing/public safety activities, counselling/technical advice, conservation activities, and supportive counselling	
Mukherjee (2010)	Interview, VI	- Happiness	9	Online, United States	61.0 (Range 55 to 64 years)	22.2	Not stated	Senior Net – online/ virtual volunteering	“It gives me great joy when I log in.”<br><br>One participant felt “at a loss”

		- Social connectedness							and “frustrated” when their emails went unanswered by the organization. (Harm)<br><br>“At my age, I don't have friends or children who I can chat or e-mail continually…as a volunteer, I feel there are people on the other side who are eagerly waiting for my e-mails.”
Musick et al. (1999)	Cohort, IV	- Mortality rate, National Death Index data, interviews	1 211	Community, United States	73.0 (Not stated)	60.0	8 years	Volunteering for any educational, religious, political, senior citizen or other national organization	
Musick & Wilson (2003)	Cohort, IV	- Depression, CESD-11	3 617	Community, United States	70.7 (Not stated)	67.0	8 years	Volunteering for any educational, religious, political, senior citizen or other national organization	
Myers et al. (2013)	Cross Sectional, IV	Self-Purpose, Single item question within Faith Maturity Scale	979, Protestant Christians	Community, United States	73.3 (5.9)	58.0	Not stated	Community ministry volunteering - involvement in activities	

		Faith development, Faith Maturity Scale						encouraged by your church that support the physical, material, emotional, and social well-being of people from your congregation, neighborhood, and community.	
Nakamura et al. (2023)	Cross Sectional, IV	- Epigenetic age, 13 DNAm epigenetic clocks	4 011	Community, United States	69.4 (9.6)	58.4	2016	Any religious, educational, health-related or charitable volunteering in past year	
Nakamura et al. (2025)	Cohort, IV	- Mortality risk, all-cause mortality	9 662	Community, United States	68.0 (9.0)	59.0	6 years	Any religious, educational, health-related or charitable volunteering in past year	
Newman (1983)	Mixed Methods, IV	- Life satisfaction, single item with Likert scale responses<br><br>- Physical health, single item with Likert scale responses<br><br>- Mental health, single item with Likert scale responses		Community, United States					"I feel I appreciate life a little more."

		- Social life, single item with Likert scale responses<br><br>- Energy levels, single item with Likert scale responses<br><br>- Self-Worth, single item with Likert scale responses<br><br>- Self-Purpose							“I look forward to Monday. In the morning, I walk to the bus station and I am humming to myself.”
		- Attitude towards aging							“I'm pushing 70...by being with children, I continue to feel young.”
		- Happiness							“I get a lot out of this work. It is really a joy to be with children.”
		- Pleasure							“I want to tell you that those children saved

									my life. Absolutely saved my life. I became so enthusiastic."
Newman et al. (1985)	Mixed Methods, IV	- Life satisfaction, single item with Likert scale responses<br><br>- Feelings about self, single item with Likert scale responses<br><br>- Mental health, single item with Likert scale responses<br><br>- Pleasure, see quotes<br><br>- Self-Purpose, see quotes	Questionnaire: 180<br><br>Interview: 60	Community, United States	64.4 (Range 55 to 85 years)	50.6	Not stated	Assisting students in classrooms	"I want to tell you that the love and support of those children saved my life. Absolutely saved my life."<br><br>"I feel we (volunteers) belong somewhere… It is a good feeling to know that children need me and that I am still good for

									something useful.”
Newman et al. (1995)	Cohort, IV	- Memory, Rivermead Behavioral Memory Test and Memory Functioning Questionnaire<br><br>- Depression, GDS	26	Community/ schools, United States	All aged 60+ years, majority 60 to 69 years (69.2%)	84.6	8 months	Assisting students aged 5 to 18 years with their learning in a school setting	
Nonaka et al. (2017)	Cohort, IV	- Functional Competence, TMIG-IC	1 320 total<br><br>‘Declined’ functional competence group: 306<br><br>‘Maintained’ functional competence group: 994	Community, Japan	‘Declined’ functional competence group: 74.6 (6.1)<br><br>‘Maintained’ functional competence group: 71.5 (4.9)	‘Declined’ functional competence group: 45.8<br><br>‘Maintained’ functional competence group: 53.9	4 years	Volunteering groups	
O’Reilly et al. (2017)	Cohort, IV	- Mortality rate, all cause mortality at follow-up	244 429	Community, Northern Ireland	All aged 65+ years, majority 65 to 69 years (41.9%)	53.6	4 years	Volunteering	
O’Shea (2006)	Mixed Methods, IV	- Life satisfaction, single item with Likert scale responses and see quotes	Questionnaire: 110, Senior Helpline volunteers	Community, Ireland	All aged , majority 70 to 80 years (50%)	Not stated, majority women		Senior Helpline – telephone listening service for older people by trained older volunteers	“...gives me a strong sense of satisfaction.”

		- Stress/ anxiety, see quotes<br><br>- Mental health, single item with Likert scale responses<br><br>- Compassion for others, see quotes<br><br>- Feelings about self, single item with Likert scale responses	Focus Groups: Not stated						Some calls were "difficult to forget about." Some participants "felt ill-equipped to take calls about sexual abuse." (Harm)<br><br>"…broadening my understanding of other people's lives."
Oman et al. (1999)	Cohort, IV	- Mortality rate, all-cause mortality via National Death Index, death notices and exit interviews	1 972	Community, United States	All aged 55+ years, majority 65+	58.0	6 years	Formal volunteer work	
Pardasani (2018)	Mixed Methods, IV	- Social connectedness<br><br>- Self-Purpose	Questionnaire: 172, senior center participants<br><br>Focus Groups: Specific data for focus groups not stated	Community, United States	All aged 60+ years, majority 60 to 69 years (43.0%)	58.1	Not stated	Volunteering at the senior center	"I made friends with the parents and the other volunteers."<br><br>"(Volunteering) gives me something to do… I don't want to let them down."

		- Compassion for others<br><br>- Pleasure							"The children need our help… We have to help them."<br><br>"We have so much fun."
Parisi et al. (2015)[a]	RCT, II	- Physical activity, 3 items including shopping, gardening, camping<br><br>- Intellectual activity, 7 items including reading a book, completing puzzles	Volunteering: 352<br><br>Control: 350	Elementary schools, United States	Volunteering: 67.4 (5.9)<br><br>Control: 67.4 (5.8)	Volunteering: 85.0<br><br>Control: 85.0	12 months	Volunteering: Experience Corps program – assisting elementary school children in the classroom<br><br>Control: Usual volunteer activity	
Parkinson et al. (2010)	Cohort, IV	- Physical health, SF-36<br><br>- Mental health, SF-36<br><br>- Social support, Duke Social Support Index	7 088, Women with over-sampling of people in remote areas	Community, Australia	All aged 70 to 75 years	100.0	9 years	Volunteering for any community or social organizations	
Pavlova & Silbereisen (2012)	Cross Sectional, IV	- Depression, 5 items from the Brief Symptom Inventory	1 422	Community, Germany	65.8 (5.9)	52.3	1 month	Any formal volunteering	

		- Life satisfaction, single item 1 to 7 scale<br><br>- Positive affect, Positive and Negative Affect Schedule							
Pettigrew et al. (2020)[b]	RCT, II	- Life satisfaction, single item with Likert scale responses<br><br>- Depression, CES-D<br><br>- Social connectedness, Social Provisions Scale<br><br>- Self-Esteem, Rosenberg Self-Esteem Scale<br><br>- Self-Purpose, Ryff's Psychological Wellbeing Scale<br><br>- Self-Efficacy, General Self-Efficacy Scale<br><br>- Personal growth, Ryff's Psychological Wellbeing Scale	Volunteering: 201<br><br>Control: 244	Community, Australia	Volunteering: 70.8 (5.7)<br><br>Control: 70.6 (6.1)<br><br>(Above are data at 6-months post-intervention)	Volunteering: 61.0<br><br>Control: 49.0<br><br>(Above are data at 6-months post-intervention)	6 months	Volunteering: Any formal volunteering of participants' choice, minimum 1hour per week<br><br>Control: Not advised to volunteer, not required to refrain from volunteering	

		- QOL, Global QOL scale<br><br>- Psychological wellbeing, Warwick-Edinburgh Mental Wellbeing Scale<br><br>- Number of chronic health conditions, single item reporting number<br><br>- Body weight/ obesity, Body Mass Index, weight circumference<br><br>- Walking speed, 6m walk test – normal and fast pace<br><br>- Dynamic balance, 6m backwards walking test<br><br>- Walking endurance, time taken to walk 400m at a fast pace							

		- Lower limb strength, 5 x sit to stand test<br><br>- Strength, 1 repetition maximum chest press, leg extension, seated row<br><br>- Physical activity, step count via accelerometer<br><br>- Physical fitness, Resting heart rate							
Proulx et al. (2018)	Cohort, IV	- Cognitive function, Sum score of a 10-word immediate and delayed recall test of memory, a serial 7s subtraction test of working memory and counting backwards	11 100	Community, United States	66.4 (10.1)	53.4	16 years	Any religious, educational, health-related or charitable volunteering in past year	
Resnick et al. (2013)	Cross Sectional, IV	- Depression, Patient Health Questionnaire-2<br><br>- Physical activity, Yale Physical Activity Survey	129, residents in a continuing care retirement community	Independent or assisted living in a continuing care retirement community, United States	87.7 (6.5)	Not stated	Not stated	Range of volunteer activities within or external to the care community	

		- Pain, Numerical Rating Scale<br><br>- Fear of Falling, rated 0 to 4<br><br>- Physical resilience, Physical Resilience Scale							
Rogers et al. (2016)	Cohort, IV	- Mortality rate, mortality data from National Health Service records<br><br>- Physical function, proportion of people requiring assistance with items from ADL and IADL scales	10 324<br><br>Volunteers: 1 957<br><br>Non-volunteers: 8 367	Community, England	Volunteers: 64.8 (9.3)<br><br>Non-volunteers: 65.1 (10.5)	Volunteers: 57.6<br><br>Non-volunteers: 53.6	11 years	Volunteering at least once per month	
Rook & Sorkin (2003)	Case Control, IV	- Self-Esteem, Rosenberg Self-Esteem Scale<br><br>- Loneliness, UCLA Loneliness Scale<br><br>- Depression, CES-D 20<br><br>- Social connectedness,	- Foster grandparent program for children with developmental disabilities: 52<br><br>- Peer befriending: 69	Community, United States	- Foster grandparent program for children with developmental disabilities: 69.6 (6.7)<br><br>- Peer befriending: 68.9 (6.3)	- Foster grandparent program for children with developmental disabilities: 67.3<br><br>- Peer befriending: 69.6	3 years	- Foster grandparent program for children with developmental disabilities<br><br>- Peer befriending<br><br>- Community sample	

		series of name-eliciting questions and associated relationships and experiences with that name	- Community sample: 59		- Community sample: 73.2 (7.0)	- Community sample: 59.3			
Ryu & Heo (2018)	Cross Sectional, IV	- Life satisfaction, Satisfaction with Life Scale<br><br>- Health, single item with Likert scale responses	188, senior center attendees	Community, South Korea	75.0 (5.5)	64.3	Not stated	Volunteer activities	
Sabin (1993)	Cohort, IV	- Mortality rate, alive or deceased at follow up and National Death Index data	7 502	Community, United States	Not stated	Not stated	4 years	Volunteering in the past 12 months	
Sanchez-Garcia et al. (2025)	Cohort, IV	- Happiness, Self-rated health, Likert scale<br><br>- Life Satisfaction, Self-rated health, Likert scale<br><br>- Self-rated health, single item with Likert scale responses	8 331	Community, 29 European countries	All 70+ years	59.0	2 years	Any volunteering in the past month	
Seeman et al. (2020)	Cohort, IV	- Conserved transcriptional response to	18	Elementary schools, United States	68. 0 (Range 60.1 to 80.7)	89.0	9 months	Classroom assistance to third grade students	

		adversity (CTRA) gene expression profiles (involved in inflammation and anti-viral responses), blood test							
Sellon (2018)	Interview, VI	- Social connectedness<br><br><br><br><br><br><br><br><br><br><br><br><br><br><br><br><br><br><br>- Physical activity<br>- Physical function	20, people with mobility impairment (serious difficulty walking or climbing stairs)	Community, United States	66.0 (Range 55.0 – 80.0)	70.0	8 months	Formal volunteering	"Well it is really great for networking. You get to know people in different parts of the city, county, state, whatever."<br><br>"(Volunteering) keeps you out in the community. It keeps you interesting and interested and if you don't do that, unless you are inclined to be a hermit."<br><br>"Volunteering has actually been my OT/PT. I have learned how to write better. I have learned how to dribble a basketball. I have learned how to shoot

		- Pain<br><br>- Self-Purpose							basketballs. Who do you think taught me all those things? The children."<br><br>"I find that if you stay active you don't pay attention to your aches and pains."<br><br>"(Volunteering) is where I get a lot of my satisfaction, purpose in life."
Sellon (2023)	Interview, VI	- Compassion for others<br><br>- Stress/ Anxiety<br><br>- Physical health	20, people with mobility impairment (serious difficulty walking or climbing stairs)	Community, United States	66.0 (Range 55.0 – 80.0)	70.0	8 months	Formal volunteering	"I want to help other people. I thought (volunteering) would be a good place to help other people,"<br><br>"So in my mind, it is just all good and (volunteering) overrides (the bad)."<br><br>"(Volunteering) keeps me engaged. It keeps me active."

		- Social connectedness<br><br>- Self-Purpose							“Volunteering is my main social outlet”<br><br>“If you don't have a reason to get up in the morning it is pretty easy to lay around and do nothing whereas I have this (volunteering) to do and this is expected.”
Serrat-Graboleda et al. (2021)	Mixed Methods, IV	- Health, Personal Wellbeing Index<br><br>- Attitude towards aging, see quotes<br><br>- Life satisfaction, Personal Wellbeing Index<br><br>- Happiness, see quotes<br><br>- Self-Purpose, see quotes	Survey: 85<br><br>Focus groups: 21	Community, Spain	71.2 (7.5)	Not stated	Not stated	Volunteering activities	“Extends the life of those who do it.”<br><br>Participants responded that volunteers were generally happy. Some participants reported “sadness over having to see the needs of others”

		- Satisfaction with interpersonal relationships, Personal Wellbeing Index							and that "seeing ill people can be depressing." (Harm)<br><br>"Feeling useful."
Shimanuki et al. (2007)	Case Control, IV	- Frequency of social interaction, single item with Likert scale responses<br><br>- Health, single item with Likert scale responses<br><br>- Self-Efficacy with ADLs, Japan Institute of Gerontology's Activity Ability Index<br><br>- Intellectual activity, Japan Institute of Gerontology's Activity Ability Index<br><br>- IADLs, Japan Institute of Gerontology's Activity Ability Index	Volunteers: 69<br><br>Control: 1 207	Community, Japan	Volunteers: 73.0 (2.6)<br><br>Control: 75.5 (4.0)	Volunteers: 26.1<br><br>Control: 63.1	1 year	Care Prevention Volunteering e.g. providing falls prevention classes, health lectures	

		- Physical fitness, Japan Institute of Gerontology’s Activity Ability Index<br><br>- Daily physical activity, QOL scale<br><br>- Social role, Japan Institute of Gerontology’s Activity Ability Index<br><br>- Mental health, QOL scale<br><br>- Mental vitality, QOL scale<br><br>- Health satisfaction, QOL scale							
Shmotkin et al. (2003)	Cross Sectional, IV	- Physical activity, mean frequency of rigorous activity<br><br>- Health, single item with Likert scale responses<br><br>- Chronic diseases incidence, self-reported	148, Jewish people	Community, Israel	82.1 (4.8)	45.3	1989	Formal volunteering led by an organization, for example, charity organizations or hospitals	

		- Cognitive functioning, Mini Mental State Exam<br><br>- Depression, CES-D<br><br>- Number of close relationships (social connectedness), self-reported<br><br>- Mortality, all cause mortality							
Sneed & Cohen (2013)	Cohort, IV	- Hypertension risk, blood pressure readings at baseline and follow-up<br><br>- Psychological wellbeing, nine psychological variables combined	1 381, not hypertensive at baseline	Community, United States	64.2 (8.9)	59.7	4 years	Any religious, educational, health-related or charitable volunteering in past year	
Stathi et al. (2021)	Interview, VI	- Social connectedness<br><br>- Self-Purpose	13	Community, England	66.8 (4.3)	77.0	6 months	Active, Connected, Engaged program – peer volunteering to support active aging in other older adults	"It's nice having a new social circle; I've got to meet other people I wouldn't have come across"

									"It gives people a purpose…you know, you're expected somewhere and wanted somewhere."
Stewart et al. (2024)	Interview, VI	- Social connectedness<br><br><br><br><br><br><br><br><br><br><br><br>- Pleasure<br><br><br><br><br><br><br><br>- Self-Purpose	17	Community, Canada	Range 65 to 87 years	76.5	Not stated	Any formal volunteering	"It got me in touch with a lot of people [in the community] … I feel an involvement."<br>"It's just another way of connecting to the community that I think is important."<br><br>"[Volunteering] gives me a lot of pleasure."<br>"As soon as I retired, I started [volunteering] and I really enjoyed that."<br><br>"I had to find things to fill my time… to be helpful and feel like I'm doing something good."

		- Energy levels							“It's a lot of work for volunteers. You're there for six or seven hours, which is a large commitment of time in the evening. It's sometimes 11:30 [pm] before we finish. And it's quite hard work, actually.” (Harm)<br><br>“I'm just getting [more] tired … pulling myself together at 8:30 in the evening and dragging myself out, it's getting to be harder work than it used to be.” (Harm)<br><br>“I'm trying to wean [my volunteer organisation] from me, from the expectation…” (Harm)
Sun et al. (2025)	Mixed Methods, IV	- Total benefits, total benefits sum	319	Within schools during	All aged 50+ years,	66.0	Not stated	Tutoring school children	

		score derived by the authors including a range of health measures<br><br>-Social connectedness<br><br>- Pleasure<br><br>-Self-Purpose		COVID-19, United States	majority aged 70-79 (46.8%)				"Tutoring this year kept me connected to people."<br><br>"It gives me joy."<br><br>"Gave me a purpose and a reason to keep moving… I feel like I'm doing something very worthwhile."
Sung et al. (2023)	Cohort, IV	- Lifelong learning, dichotomous item whether participants had attended any education or training course in the past year	2 608	Community, Singapore	70.9 (7.9)	53.3	3 years	Unpaid help through organizations, groups or clubs in the past year	
Tamura et al. (2021)	Cohort, IV	Depression, GDS-15	37 552	Community, Japan	All over 65 years, majority over 85 (18.4%)	53.1	3 years	Volunteering more than once per month	

Tamura et al. (2024)	Cohort, IV	Survey responses over time in relation to:<br><br>- Physical/ cognitive health (e.g. onset of functional disability, dementia diagnosis)<br><br>- Health behaviors (e.g. sedentary lifestyle, smoking)<br><br>- Mental health (e.g. depressive symptoms, loneliness)<br><br>- Subjective wellbeing (e.g. happiness, loneliness)<br><br>- Social wel-being (e.g. number of friends seen per month)<br><br>- Cognitive social capital (e.g. community attachment)	34 187, physically and cognitively independent older adults	Community, Japan	72 (4.9)	48.2	6 years	Any volunteer group participation	

Tan et al. (2006)[a]	RCT, II	- Physical activity, Minnesota Leisure Time Physical Activity Questionnaire, 2 items from Paffenbarger Physical Activity Questionnaire (number of blocks/ iles walked in past week, number of flights of stairs walked in past week), overall perceived activity asked in single item question	Volunteering: 59<br><br>Control: 54	Elementary schools, United States	All aged 59+ years<br><br>Volunteering: Majority 71 to 75 years (37.0%)<br><br>Control: Majority 66 to 70 years (46.0%)	Volunteering: 92.0<br><br>Control: 96.0	4 to 8 months	Volunteering: Experience Corps program – assisting elementary school children in the classroom<br><br>Control: Placed on waiting list and did not volunteer	
Tan et al. (2009)	Cohort, IV	- Physical activity, Minnesota Leisure Time Physical Activity Questionnaire	71, African American women	Elementary schools, United States	71.2 (4.3)	100.0	3 years	Experience Corps program – assisting elementary school children in the classroom	
Tang (2009)	Cohort, IV	- Health, single item with Likert scale responses<br><br>- Physical function, six items with Likert scale responses<br><br>- Number of chronic	1 669	Community, United States	70.1 (7.4)	67.1	8 years	Volunteer work for a church, synagogue, or other religious organization; school or educational organization; political group or labor union; senior citizen group or	

		conditions, several questions asked respondents whether they had experienced health problems in the past year, including arthritis, lung disease, hypertension, heart attack, diabetes, cancer, foot problems, stroke, broken bones, and urine beyond control						related organi-zation; and any other national or local organizations, including United Fund, hospitals	
Taylor et al. (2024)	Cohort, IV	- Mortality data	16 939, not receiving veterans' support, Non-Indigenous	Community, Australia	Aged 65+ years	Not stated	4 years, participants drawn from a database (Register of Senior Australians 1997-2017), but participants are included from 2013 onwards	Any volunteering	
Tiittanen & Turjamaa (2022)	Focus Group, VI	- Social connectedness	38, rural population	Community, rural, Finland	76.8, Range: 65 to 81 years	73.7	1 year	Volunteer work e.g. Finnish Red Cross, Finnish Association for the Welfare of Older People	Volunteers reported socializing outside of their volunteering hours, "We have visited the

									theatre and we have been eating together.”
Tomioka et al. (2016)	Cross Sectional, IV	- IADL independence, TMIG-IC IADL subscale	14 956, independent in their basic ADLs on Barthel Index	Community, Japan	All aged 65+ years	53.6	2 months	Volunteer groups, frequency also captured	
Torres et al. (2023)	Cohort, IV	- Loneliness, single item question regarding frequency of loneliness	31 667	Community, 27 European countries and Israel, before and during COVID-19 (2019 to 2021)	67.8 (6.9)	46.9	2 years	Any volunteer work in the last 3 to 12 months	
Varma et al. (2015)	Focus Group, VI	- Stress/ anxiety<br><br>- Pleasure	46	Elementary schools, United States	66.7 (5.8)	84.8	Not stated	Experience Corps program – assisting elementary school children in the classroom	“What’s most challenging for me was that most of the students in my class had some sort of behavior problems. It was very stressful for me because some days we never got to a lesson.” (Harm)<br><br>“I enjoyed it.”<br><br>“Too many of our children are surrounded by negativity…they'

		 <br><br><br><br><br><br><br><br><br><br>- Self-Purpose<br><br><br><br><br><br><br><br><br><br>- Social connectedness							re raising siblings ...and they're six or seven. your heart goes out to them . . . Sometimes they will make you want to cry.” (Harm)<br><br>“When I left at the end of the day I felt like I had accomplished . . . a lot and given back by serving and helping these children.”<br><br>“Everyone was nice... they were like my family, or my friends.”<br><br>“They (the students) showed me how much they really did care for me, and how much they loved me . . . and I was beginning to feel the same way. Just like they were my grandchildren.”

		- Energy levels<br><br>- Physical function<br><br>- Fear of harm<br><br>- Happiness							“I’ve noticed that you feel more lively . . . [with] those four years-olds there’s no time for laid back.”<br><br>“I used to have to hold on the rail to go up the steps and come down the steps. Now I can walk up the steps and walk right down the steps.”<br><br>“I know I’m going to have to break up some fights . . . these are not little love taps, they’re serious.” – Regarding having to break up physical fights between students. (Harm)<br><br>“‘Hi Ms. X!’All up and down the street and I would meet them in stores . . . ‘There’s Ms. X!’ I know it made me feel happy.”

		- Psychological wellbeing							“To help others helps me. Because some days when I might . . . just have a . . . down feeling or a feeling of not being . . . just dead . . . One little child would need my help.”
Varma et al. (2016)	Cohort, IV	- Physical activity, steps per day on accelerometer	114	Elementary schools, United States	67.4 (6.0)	68.4	2 years	Experience Corps program – assisting elementary school children in the classroom	
Voloshina et al. (2023)	Cross Sectional, IV	- Self-purpose, Life Orientation Test<br>- Life Satisfaction, Life Orientation Test<br>- Anxiety, Integrated Anxiety Test<br>- Resilience, Vitality Test	40	Community, Russia	All aged 61+ years, majority 71 to 75 years	87.5	Not stated	Any volunteer work throughout the year	
Wang et al. (2021)	Cross Sectional, IV	- Depression, CESD-10	8 255, no Alzheimer’s or psychiatric diagnoses	Community, China	67.9 (6.4)	49.6	2015	Formal volunteering, such as participating in formal organizations or social group	

								activities with non-paying time and energy	
Warburton & Peel (2008)	Case Control, IV	- Hip fracture risk, hip fracture incidence, dichotomous	387 total<br><br>Cases: 126<br>Controls: 261	Community and Hospital, Australia	Cases: 82.5 (6.9)<br><br>Controls: 82.7 (6.8)	Cases: 81.7<br><br>Controls: 82.0	Not stated	Voluntary unpaid work, e.g. community service	
Warburton & Winterton (2017)	Interview, VI	- Social connectedness, see quotes<br><br>- Stress/ anxiety, see quotes<br><br>- Physical activity, see quotes	60	Rural community, Australia	60 (Not stated)	51.6	Not stated	Volunteering within local rural community, for example, Red Cross, Historical Society	"Recluses…come along and they hardly talk to anybody, but they're still good friends."<br><br>"(volunteering is) good therapy. It takes my mind off my worries…"<br><br>"I am busy but sometimes you can be too busy, which is not good either." (Harm)<br><br>"I keep myself…active all day long (due to volunteering)."
Warburton et al. (2001)	Cross Sectional, IV	Participants were asked to rate how likely volunteering	238	Community, Australia	All aged 65 to 74 years	52.5	1996	Any service to the community given without payment through any group	

		would result in them experiencing a series of costs and benefits if they were to volunteer in the next month, on a 7-item scale.<br><br>Items included:<br><br>- Life satisfaction<br><br>- Pleasure<br><br>- Social connectedness ("meet people")<br><br>- Self-Purpose ("feel useful")						or organization, including welfare groups, local community organizations, the church, as well as sporting or social groups.	
Ward (2024)	Cross Sectional, IV	- Wish to die, single item	6 915	Community, Ireland	62.8 (95% CI 61.1 to 64.4)	52.1 (95% CI 51.1 to 53.1)	Approximately 2 years	Membership of voluntary organisations	
Warner et al. (2024)[c]	RCT, II	- Loneliness, UCLA-20 (Chinese), DeJong Gierveld Loneliness Scale<br><br>- Engagement with social network, Lubben Social Network Scale	Volunteering: 185<br><br>Control: 190<br><br>Currently feeling lonely: UCLA-3 Loneliness scale >5 (indicating higher	Community, Hong Kong	Volunteering: 63.0<br><br>Control: 64.0	Volunteering: 71.4<br><br>Control: 83.7	6 months	Active volunteering: Training and then delivering telephone-based behavioral activation, mindfulness and befriending interventions to participants in the concurrent trial, 60 to 120 mins per	

		- Perceived social support, Multidimensional Scale of Perceived Social Support<br><br>- Stress, Perceived Stress Scale<br><br>- Symptoms of depression, Patient Health Questionnaire<br><br>Symptoms of Anxiety, Hospital Anxiety and Depression Scale	loneliness levels).<br>No severe mental, physical or cognitive impairment, UCLA-3 Loneliness scale >5 (indicating higher loneliness levels)					week/ 2 to 4 sessions per week.<br><br>Control: Attending a psychoeducation program with social gatherings, 6 x 2-hour sessions.	
Webster et al. (2021)	Cohort, IV	- Depression, CES-D 20<br><br>- Physical health, single item with Likert scale responses	556	Community, United States	67.0 (11.8)	60.1	13 years	Any volunteering	
Weziak-Bialowolska et al. (2024)	Cohort, IV	- Emotional wellbeing, CASP-12<br><br>- Loneliness, UCLA-3<br><br>- ADL and IADL ability, Authors 0 to 6 and 0 to 7	19 821	Community, 15 European countries	All aged 50+ years, majority 60 to 69 years (40.1%)	59.3	6 years	Any voluntary or charity work in the past year	

		scores respectively, with a lower score indicating greater ADL independence<br><br>- Physical health, incidence of self-reported heart attack, hypertension, high blood cholesterol, stroke, diabetes, chronic lung disease, and cancer, pain or at least one mobility limitation. Or exit interviews with cause of death.<br><br>- Cognitive impairment, time orientation<br><br>- Alzhiemer’s disease/ other dementia, self-reported incidence<br><br>- Depression, EURO-D							
White et al. (2025)	Interview, VI	- Loneliness, see quotes	15, Meals on Wheels volunteers.	Community, Australia	Aged 67+ years	66.7	Not stated	Meals on Wheels (meal deliveries,	“If you are active and alone there are so many

		- Social Connectedness, see quotes	Additional participants who were volunteers at a Research Institute were not included in this review.					often to frail elderly)	things you can do (to feel less lonely). I mean there are all the charity, volunteer things you can join." "If you make an effort and you start meeting people - I have made great friends."
Windsor et al. (2008)	Cohort, IV	- Positive and negative affect, Positive and Negative Affect Schedule scales - Life satisfaction, Satisfaction with Life Scale	2 136	Community, Australia	Aged 64 to 68 years	Not stated	6 years	Volunteer work	
Withall et al. (2018)	Interview, VI	- Social connectedness - Self-Purpose	28	Community, England	70.8 (Range: 65 to 74 years)	66.7	3 months	Project Active, Connected, Engaged – peer volunteering to encourage active aging in other older adults	"If you're volunteering you meet people, make friends with people." "I'd just taken early retirement so... I was looking for something to do…. I loved being busy every day."

		- Pleasure							“That makes me feel really good, I’ve gone something good today. I made an old man happy. I look forward to the next day now.”
Wu et al. (2005)	Cross Sectional, IV	- Psychological distress, GHQ (12-item)<br><br>- Life satisfaction, Satisfaction with Life Scale (5-item)<br><br>- Self-Efficacy, Generalized Self-Efficacy Scale (10-item)<br><br>- Physical health, single item question with Likert scale responses	501	Community, Hong Kong	72.0 (7.5)	78.6	Not stated	Voluntary work	
Xi et al. (2025)	Cohort, IV	- Depression, PROMIS Short Form v1.0 Depression 4a instrument	2 990, participants in an aging drivers study	Community, United States	Aged 65+ years, majority 65 to 69 years (38.0%)	47.6	20 months	Any volunteering	

Xie & Han (2024)	Cross Sectional, IV	- Life Satisfaction, single item with Likert scale responses	11 418	Urban and rural environments, China	71.4 (7.3)	49.5	3 months	Volunteering requiring professional services, or involving security patrolling or environmental protection. Through a structured organization.	
Yamazaki et al. (2021)	Cohort, IV	- Physical function disability, Long-Term Care Insurance Certification (rates level of caregiving support required)	826	Community, Japan	75.2 (4.5)	52.0	14 years	Any formal volunteering	
Yang (2020)	Cohort, IV	- Depression, CESD-8	7 107, unemployed	Community, United States	Not stated	Not stated	12 years	Any religious, educational, health-related or charitable volunteering in past year	
Yasunaga et al. (2016)	Quasi-Experimental, III	- Health, demographics, hospitalizations, medications<br><br>- Social connectedness, frequency of going out, interactions with friends and	Volunteers: 67<br><br>Control: 74	Childcare centers, kindergartens and elementary schools, Japan	Volunteers: 68.2 (6.0)<br><br>Control: 68.7 (4.8)	Volunteers: 77.6<br><br>Control: 68.9	9 months	Volunteers: Playing hand games and reading to children at childcare centers kindergartens and elementary schools<br><br>Control: Conventional	

		neighbourhood children<br><br>- Psychological wellbeing, depression, self-esteem<br><br>- Physical function, IADLs, walking speed, functional walk test, one-leg stand time, functional reach test, intellectual activity, social function						social activities with no training or attendance at the above program	
Yeung (2025)[c]	RCT, II	- Loneliness, UCLA-20 (Chinese), DeJong Gierveld Loneliness Scale<br><br>- Engagement with social network, Lubben Social Network Scale<br><br>- Perceived social support, Multidimensional Scale of Perceived Social Support	Volunteering: 148<br><br>Control: 170<br><br>Volunteering: 131<br><br>Control: 168<br><br>Currently feeling lonely: UCLA-3 Loneliness scale >5	Community, Hong Kong	Volunteering: 63.0 (4.9)<br><br>Control: 64.0 (4.8)	Volunteering: 71.0<br><br>Control: 84.0	6 months<br><br>12 months	Active volunteering: Training and then delivering telephone-based behavioral activation, mindfulness and befriending interventions to participants in the concurrent trial, 60 to 120 mins per week/ 2 to 4 sessions per week.<br><br>Control: Attending a psychoeducation program with social gatherings,	

		- Stress, Perceived Stress Scale<br><br>- Symptoms of depression, Patient Health Questionnaire<br><br>Symptoms of Anxiety, Hospital Anxiety and Depression Scale<br><br>- Sleep quality, Sleep Condition Indicator<br><br>- Life satisfaction, Satisfaction with life scale<br><br>- Psychological wellbeing, Psychological wellbeing scale	(indicating higher loneliness levels).<br>No severe mental, physical or cognitive impairment, UCLA-3 Loneliness scale >5 (indicating higher loneliness levels)					6 x 2-hour sessions.	
Yuen et al. (2008)	RCT, II	- Depression, GDS<br><br>- Life satisfaction, Life Satisfaction Index-A<br><br>- Physical health, single item with Likert scale responses	Volunteering: 15<br><br>Control: 12<br><br>All living in residential care	Long-Term Residential Care Facilities, United States	Volunteering: 83.0 (9.9)<br><br>Control: 83.9 (7.7)	Volunteering: 80.0<br><br>Control: 66.7	3 months	Volunteering: Tutoring conversational English to English-as-an-Additional-Language students, 1 hour, twice per week	

								Control: Usual care facility lifestyle activities	

[a] Papers report on the same study by Fried et al. (2004)

[b] Papers report on the same study by Pettigrew et al. (2020)

[c] Papers report on the same study by Warner et al. (2024)

ADL = Activities of Daily Living

CASP-12 = Control, Autonomy, Self-Realization, Pleasure, 12 item scale

CCCE = Cross Cultural Cognitive Examination

CERAD = Consortium to Establish a Registry for Alzheimer's Disease

CES-D = Centre for Epidemiological Studies Depression Scale

CLASS = Chinese Longitudinal Social Survey

CRP = C-Reactive Protein, a measure of systemic inflammation

DRACE = Dysphagia Risk Assessment for Community-Dwelling Elderly

EURO-D = European Depression Scale

EQ-5D = Euro-Qol 5 Dimension

GDS-15 = Geriatric Depression Scale 15

GHQ = General Health Questionnaire

HAVEN = Healthy Ageing/ Vulnerable Environment

IADL = Instrumental Activities of Daily Living

KCL = Kihon Checklist

LOT-R = Life Orientation Test-Revised

LSS = Life Satisfaction Scale

MoCA = Montreal Cognitive Assessment

PASE = Physical Activity Scale for the Elderly

PROMIS = Patient-Reported Outcomes Measurement Information System

PSQI = Pittsburgh Sleep Quality Index

RCT = Randomized Controlled Trial

SENAS = Spanish and English Neuropsychological Assessment Scales

TICS = Telephone Interview for Cognitive Status

TICS-m = Modified Telephone Interview for Cognitive Status

TMIG-IC = Tokyo Metropolitan Gerontology Index of Competence

UCLA LS3 = University of California, Los Angeles Loneliness Scale 3

WHO-5 = World Health Organization Five Well-being index

Supplementary Material 3 – Risk of Bias Assessment Tables using CASP Checklists by paper design

RCTs (Yes (Y) / No (N) / Can't Tell (CT)):

Author (Year)	Did the study address a clearly formulated research question?	Was the assignment of participants to interventions randomised?	Were all participants who entered the study accounted for at its conclusion?	Were participants 'blind' to intervention they were given?	Were investigators 'blind' to the intervention they were giving to participants?	Were the people assessing/ analysing outcome/s 'blinded'?	Were the study groups similar at the start of the randomised controlled trial?	Apart from the experimental intervention, did each group receive the same level of care (that is, were they treated equally)?	Were the results of intervention reported comprehensively?	Was the precision of the estimate of the intervention reported?	Do the benefits of the intervention outweigh the harms and costs?	Can the results be applied to your local population/ in your context?	Would the experimental intervention provide greater value to the people in your care than any of the existing interventions?	Overall
Brydges et al. (2021)[a]	Y	Y	N	Y	CT	CT	Y	Y	Y	Y	Y	Y	Y	High risk
Carlson et al. (2015)[a]	Y	Y	N	N	CT	Y	Y	Y	Y	Y	Y	Y	Y	High risk
Fried et al. (2004)[a]	Y	Y	Y	N	CT	CT	N	Y	Y	Y	Y	Y	Y	High risk
George & Singer (2011)	Y	Y	Y	N	N	CT	Y	Y	Y	Y	Y	Y	Y	High risk
Jongenelis,	Y	Y	N	Y	N	CT	CT	Y	Y	Y	Y	Y	Y	High risk

Jackson, Newton et al. (2022)[b]														
Parisi et al. (2015)[a]	Y	Y	N	N	N	CT	Y	Y	Y	Y	Y	Y	Y	High risk
Pettigrew et al. (2020)[b]	Y	Y	N	Y	Y	CT	N	Y	Y	N	Y	Y	Y	High risk
Tan et al. (2006)[a]	Y	Y	Y	N	CT	Y	N	Y	N	N	Y	Y	Y	High risk
Warner et al. (2024)[c]	Y	CT – randomization was mentioned, however the process for randomization was unclear	Y - Loss to follow up total 15.2%, similar in both groups, authors include attrition analyses in Supplementary Materials	N – although this is difficult with the volunteering intervention	N	Y – blinded research assistants completed baseline and follow up assessments	N – differences in gender, age, illnesses and anxiety – important variable in this study	Y	N – not all results for both groups and not all direct comparisons at T2 are provided. Some results report significant interaction effects prior to or without clearly reporting between-group comparisons at T2	N – not for all between-comparisons at T2	Y	Y	Y	High risk

Yeung (2025)[c]	Y	Y	Y - Loss to follow up total 15.2%, similar in both groups, authors include attrition analyses in Supplementary Materials	N – although this is difficult with the volunteering intervention	N	Y – blinded research assistants completed baseline and follow up assessments	N – differences in gender, illnesses and anxiety – important variable in this study	Y	N – Some results report significant interaction effects prior to or without clearly reporting between-group comparisons at T3	Y	Y	Y	Y	High risk - Control group had a higher baseline level of anxiety, which may contribute to the significant decrease in anxiety scores in the control group compared to the volunteering group.<br><br>This paper includes results from Warner, above.
Yuen et al. (2008)	Y	Y	Y	N	N	N	Y	Y	Y	Y	Y	Y	Y	High risk

[a] Papers report on the same study by Fried et al. (2004)

[b] Papers report on the same study by Pettigrew et al. (2020)

[c] Papers report on the same study by Warner et al. (2024)

Quasi-Experimental (Yes (Y) / No (N) / Can't Tell (CT)):

Author (Year)	Was there a clear statement of the aims of the research?	Is quasi-experimental methodology appropriate?	Was the research design appropriate to address the aims of the research?	Was the recruitment strategy appropriate to the aims of the research?	Was the data collected in a way that addressed the research issue?	Has the relationship between the researcher and participants been adequately considered?	Have ethical considerations been taken into consideration?	Was the data analysis sufficiently rigorous?	Is there a clear statement of findings?	Can the results be applied to the local population?	Overall
De Souza et al. (2011)	Y	Y	Y	Y	Y	CT	CT	N	Y	Y	Medium risk
Hong & Morrow-Howell (2010)	Y	Y	Y	Y	Y	CT	CT	N	Y	Y	Medium risk
Hsiao et al. (2020)	Y	Y	Y	Y	Y	CT	CT	Y	Y	Y	Low risk
Hunter & Linn (1981)	Y	Y	Y	Y	Y	CT	CT	Y	Y	Y	Low risk
Yasunaga et al. (2016)	Y	Y	Y	Y	Y	CT	CT	N	N	CT	High risk

Mixed Methods (Yes (Y) / No (N) / Can't Tell (CT)):

Author (Year)	Was there a clear statement of the aims of the research?	Is mixed methods methodology appropriate?	Was the research design appropriate to address the aims of the research?	Was the recruitment strategy appropriate to the aims of the research?	Was the data collected in a way that addressed the research issue?	Has the relationship between the researcher and participants been adequately considered?	Have ethical considerations been taken into consideration?	Was the data analysis sufficiently rigorous?	Is there a clear statement of findings?	Can the results be applied to the local population?	Overall
Brown et al. (2009)	Y	Y	Y	Y	Y	CT	CT	Y	Y	Y	Medium risk
Fraser et al. (2009)	Y	Y	Y	Y	Y	CT	CT	N	Y	Y	Medium risk
Gagliardi et al. (2020)	Y	Y	Y	Y	Y	CT	CT	N	Y	Y	High risk
Larkin et al. (2005)	Y	Y	Y	Y	Y	CT	CT	N	Y	Y	High risk
Newman (1983)	Y	Y	Y	Y	Y	CT	CT	N	Y	Y	High risk
Newman et al. (1985)	Y	Y	Y	Y	Y	CT	CT	N	Y	Y	High risk
O'Shea (2006)	Y	Y	Y	Y	Y	CT	CT	N	Y	Y	High risk
Pardasani (2018)	Y	Y	Y	Y	Y	CT	CT	Y	Y	Y	Low risk
Serrat-Graboleda et al. (2021)	Y	Y	Y	Y	Y	Y	Y	Y	Y	Y	Low risk
Sun et al. (2025)	Y	Y	Y	Y	Y	CT	CT	Y	Y	Y	Low risk

Cohort (Yes (Y) / No (N) / Can't Tell (CT)):

Author (Year)	Did the study address a clearly focused issue?	Was the cohort recruited in an acceptable way?	Was the exposure accurately measured to minimise bias?	Was the outcome accurately measured to minimise bias?	Have the authors identified all important confounding factors?	Have they taken account of the confounding factors in the design and/or analysis?	Was the follow up of subjects complete enough?	Was the follow up of subjects long enough?	Are the results precise?	Do you believe the results?	Can the results be applied to the local population?	Do the results of this study fit with other available evidence?	Overall
Abe et al. (2022)	Y	Y	Y	Y	Y	Y	Y	Y	Y	Y	Y	Y	Low risk
Abe et al. (2023)	Y	Y	Y	Y	Y	Y	Y	Y	Y	Y	Y	Y	Low risk
Akhter-Khan et al. (2023)	Y	Y	Y	Y	N	N	N	Y	Y	Y	Y	Y	Medium risk
Ayalon (2008)	Y	Y	Y	Y	Y	Y	N	Y	Y	Y	Y	Y	Medium risk
Barron et al. (2009)	Y	Y	N	Y	N	N	Y	Y	Y	Y	Y	Y	High risk
Bell et al. (2022)	Y	Y	N	Y	Y	Y	Y	Y	Y	Y	Y	Y	Low risk
Bell & Ferraro (2025)	Y	Y	N	Y	Y	Y	N	Y	N	CT	Y	CT	Medium risk
Bjalkebring et al. (2021)	Y	Y	N	Y	N	N	N	Y	Y	CT	Y	Y	High risk

Burr et al. (2016)	Y	Y	N	Y	Y	Y	Y	Y	Y	Y	Y	Y	Low risk
Carney (1987)	Y	Y	Y	Y	N	N	N	N	Y	Y	Y	Y	High risk
Carr (2018)	Y	Y	Y	Y	N	Y	Y	Y	Y	Y	Y	Y	Low risk
Chiao (2019)	Y	Y	N	Y	N	Y	N	Y	Y	CT	CT	Y	Medium risk
Cho & Xiang (2023)	Y	Y	Y	Y	N	Y	Y	Y	Y	Y	Y	Y	Low risk
Correa et al. (2022)	Y	Y	Y	Y	Y	Y	Y	Y	Y	Y	Y	Y	Low risk
Costenoble et al. (2023)	Y	Y	N	Y	N	N	N	N	Y	N	CT	CT	High risk
Dulin et al. (2012)	Y	Y	N	Y	N	N	N	Y	Y	CT	Y	CT	High risk
Fletcher et al. (2025)	Y	Y	Y	Y	N	N	N	N	Y	CT	Y	CT	High risk
Gil-Lacruz et al. (2019)	Y	Y	Y	Y	N	Y	Y	Y	Y	Y	Y	Y	Low risk
Griep et al. (2017)	Y	Y	N	N	N	Y	N	Y	Y	Y	Y	Y	High risk
Gonzales et al. (2019)	Y	Y	Y	Y	Y	Y	Y	Y	Y	Y	Y	Y	Low risk
Hambisa et al. (2022)	Y	N	N	Y	N	N	Y	Y	Y	CT	N	CT	High risk
Han et al. (2020)	Y	Y	Y	Y	N	Y	Y	Y	Y	Y	Y	Y	Low risk
Han & Park (2024) q	Y	Y	Y	N	N	Y	Y	Y	Y	Y	Y	Y	Medium risk
Han et al. (2017)	Y	Y	N	Y	Y	Y	Y	Y	Y	Y	Y	Y	Low risk
Harris & Thoresen (2005)	Y	Y	Y	Y	N	Y	Y	Y	Y	Y	Y	Y	Low risk

Hayward & Krause (2014)	Y	Y	N	Y	N	N	N	Y	Y	CT	Y	CT	High risk
Huo et al. (2021)	Y	Y	Y	Y	N	Y	Y	Y	Y	CT	Y	Y	Medium risk
Huo, Kim, Haghighat (2023)	Y	Y	Y	Y	N	Y	Y	Y	Y	CT	Y	Y	Medium risk
Huo, Kim, Wang (2023)	Y	Y	Y	Y	N	Y	Y	Y	Y	CT	Y	Y	Medium risk
Huo & Kim (2022)	Y	Y	N	Y	N	Y	Y	Y	Y	CT	Y	Y	Medium risk
Ide et al. (2023)	Y	Y	N	Y	N	Y	Y	Y	Y	Y	Y	Y	Medium risk
Infurna et al. (2016)	Y	Y	Y	Y	N	Y	Y	Y	Y	Y	Y	Y	Low risk
Jiang (2022)	Y	Y	Y	Y	Y	Y	Y	Y	N	CT	CT	Y	Medium risk
Jiang et al. (2019)	Y	Y	N	Y	N	N	CT	Y	Y	CT	CT	Y	High risk
Jirovec & Hyduk (1999)	Y	Y	Y	Y	Y	Y	Y	Y	Y	Y	Y	Y	Low risk
Johnson (2013)	Y	Y	Y	Y	Y	Y	Y	Y	Y	Y	Y	Y	Low risk
Jongenelis, Jackson, Warburton et al. (2022)	Y	Y	Y	Y	N	Y	Y	N	Y	Y	Y	Y	Medium risk
Kail & Carr (2020)	Y	Y	N	Y	N	N	Y	Y	Y	Y	Y	Y	Medium risk
Kim et al. (2020)	Y	Y	N	Y	N	Y	N	Y	Y	Y	Y	Y	High risk
Kim, Halvorsen et al. (2025)	Y	Y	Y	Y	Y	Y	Y	Y	Y	Y	Y	Y	Low risk

Kim, Shiba et al. (2025)	Y	Y	Y	Y	Y	Y	Y	Y	Y	Y	Y	Y	Low risk
Kim & Halvorsen (2021)	Y	Y	Y	N	Y	Y	Y	Y	Y	Y	Y	Y	Low risk
Kim & Yoon (2020)	Y	Y	N	Y	Y	Y	Y	Y	Y	Y	Y	Y	Low risk
Konrath et al. (2012)	Y	Y	N	Y	N	Y	Y	Y	Y	Y	Y	Y	Medium risk
Krause (2009)	Y	Y	N	N	N	Y	N	Y	Y	CT	Y	CT	High risk
Kuang et al. (2023)	Y	Y	N	N	N	Y	Y	N	Y	Y	Y	CT	Medium risk
Lakomy (2023)	Y	Y	N	Y	N	N	Y	Y	Y	Y	Y	Y	High risk
K. Lee et al. (2021)	Y	Y	Y	N	Y	Y	Y	Y	Y	Y	Y	Y	Low risk
Lee et al. (2011)	Y	Y	Y	Y	N	Y	Y	N	Y	Y	Y	Y	Medium risk
Lee (2019)	Y	Y	Y	Y	Y	Y	Y	Y	Y	Y	Y	Y	Low risk
Lee (2023)	Y	Y	Y	Y	Y	Y	Y	Y	Y	Y	Y	Y	Low risk
Lee et al. (2025)	Y	Y	N	N	N	Y	Y	Y	Y	Y	Y	Y	Medium risk
Li & Ferraro (2005)	Y	Y	N	Y	N	Y	Y	Y	Y	Y	Y	Y	Medium risk
Li et al. (2013)	Y	Y	N	Y	N	Y	Y	Y	Y	Y	Y	Y	Medium risk
Lim et al. (2023)	Y	Y	Y	Y	Y	Y	Y	Y	Y	Y	Y	Y	Low risk
Lim et al. (2025)	Y	Y	N	Y	N	Y	Y	Y	Y	Y	Y	Y	Medium risk
Luhr et al. (2022)	Y	Y	Y	Y	N	N	Y	Y	Y	CT	CT	CT	High risk

Lum & Lightfoot (2005)	Y	Y	Y	Y	N	Y	Y	Y	Y	Y	Y	Y	Low risk
Matthews & Nazroo (2021)	N	Y	N	Y	N	N	Y	Y	Y	N	Y	Y	High risk
Musick et al. (1999)	Y	Y	N	Y	Y	Y	Y	Y	Y	Y	Y	Y	Medium risk
Musick & Wilson (2003)	Y	Y	N	Y	Y	Y	N	Y	Y	Y	Y	Y	Medium risk
Nakamura et al. (2025)	Y	Y	Y	N	Y	Y	Y	Y	Y	Y	Y	Y	Low risk
Newman et al. (1995)	Y	CT	CT	Y	N	N	Y	N	N	CT	Y	CT	High risk
Nonaka et al. (2017)	Y	Y	N	Y	N	Y	Y	Y	Y	Y	Y	Y	High risk
Oman et al. (1999)	Y	Y	N	Y	N	Y	N	Y	Y	Y	Y	Y	Medium risk
O’Reilly et al. (2017)	Y	Y	N	Y	N	Y	Y	N	Y	CT	Y	Y	High risk
Parkinson et al. (2010)	Y	Y	N	Y	N	Y	Y	Y	Y	Y	Y	Y	Medium risk
Proulx et al. (2018)	Y	Y	Y	N	Y	Y	Y	Y	Y	Y	Y	Y	Low risk
Rogers et al. (2016)	Y	Y	Y	N	Y	Y	Y	Y	Y	Y	Y	Y	Low risk
Sabin (1993)	Y	Y	N	Y	N	N	N	Y	Y	CT	Y	Y	High risk
Sanchez-Garcia et al. (2025)	Y	Y	N	Y	N	N	Y	Y	Y	Y	Y	Y	High risk
Seeman et al. (2020)	Y	Y	Y	Y	N	N	N	Y	Y	Y	Y	Y	High risk
Sneed & Cohen (2013)	Y	Y	N	Y	N	N	N	Y	Y	CT	Y	CT	High risk

Sung et al. (2023)	Y	Y	N	Y	N	Y	Y	Y	Y	CT	Y	CT	Medium risk
Tamura et al. (2021)	Y	Y	Y	Y	N	Y	Y	Y	Y	CT	Y	Y	Medium risk
Tamura et al. (2024)	Y	Y	Y	Y	Y	Y	Y	Y	Y	CT	Y	Y	Medium risk
Tan et al. (2009)	Y	Y	Y	Y	N	N	Y	Y	N	CT	Y	CT	High risk
Tang (2009)	Y	Y	N	Y	N	N	N	Y	Y	CT	Y	Y	High risk
Taylor et al. (2024)	Y	Y	N	Y	N	N	Y	Y	N	CT	Y	CT	High risk
Torres et al. (2023)	Y	Y	N	Y	Y	Y	Y	Y	Y	Y	Y	Y	Low risk
Varma et al. (2016)	Y	Y	Y	Y	N	N	Y	Y	Y	Y	Y	Y	Medium risk
Webster et al. (2021)	Y	Y	Y	Y	Y	Y	Y	Y	Y	Y	Y	Y	Low risk
Weziak-Bialowolska et al. (2024)	Y	Y	Y	Y	Y	Y	Y	Y	Y	Y	Y	Y	Low risk
Windsor et al. (2008)	Y	Y	N	Y	N	N	Y	Y	Y	Y	Y	Y	Medium risk
Yamazaki et al. (2021)	Y	Y	Y	Y	Y	Y	Y	Y	Y	Y	Y	Y	Low risk
Yang (2020)	Y	Y	Y	Y	N	N	N	N	Y	Y	Y	Y	Medium risk
Xi et al. (2025)	Y	N	N	Y	N	Y	Y	Y	Y	Y	Y	Y	Medium risk.

Cross Sectional (Yes (Y) / No (N) / Can't Tell (CT)):

Author (Year)	Did the study	Did the authors use an	Were the subjects	Were the measures	Were the data collected in a	Did the study have enough	Was the data analysis	Is there a clear	Can the results be	Overall

	address a clearly focused issue?	appropriate method to answer their question?	recruited in an acceptable way?	accurately measured to reduce bias?	way that addressed the research issue?	participants to minimise the play of chance?	sufficiently rigorous?	statement of findings?	applied to the local population?	
Celdran & Villar (2007)	N	Y	Y	N	Y	N	Y	Y	Y	Medium risk
Chang et al. (2022)	Y	Y	Y	Y	Y	Y	Y	Y	Y	Low risk
Cheung & Kwan (2006)	Y	Y	Y	N	Y	Y	Y	Y	Y	Medium risk
Chu & Koo (2023)	Y	Y	Y	Y	Y	Y	Y	Y	Y	Low risk
Connolly & O'Shea (2015)	Y	Y	Y	N	Y	N	N	Y	Y	High risk
Fujii et al. (2024)	Y	N	Y	Y	Y	Y	Y	Y	Y	Low risk
Ge et al. (2025)	Y	Y	Y	N	Y	N	N	Y	Y	High risk<br>- Only 26.5% of the sample had volunteered
Greenfield & Marks (2004)	Y	Y	Y	Y	Y	Y	Y	Y	Y	Low risk
Guiney (2021)	Y	Y	Y	Y	Y	N	Y	Y	Y	Medium risk
Hidalgo et al. (2013)	Y	Y	Y	N	Y	Y	Y	Y	Y	Medium risk
Ho et al. (2018)	Y	Y	Y	Y	Y	Y	Y	Y	Y	Low risk
Huang et al. (2019)	Y	Y	Y	N	Y	Y	N	Y	CT	High risk
Hung et al. (2022)	Y	Y	Y	Y	Y	N	N	Y	Y	Medium risk
Jung et al. (2023)	Y	Y	Y	Y	Y	Y	Y	Y	Y	Low risk

Kim & Pan (2025)	Y	Y	Y	Y	Y	Y	Y	Y	Y	Low risk
Klinedinst & Resnick (2014)	Y	Y	Y	Y	Y	Y	Y	Y	Y	Low risk
Lam et al. (2023)	Y	Y	Y	Y	Y	Y	N	Y	Y	Medium risk – limited confounding factors considered
Lee (2024)	Y	Y	Y	Y	Y	Y	Y	Y	Y	Low risk
Lee & Kim (2014)	Y	Y	Y	N	Y	Y	N	Y	Y	Medium risk
Ling et al. (2023)	Y	N	Y	N	N	Y	N	Y	Y	High risk
Liu et al. (2020)	Y	Y	Y	N	Y	Y	N	Y	Y	Medium risk
Lyons et al. (2021)	Y	Y	Y	Y	Y	Y	Y	Y	Y	Low risk
Mayers et al. (2024)	Y	Y	Y	N	Y	Y	Y	Y	Y	Medium risk – volunteering not clearly defined
Mechakra-Tahiri et al. (2010)	Y	Y	Y	Y	Y	Y	Y	Y	Y	Low risk
Moncayo-Hernandez et al. (2024)	Y	Y	Y	Y	Y	Y	Y	Y	Y	Medium risk – Authors reported that majority of participants from cities with environmental barriers that may have resulted in increased falls
Monserud (2025)	Y	Y	Y	Y	N	Y	Y	Y	Y	Medium risk – multiple analyses performed, increased risk of Type I errors
Morrow-Howell (1999)	Y	Y	Y	N	Y	Y	N	Y	Y	High risk – results only presented as percentages

Morrow-Howell et al. (2009)	Y	Y	Y	N	Y	Y	N	Y	Y	High risk – would have benefited from additional analyses. Only 13 out of initial 51 volunteer programs were included in this study.
Myers et al. (2013)	Y	Y	Y	N	Y	Y	Y	Y	Y	Medium risk
Nakamura et al. (2023)	Y	Y	Y	Y	Y	Y	Y	Y	Y	Low risk
Pavlova & Silbereisen (2012)	Y	Y	Y	Y	Y	Y	Y	Y	Y	Low risk
Resnick et al. (2013)	Y	Y	Y	Y	Y	Y	Y	Y	Y	Low risk
Ryu & Heo (2018)	Y	Y	Y	Y	Y	Y	N	Y	Y	High risk
Shmotkin et al. (2003)	Y	Y	Y	Y	Y	Y	Y	Y	Y	Low risk
Tomioka et al. (2016)	Y	Y	Y	N	Y	Y	Y	Y	Y	Medium risk
Voloshina et al. (2023)	Y	Y	CT	CT	CT	N	CT	N	CT	High risk – critical appraisal may be limited by the quality of the English language translation of this paper
Wang et al. (2021)	Y	Y	Y	Y	Y	Y	Y	Y	Y	Low risk
Warburton et al. (2001)	Y	Y	Y	Y	Y	Y	Y	Y	Y	Low risk
Ward (2024)	Y	Y	Y	N	Y	Y	Y	Y	Y	Medium risk
Wu et al. (2005)	Y	Y	Y	Y	Y	Y	N	Y	Y	High risk
Xie & Han (2024)	Y	Y	Y	Y	Y	Y	Y	Y	Y	Low risk

Case Control (Yes (Y) / No (N) / Can't Tell (CT)):

Author (Year)	Did the study address a clearly focused issue?	Did the authors use an appropriate method to answer their question?	Were the cases recruited in an acceptable way?	Were the controls selected in an acceptable way?	Was the exposure accurately measured to minimise bias?	Aside from the experimental intervention, were the groups treated equally?	Have the authors taken account of the potential confounding factors in the design and/or in their analysis?	Was the exposure effect large?	Was the estimate of the exposure effect precise?	Do you believe the results?	Can the results be applied to the local population?	Overall
Rook & Sorkin (2003)	Y	Y	Y	Y	N	Y	N	CT	N	CT	CT	High risk
Shimanuki et al. (2007)	Y	Y	Y	Y	Y	Y	N	Y	Y	Y	Y	Medium risk
Warburton & Peel (2008)	Y	Y	Y	Y	N	Y	Y	Y	Y	Y	Y	Low risk

Qualitative (Yes (Y) / No (N) / Can't Tell (CT)):

Author (Year)	Was there a clear statement of the aims of the research?	Is qualitative methodology appropriate?	Was the research design appropriate to address the aims of the research?	Was the recruitment strategy appropriate to the aims of the research?	Was the data collected in a way that addressed the	Has the relationship between the researcher and participants been	Have ethical considerations been taken into consideration?	Was the data analysis sufficiently rigorous?	Is there a clear statement of findings?	Can the results be applied to the local population?	Overall

					research issue?	adequately considered?					
Breheny et al. (2020)	Y	Y	Y	Y	Y	CT	Y	Y	Y	Y	Low risk
Cao et al. (2021)	Y	Y	Y	Y	Y	CT	CT	Y	Y	Y	Low risk
Chen (2016)	Y	Y	Y	Y	Y	CT	Y	Y	Y	Y	Low risk
Fields (2023)	Y	Y	Y	Y	Y	CT	CT	Y	Y	Y	Low risk
Han et al. (2019)	Y	Y	Y	Y	Y	CT	Y	Y	Y	Y	Low risk
Han & Zhang (2025)	Y	Y	Y	Y	Y	CT	CT	Y	Y	Y	Low risk
Hwan & Hussin (2022)	Y	Y	Y	Y	Y	CT	CT	Y	Y	Y	Low risk
Jones & Reynolds (2019)	Y	Y	Y	Y	Y	CT	Y	Y	Y	Y	Low risk
Labegalini et al. (2015)	Y	Y	Y	Y	Y	CT	Y	Y	Y	Y	Low risk
Landry (2017)	Y	Y	Y	Y	Y	Y	Y	Y	Y	Y	Low risk
Y. Lee et al. (2021)	Y	Y	Y	Y	Y	CT	CT	Y	Y	Y	Low risk
Matsuda et al. (2024)	Y	Y	Y	Y	Y	CT	CT	Y	Y	Y	Low risk
Misener et al. (2010)	Y	Y	Y	Y	Y	CT	Y	Y	Y	Y	Low risk
Mukherjee (2010)	Y	Y	Y	Y	Y	CT	CT	Y	Y	Y	Low risk

Sellon (2018)	Y	Y	Y	Y	Y	Y	Y	Y	Y	Y	Low risk
Sellon (2023)	Y	Y	Y	Y	Y	Y	Y	Y	Y	Y	Low risk
Stathi et al. (2021)	Y	Y	Y	Y	Y	CT	Y	Y	Y	Y	Low risk
Stewart et al. (2024)	Y	Y	Y	Y	Y	CT	CT	Y	Y	Y	Low risk
Tiittanen & Turjamaa (2022)	Y	Y	Y	Y	Y	CT	Y	Y	Y	Y	Low risk
Varma et al. (2015)	Y	Y	Y	Y	Y	CT	CT	Y	Y	Y	Low risk
Warburton & Winterton (2017)	Y	Y	Y	Y	Y	CT	CT	Y	Y	Y	Low risk
White et al. (2025)	Y	Y	Y	Y	Y	CT	CT	Y	Y	Y	Low risk
Withall et al. (2018)	Y	Y	Y	Y	Y	CT	CT	Y	Y	Y	Low risk

Supplementary Material 4 – Biological Benefits and Harms of Older Adult Volunteering

Author (Year)	Design, Level of Evidence (I-VII)	Improved Physical Health	Increased Physical Activity	Improved Physical Fitness	Increased Strength	Greater Independence with ADLs/	Greater Independence with IADLs	Decreased Body Weight/ Obesity	Improved Walking Endurance	Increased Walking Speed	Increased Stair Climbing Speed	Reduced Chronic Health Condition Incidence	Reduced Falls Risk	Increased Dynamic Balace	Reduced Hip Fracture Risk	Reduced Mortality Rate	Improved Cardiovascular Health	Reduced Lipid Dysregulation	Reduced Hypertension Risk	Reduced CRP levels	Decreased Blood Sugar Levels	Reduced Epigenetic Age	Reduced CRTA Gene Expression	Increased brain volume	Reduced Dysphagia Risk	Healthier Eating Habits	Improved Sleep Quality	Reduced Binge Drinking	Reduced Smoking	Reduced Frailty Risk
Abe et al. (2022)	Cohort, IV		✓																							✓				
Abe et al. (2023)	Cohort, IV											✓				✓														
Ayalon (2008)	Cohort, IV															✓														
Barron et al. (2009)	Cohort, IV		✓		?				?	?	?																			
Bell et al. (2022)	Cohort, IV																			✓										
Bell & Ferraro (2025)	Cohort, IV																✓													
Brown et al. (2009)	Mixed Methods, IV	?				?																								
Burr et al. (2016)	Cohort, IV							?										✗	✓	?	?									
Carlson et al. (2015) (Men)[a]	RCT, II																							✓						
Carlson et al. (2015) (Women)[a]	RCT, II																							?						
Celdran & Villar (2007)	Cross Sectional, IV		✓																											
Chang et al. (2022)	Cross Sectional, IV	✓																												
Costenoble et al. (2023)	Cohort, IV																													?
De Souza et al. (2011)	Quasi-Experimental, III	?																												

Fried et al. (2004)[a]	RCT, II		✓		✓					✓																				
Gagliardi et al. (2020)	Mixed Methods, IV		✓																											
Gonzales et al. (2019)	Cohort, IV					✓	✓																							
Guiney et al. (2021)	Cross Sectional, IV		?														✓													
Han et al. (2017)	Cohort, IV															✓														
Han & Park (2024)	Cohort, IV																													✓
Harris & Thoresen (2005)	Cohort, IV															✓														
Hayward & Krause (2014)	Cohort, IV					✓																								
Ho et al. (2018)	Cross Sectional, IV		✓																											
Hong & Morrow-Howell (2010)	Quasi-Experimental, III					✓																								
Hsiao et al. (2020)	Quasi-Experimental, III							?									?		✓	✓	?									
Hung et al. (2022)	Cross Sectional, IV		✓		✓																									
Ide et al. (2023)	Cohort, IV					✓																								
Jirovec & Hyduk (1999) (50 – 99 hours per year)	Cohort, IV		?			✓						✓				✓											?			
Jirovec & Hyduk (1999) (100+ hours per year)	Cohort, IV		✓			✓						?				✓											?			
Kim et al. (2020) (50 – 99 hours	Cohort, IV		?			✓		?				✓				✓											?	?	?	

per year)																														
Kim et al. (2020) (100+ hours per year)	Cohort, IV		✓			✓		?				?				✓											?	?	?	
Kim et al. (2025) – Epigenetic Age	Cohort, IV																					✓								
Kim et al. (2025) - Hypertension	Cohort, IV																		✓											
Kim & Yoon (2020)	Cohort, IV																			✓							?			
Klinedinst & Resnick (2014)	Cross Sectional, IV					✓																								
Konrath et al. (2012)	Cohort, IV															✓														
Kuang et al. (2023)	Cohort, IV									?																				
Lam et al. (2023)	Cross Sectional, IV				?																									
Landry (2017)	Interview, VI		✓																											
K. Lee et al. (2021)	Cohort, IV	✓																												
Lee et al. (2011)	Cohort, IV															✓														
Li et al. (2013)	Cohort, IV					✓																								
Liu et al. (2020)	Cohort, IV	✓				✓																								
Lum & Lightfoot (2005)	Cohort, IV					✓	✓									✓														
Mayers et al. (2024)	Cross Sectional, IV		?																						?		?			?
Misener et al. (2010)	Interview, VI	✓	✓																											
Moncayo-Hernandez et	Cross Sectional, IV												✗																	

al. (2024)																														
Morrow -Howell et al. (1999)	Cross Sectiona l, IV	?																												
Morrow -Howell et al. (2009)	Cross Sectiona l, IV					?																								
Musick et al. (2019)	Cohort, IV															✓														
Nakamu ra et al. (2025)	Cohort, IV															✓						?								
Newma n (1983)	Mixed Methods , IV	?																												
Nonaka et al. (2017)	Cohort, IV					✓																								
Oman et al. (1999)	Cohort, IV															✓														
Parisi et al. (2015)[a]	RCT, II		✓																											
Parkins on et al. (2010)	Cohort, IV	✓																												
Pettigre w et al. (2020)	RCT, II		?	?	?			?	?	?		?		?																
Resnick et al. (2013)	Cross Sectiona l, IV		?									✓																		
Rogers et al. (2016)	Cohort, IV					✓										✓														
Sabin (1993)	Cohort, IV															✓														
Seeman et al. (2020)	Cohort, IV																						✓							
Sellon (2018)	Intervie w, VI		✓			✓																								
Sellon (2023)	Intervie w, VI	✓																												
Shiman uki et al. (2007)	Case Control, IV		✓	✓		✓	✓																							

Shmotkin et al. (2003)	Cross Sectional, IV		✓									?				✓														
Sneed & Cohen (2013) (1 – 199 hours per year)	Cohort, IV					?													?											
Sneed & Cohen (2013) (200+ hours per year)	Cohort, IV					?													✓											
Tamura et al. (2024)	Cohort, IV					✓						✓				?			?											
Tan et al. (2006)[a]	RCT, II		?																											
Tan et al. (2009)	Cohort, IV		?																											
Tang (2009)	Cohort, IV					✓						?																		
Taylor et al. (2024)	Cohort, IV															✓														
Tomioka et al. (2016)	Cross Sectional, IV						✓																							
Varma et al. (2015)	Focus Group, VI					✓																								
Varma et al. (2016) (Women)	Cohort, IV		✓																											
Varma et al. (2016) (Men)	Cohort, IV		?																											
Warburton & Peel (2008)	Case Control, IV														✓															
Warburton & Winterton (2017)	Cross Sectional, IV		✓																											
Webster et al. (2021)	Cohort, IV	?																												

**Weziak-Bialowolska et al. (2024)**	**Cohort, IV**					✓						?					?		?											
**Wu et al. (2005)**	**Interview, VI**	✓																												
**Yamazaki et al. (2021)**	**Cohort, IV**					?																								
**Yasunaga et al. (2016)**	**Quasi-Experimental, III**					?																								
**Yeung (2025)**	**RCT, II**																										?			
**Yuen et al. (2008)**	**RCT, II**	✗																												

✓ = Significant differences favouring volunteering intervention or theme in qualitative analyses

? = Non-significant differences between volunteering and control

✗ = Significant differences favouring control

C = Paper reports specific cases indicating harms of volunteering intervention

[a] Papers report on the same study by Fried et al. (2004)

ADLs = Activities of Daily Living

CRP = C-Reactive Protein

CRTA = Conserved Transcriptional Response to Adversity

IADLs = Instrumental Activities of Daily Living

Supplementary Material 5 – Psychological Benefits and Harms of Older Adult Volunteering

Author (Year)	Design, Level of Evidence (I-VII)	Reduced Depression Symptoms	Increased Life Satisfaction	Improved Psychological Wellbeing	Greater Psychological Resources	Greater Self-Fulfilment	Increased Intellectual Activity	Improved Cognitive Functioning	Reduced Cognitive Impairment Incidence	Reduced Stress/ Anxiety	More Personal Growth/ Learning	Reduced Fear of Falling	Greater Resilience	Reduced Hopelessness	More Health Satisfaction	Improved Attitude Towards Aging	Deeper Faith Development	Less Sedentary Lifestyle	Greater Will to Live
Abe et al. (2022)	Cohort, IV						✓												
Bjalkebring et al. (2021) “Higher levels of volunteering” – specific hours not stated	Cohort, IV		✗																
Breheny et al. (2020)	Interview, VI															✓			
Brown et al. (2009)	Mixed Methods, IV			✓				✓											
Brydges et al. (2021)[a]	RCT, II							?											
Cao et al. (2021)	Focus Group, VI									✓	✓								
Carney et al. (1987)	Cohort, IV					✓													
Celdran & Villar (2007)	Cross Sectional, IV					✓													
Chang et al. (2022)	Cross Sectional, IV			✓															
Chen (2016)	Interview, VI					✓		✓			✓					✓			
Cheung & Kwan (2006)	Cross Sectional, IV		✓			✓													
Chiao (2019)	Cohort, IV							✓											

Chu & Koo (2023)	Cross Sectional, IV		✓																
Cohen-Mansfield & Jensen (2017)	Interview, VI			✓ C															
Connolly & O'Shea (2015)	Cross Sectional, IV					?													
Correa et al. (2022)	Cohort, IV							✓											
De Souza et al. (2011)	Quasi-Experimental, III			✓															
Dulin et al. (2012)	Cohort, IV			✓															
Fields et al. (2023)	Interview, VI					✓					✓								
Fletcher et al. (2025)	Cohort, IV							?											
Fraser et al. (2009)	Interview, VI					?													
Fried et al. (2004)[a]	RCT, II							?											
Fuhi (2024)	Cross Sectional, IV		✓																
Gagliardi et al. (2020)	Mixed Methods, IV		✓	✓		✓													
Ge et al. (2025)	Cross Sectional, IV		✓																
George & Singer (2011)	RCT, II	?				?		?		✓									
Gil-Lacruz et al. (2019) (Religious Volunteering)	Cohort, IV		✓	?															
Gil-Lacruz et al. (2019) (Social Awareness Volunteering)	Cohort, IV		?	✗															
Gil-Lacruz et al. (2019) (Other Volunteering)	Cohort, IV		✓	?															
Gonzales et al. (2019)	Cohort, IV	?																	

**Greenfield & Marks (2004)**	**Cross Sectional, IV**			✓		?													
**Griep et al. (2017)**	**Cohort, IV**							✓	✓										
**Guiney et al. (2021)**	**Cross Sectional, IV**							✓											
**Han et al. (2019)**	**Interview, VI**			✓		✓													
**Han et al. (2020)**	**Cohort, IV**							✓											
**Hidalgo et al. (2013)**	**Cross Sectional, IV**		✓	✓		✓													
**Hong & Morrow-Howell (2010)**	**Quasi-Experimental, III**	✓																	
**Hsiao et al. (2020)**	**Quasi-Experimental, III**	✓		✓		✓													
**Huang (2019)**	**Cohort, IV**		?	✓															
**Hunter & Linn (1981)**	**Cross Sectional, IV**		✓			?				✓									✓
**Huo et al. (2021) (< 100 hours per year)**	**Cohort, IV**	?														?			
**Huo et al. (2021) (100+ hours per year)**	**Cohort, IV**	?														✓			
**Huo, Kim, Haghighat (2023)**	**Cohort, IV**															✓			
**Huo, Kim, Wang (2023) (100+ hours per year)**	**Cohort, IV**	✓	✓																
**Huo & Kim (2022)**	**Cohort, IV**		✓																
**Hwan & Hussin (2022)**	**Interview, VI**			✓						✓									
**Infurna et al. (2016)**	**Cohort, IV**								✓										
**Jiang (2022)**	**Cohort, IV**	?																	
**Jiang et al. (2019)**	**Cohort, IV**		✓																
**Jirovec & Hyduk (1999) (50 – 99 hours per year)**	**Cohort, IV**	✓	?	✓		?			✓										

Jirovec & Hyduk (1999) (100+ hours per year)	Cohort, IV	✓	?	✓		✓			?										
Johnson (2013) (Recently Widowed)	Cohort, IV	?																	
Johnson (2013) (Not Recently Widowed)	Cohort, IV	✓																	
Jones & Reynolds (2019)	Interview, VI															✓			
Jongenelis, Jackson, Newton et al. (2022)[b]	RCT, II	?	✓	✓		✓					✓								
Jongenelis, Jackson, Warburton et al. (2022) (Overall volunteers)	Cohort, IV			?	✓														
Jongenelis, Jackson, Warburton et al. (2022) (Volunteers who felt overwhelmed)	Cohort, IV			✗	✗														
Jones & Reynolds (2019)	Interview, VI			✓															
Jung et al. (2023)	Cross Sectional, IV																?		
Kail & Carr (2020)	Cohort, IV							✓											
Kim et al. (2020) (50 – 99 hours per year)	Cohort, IV	✓	?	✓		?		✓						?					
Kim et al. (2020) (100+ hours per year)	Cohort, IV	✓	?	✓		✓		?						✓					
Kim & Halvorsen (2021)	Cohort, IV	✓																	
Kim & Pan (2025)	Cross Sectional, IV							✓											
Klinedinst & Resnick (2014)	Cross Sectional, IV	?				✓													
Labegalini et al. (2015)	Interview, VI		✓	✓							✓								
Lam et al. (2023)	Cross Sectional, IV	?	?					?											

**Landry (2017)**	**Interview, VI**		✓	✓		✓				C									
**Larkin et al. (2005)**	**Mixed Methods, IV**		✓	✓		✓													
**Lee (2019)**	**Cohort, IV**		?																
**Lee (2024)**	**Cross Sectional, IV**	✓	✓																
**K. Lee et al. (2021)**	**Cohort, IV**					✓		✓											
**Y. Lee et al. (2021)**	**Interview, VI**			✓		✓													
**Lee & Kim (2014)**	**Cohort, IV**	?																	
**Li et al. (2013)**	**Cohort, IV**	✓	✓																
**Li & Ferraro (2005)**	**Cohort, IV**	✓																	
**Lim et al. (2023)**	**Cohort, IV**	✓																	
**Lim et al. (2025)**	**Cohort, IV**							✓											
**Ling et al. (2023)**	**Cross Sectional, IV**			?															
**Liu et al. (2020)**	**Cohort, IV**	✓												✓					
**Luhr et al. (2022) (Non-Political Volunteering)**	**Cohort, IV**		?																
**Luhr et al. (2022) (Political Volunteering)**	**Cohort, IV**		?																
**Lum & Lightfoot (2005)**	**Cohort, IV**	✓																	
**Lyons et al. (2021) (Lesbian Women)**	**Cross Sectional, IV**			✓															
**Lyons et al. (2021) (Gay Men)**	**Cross Sectional, IV**			✓															
**Matthews & Nazroo (2021)**	**Cohort, IV**	✓	✓																

**Matsuda et al.(2024)**	**Interview, VI**			✓						C	✓								
**Mayers et al. (2024)**	**Cross Sectional, IV**	✓																	
**Mechakra-Tahiri et al. (2010) (Women)**	**Cross Sectional, IV**	?																	
**Mechakra-Tahiri et al. (2010) (Men)**	**Cross Sectional, IV**	✓																	
**Misener et al. (2010)**	**Interview, VI**			✓ C												✓			
**Monserud (2025) (Married Men)**	**Cross Sectional, IV**	?						✗											
**Monserud (2025) (Unmarried Women)**	**Cross Sectional, IV**	?						✓											
**Morrow-Howell et al. (1999)**	**Cross Sectional, IV**										?								
**Morrow-Howell et al. (2009)**	**Cross Sectional, IV**		?			?													
**Mukherjee (2010)**	**Interview, VI**			✓ C															
**Musick & Wilson (2003)**	**Cohort, IV**	✓																	
**Myers et al. (2013)**	**Cross Sectional, IV**					✓											✓		
**Newman (1983)**	**Mixed Methods (Interview Results), VI**		✓	✓		✓										✓			
**Newman (1983)**	**Mixed Methods (Questionnaire Results), IV**		?	?		?													
**Newman et al. (1985)**	**Mixed Methods, IV**		?	✓		✓													
**Newman et al. (1995)**	**Cohort, IV**	?						?											
**O'Shea (2006)**	**Mixed Methods, IV**		?	?		?				C									

**Pardasani (2018)**	**Focus Group, VI**			✓		✓													
**Parisi et al. (2015)[a]**	**RCT, II**						✓												
**Parkinson et al. (2010)**	**Cohort, IV**			✓															
**Pavlova & Silbereisen (2012)**	**Cross Sectional, IV**	?	✓	?															
**Pettigrew et al. (2020)[b]**	**RCT, II**	?	?	?		?					?								
**Proulx et al. (2018)**	**Cohort, IV**							✓											
**Resnick et al. (2013)**	**Cross Sectional, IV**	?										?	✓						
**Rook & Sorkin (2003)**	**Case Control, IV**	?				✓													
**Ryu & Heo (2018)**	**Cohort, IV**		✓																
**Sanchez-Garcia et al. (2025)**	**Cohort, IV**		✓	✓															
**Sellon (2018)**	**Interview, VI**					✓													
**Sellon (2023)**	**Interview, VI**					✓				✓									
**Serrat-Graboleda et al. (2021)**	**Mixed Methods, IV**		✓	✓ C		✓										✓			
**Shimanuki et al. (2007)**	**Case Control, IV**			?			✓	✓							✓				
**Shmotkin et al. (2003)**	**Cross Sectional, IV**	✓						✓											
**Sneed & Cohen (2013)**	**Cohort, IV**			✓															
**Stathi et al. (2021)**	**Interview, VI**					✓													
**Stewart et al. (2024)**	**Interview, VI**			✓		✓													
**Sun et al. (2025)**	**Mixed Methods, IV**			✓		✓													

**Sung et al. (2023)**	**Cohort, IV**										✓								
**Tamura et al. (2021)**	**Cohort, IV**	✓																	
**Tamura et al. (2024)**	**Cohort, IV**	✓	?	✓					✓									✓	
**Varma et al. (2015)**	**Focus Group, VI**			✓ C		✓				C									
**Voloshina et al. (2023)**	**Cross Sectional, IV**		?			?				?			?						
**Wang et al. (2021)**	**Cross Sectional, IV**	✓																	
**Warburton et al. (2001)**	**Cross Sectional, IV**		✓	✓		✓													
**Warburton & Winterton (2017)**	**Interview, VI**									✓ C									
**Ward (2024)**	**Cross Sectional, IV**																		✓
**Warner et al. (2024)[c]**	**RCT, II**	✓								✓									
**Webster et al. (2021) (101 – 300 hours per year)**	**Cohort, IV**	✓																	
**Webster et al. (2021) (Other hours per year)**	**Cohort, IV**	?																	
**Weziak-Bialowolska et al. (2024)**	**Cohort, IV**	?		✓				?	✓										
**Windsor et al. (2008) (< 100 hours per year)**	**Cohort, IV**	?		✓															
**Windsor et al. (2008) (100 - 799 hours per year)**	**Cohort, IV**	✓		✓															
**Windsor et al. (2008) (800+ hours per year)**	**Cohort, IV**	?		?															
**Withall et al. (2018)**	**Interview, VI**			✓		✓													

Wu et al. (2005)	Interview, VI		✓	✓		✓													
Xi et al. (2025)	Cohort, IV	✓																	
Xie & Han (2024)	Cross Sectional, IV		✓																
Yang (2020)	Cohort, IV	✓																	
Yasunaga et al. (2016)	Quasi-Experimental, III			?															
Yeung (2025)[c]	RCT, II	?	?	?						✗									
Yuen et al. (2008)	RCT, II	?	?																

✓ = Significant differences favouring volunteering intervention or theme in qualitative analyses

? = Non-significant differences between volunteering and control

✗ = Significant differences favouring control

C = Paper reports specific cases indicating harms of volunteering intervention

[a] Papers report on the same study by Fried et al. (2004)

[b] Papers report on the same study by Pettigrew et al. (2020)

[c] Papers report on the same study by Warner et al. (2024)

Supplementary Material 6 – Social Benefits and Harms of Older Adult Volunteering

Author (Year)	Design	Increased Social Connectedness	Increased Social Support	Greater Satisfaction with Interpersonal Relationships	More Active Social Role	Reduced Loneliness	More Compassion for Others	Continued Driving Ability	Greater Environmental QOL
Akhter-Khan et al. (2023) (0 – 99 hours volunteering)	Cohort, IV					✓			
Akhter-Khan (2023) (0 – 99 hours volunteering)	Cohort, IV					?			
Brown et al. (2009)	Mixed Methods, IV	✓							
Carr et al. (2018)	Cohort, IV					?			
Celdran & Villar (2007)	Cross Sectional, IV	✓							
Chen (2016)	Interview, VI						✓		
Cho & Xiang (2023)	Cohort, IV					✓			
Connolly & O'Shea (2015)	Cross Sectional, IV	?							
Davila (2018)	Cross Sectional, IV		✓						
De Souza et al. (2011)	Quasi-Experimental, III	✓							?
Fields et al. (2023)	Interview, VI	✓				✓			
Fraser et al. (2009)	Mixed Methods, IV	✓							
Fried et al. (2004)	RCT, II		✓						
Gagliardi et al. (2020)	Mixed Methods, IV	✓							

Guiney et al. (2021)	Cross Sectional, IV	✓							
Han et al. (2019)	Interview, VI	✓					✓		
Han & Zhang (2025)	Interview, VI	✓							
Hambisa et al. (2022)	Cohort, IV							✓	
Hidalgo et al. (2013)	Cross Sectional, IV	✓	✓						
Hsiao et al. (2020)	Quasi-Experimental, III						✓		
Jirovec & Hyduk (1999) (50 – 99 hours per year)	Cohort, IV		✓			?			
Jirovec & Hyduk (1999) (100+ hours per year)	Cohort, IV		✓			✓			
Jones & Reynolds (2019)	Interview, VI	✓							
Jongenelis, Jackson, Newton et al. (2022)[b]	RCT, II	?							
Kim et al. (2020) (50 – 99 hours per year)	Cohort, IV		?			?			
Kim et al. (2020) (100+ hours per year)	Cohort, IV		?			✓			
Kritz et al. (2021)	Interview, VI	✓					✓		
Labegalini et al. (2015)	Interview, VI	✓							
Lee (2023)	Cohort, IV					✓			
Lee (2024)	Cross Sectional, IV					✓			
Y. Lee et al. (2021)	Interview, VI	✓							
Lim et al. (2023)	Cohort, IV		✓						
Luhr et al. (2022) (Non-Political Volunteering)	Cohort, IV					?			
Luhr et al. (2022) (Political Volunteering)	Cohort, IV					✗			

Lyons et al. (2021) (Lesbian Women)	Cross Sectional, IV	?	?						
Lyons et al. (2021) (Gay Men)	Cross Sectional, IV	✓	✓						
Matthews & Nazroo (2021)	Cohort, IV					?			
Mayers et al. (2024)	Cross Sectional, IV					✓			
Misener et al. (2010)	Interview, VI	✓ C							
Morrow-Howell et al. (1999)	Cross Sectional, IV	?							
Morrow-Howell et al. (2009)	Cross Sectional, IV	?							
Mukherjee (2010)	Interview, VI	✓							
Newman (1983)	Mixed Methods (Questionnaire Results), IV	?							
Pardasani (2018)	Focus Group, VI	✓					✓		
Parkinson et al. (2010)	Cohort, IV		✓						
Pettigrew et al. (2020)[b]	RCT, II	?							
Rook & Sorkin (2003)	Case Control, IV	?				?			
Sellon (2018)	Interview, VI	✓							
Sellon (2023)	Interview, VI	✓					✓		
Serrat-Graboleda et al. (2021)	Mixed Methods, IV			?					
Shimanuki et al. (2007)	Case Control, IV		✓		✓				
Shmotkin et al. (2003)	Cross Sectional, IV	✓							
Stathi et al. (2021)	Interview, VI	✓							

Stewart et al. (2024)	Interview, VI	✓							
Sun et al. (2025)	Mixed Methods, IV	✓							
Tamura et al. (2024)	Cohort, IV	✓				?			
Tiittanen & Turjamaa (2022)	Interview, VI	✓							
Torres et al. (2023)	Cohort, IV					✓			
Varma et al. (2015)	Focus Group, VI	✓					✓		
Warburton et al. (2001)	Cross Sectional, IV	✓							
Warburton & Winterton (2017)	Interview, VI	✓							
Warner et al. (2024)[c]	RCT, II	✓	?			✓			
Withall et al. (2018)	Interview, VI	✓							
Weziak-Bialowolska et al. (2024)	Cohort, IV					?			
White et al. (2025)	Interview, VI	✓				✓			
Yasanuga et al. (2016)	Quasi-Experimental, III	?							
Yeung (2025)[c]	RCT, II	?	?			?			

✓ = Significant differences favouring volunteering intervention or theme in qualitative analyses

? = Non-significant differences between volunteering and control

✗ = Significant differences favouring control

C = Paper reports specific cases indicating harms of volunteering intervention

[b] Papers report on the same study by Pettigrew et al. (2020)

[c] Papers report on the same study by Warner et al. (2024)

Supplementary Material 7 – Intersect Biopsychosocial Benefits and Harms of Older Adult Volunteering

Author (Year)	Design	Improved Health	Improved Quality of Life	Reduced Pain	Increased Energy Levels	Less Fear of Harm
Barron et al. (2009)	Cohort, IV				?	
Brown et al. (2009)	Mixed Methods, IV	✓		?		
Celdran & Villar (2007)	Cross Sectional, IV				✗	
Chang et al. (2022)	Cross Sectional, IV	✓				
Cheung & Kwan (2006)	Cross Sectional, IV	✓				
Connolly & O'Shea (2015)	Cross Sectional, IV		?			
De Souza et al. (2011)	Quasi-Experimental, III		✓			
Gil-Lacruz et al. (2019) (Religious Volunteering)	Cohort, IV	?				
Gil-Lacruz et al. (2019) (Social Awareness Volunteering)	Cohort, IV	?				
Gil-Lacruz et al. (2019) (Other Volunteering)	Cohort, IV	✓				
Gonzales et al. (2019)	Cohort, IV	✓				
Hidalgo et al. (2013)	Cross Sectional, IV	✓				
Hong & Morrow-Howell (2010)	Quasi-Experimental, III	?				
Huang (2019)	Cohort, IV	✓				
Hwan & Hussin (2022)	Interview, VI	✓				
Jirovec & Hyduk (1999)	Cohort, IV	✓		?		

**Johnson (2013)**<br>**(Recently Widowed)**	**Cohort, IV**	?				
**Johnson (2013)**<br>**(Not Recently Widowed)**	**Cohort, IV**	✓				
**Jung et al. (2023)**	**Cross Sectional, IV**		✓			
**Kim et al. (2020)**	**Cohort, IV**	✓		?		
**Kim & Halvorsen (2021)**	**Cohort, IV**	✓				
**Krause (2009)**	**Cohort, IV**	✓				
**Lakomy (2023)**	**Cohort, IV**		✓			
**Lee (2023)**	**Cohort, IV**		✓			
**Lee (2024)**	**Cross Sectional, IV**	✓	✓			
**Y. Lee et al. (2021)**	**Interview, VI**			✓		
**Li et al. (2013)**	**Cohort, IV**	✓				
**Lum & Lightfoot (2005)**	**Cohort, IV**	✓				
**Lyons et al. (2021)**<br>**(Lesbian Women)**	**Cross Sectional, IV**	?				
**Lyons et al. (2021)**<br>**(Gay Men)**	**Cross Sectional, IV**	✓				
**Matthews & Nazroo (2021)**	**Cohort, IV**		✓			
**Morrow-Howell et al. (2009)**	**Cross Sectional, IV**	?				
**Newman (1983)**	**Mixed Methods (Questionnaire Results), IV**				?	
**Pettigrew et al. (2020)**	**RCT, II**		?			
**Resnick et al. (2013)**	**Cross Sectional, IV**			?		
**Ryu & Heo (2018)**	**Cohort, IV**	✓				

Sanchez-Garcia et al. (2025)	Cohort, IV	✓				
Sellon (2018)	Interview, VI			✓		
Serrat-Graboleda et al. (2021)	Mixed Methods, IV	✓				
Shimanuki et al. (2007)	Case Control, IV	✗				
Shmotkin et al. (2003)	Cross Sectional, IV	✓				
Stewart et al. (2024)	Interview, VI				✗	
Sun et al. (2025)	Mixed Methods, IV	✓				
Tang (2009)	Cohort, IV	✓				
Tamura et al. (2024)	Cohort, IV	✓				
Varma et al. (2015)	Focus Group, VI				✓	C
Weziak-Bialowolska et al. (2024)	Cohort, IV				✓	
Yasunaga et al. (2016)	Quasi-Experimental, III	✓				

✓ = Significant differences favouring volunteering intervention or theme in qualitative analyses

? = Non-significant differences between volunteering and control

✗ = Significant differences favouring control

C = Paper reports specific cases indicating harms of volunteering intervention

Supplementary Material 8 – PRISMA Checklist

Section and Topic	Item #	Checklist item	Location where item is reported
**TITLE**			
Title	1	Identify the report as a systematic review.	Methods
**ABSTRACT**			
Abstract	2	See the PRISMA 2020 for Abstracts checklist.	Abstract
**INTRODUCTION**			
Rationale	3	Describe the rationale for the review in the context of existing knowledge.	Background
Objectives	4	Provide an explicit statement of the objective(s) or question(s) the review addresses.	Background
**METHODS**			
Eligibility criteria	5	Specify the inclusion and exclusion criteria for the review and how studies were grouped for the syntheses.	Inclusion Criteria
Information sources	6	Specify all databases, registers, websites, organisations, reference lists and other sources searched or consulted to identify studies. Specify the date when each source was last searched or consulted.	Methods
Search strategy	7	Present the full search strategies for all databases, registers and websites, including any filters and limits used.	Supplementary Materials Table 1
Selection process	8	Specify the methods used to decide whether a study met the inclusion criteria of the review, including how many reviewers screened each record and each report retrieved, whether they worked independently, and if applicable, details of automation tools used in the process.	Data Extraction
Data collection process	9	Specify the methods used to collect data from reports, including how many reviewers collected data from each report, whether they worked independently, any processes for obtaining or confirming data from study investigators, and if applicable, details of automation tools used in the process.	Data Extraction
Data items	10a	List and define all outcomes for which data were sought. Specify whether all results that were compatible with each outcome domain in each study were sought (e.g. for all measures, time points, analyses), and if not, the methods used to decide which results to collect.	Outcomes
	10b	List and define all other variables for which data were sought (e.g. participant and intervention characteristics, funding sources). Describe any assumptions made about any missing or unclear information.	Data Extraction
Study risk of bias assessment	11	Specify the methods used to assess risk of bias in the included studies, including details of the tool(s) used, how many reviewers assessed each study and whether they worked independently, and if applicable, details of automation tools used in the process.	Risk of Bias Assessment Approach

Section and Topic	Item #	Checklist item	Location where item is reported
Effect measures	12	Specify for each outcome the effect measure(s) (e.g. risk ratio, mean difference) used in the synthesis or presentation of results.	Data Analysis
Synthesis methods	13a	Describe the processes used to decide which studies were eligible for each synthesis (e.g. tabulating the study intervention characteristics and comparing against the planned groups for each synthesis (item #5)).	Data Analysis
	13b	Describe any methods required to prepare the data for presentation or synthesis, such as handling of missing summary statistics, or data conversions.	Data Analysis
	13c	Describe any methods used to tabulate or visually display results of individual studies and syntheses.	Supplementary Materials
	13d	Describe any methods used to synthesize results and provide a rationale for the choice(s). If meta-analysis was performed, describe the model(s), method(s) to identify the presence and extent of statistical heterogeneity, and software package(s) used.	Data Analysis
	13e	Describe any methods used to explore possible causes of heterogeneity among study results (e.g. subgroup analysis, meta-regression).	P.3 Data Analysis
	13f	Describe any sensitivity analyses conducted to assess robustness of the synthesized results.	N/A
Reporting bias assessment	14	Describe any methods used to assess risk of bias due to missing results in a synthesis (arising from reporting biases).	Risk of Bias Assessment
Certainty assessment	15	Describe any methods used to assess certainty (or confidence) in the body of evidence for an outcome.	N/A
**RESULTS**			
Study selection	16a	Describe the results of the search and selection process, from the number of records identified in the search to the number of studies included in the review, ideally using a flow diagram.	Figure 1
	16b	Cite studies that might appear to meet the inclusion criteria, but which were excluded, and explain why they were excluded.	Figure 1
Study characteristics	17	Cite each included study and present its characteristics.	Supplementary Materials
Risk of bias in studies	18	Present assessments of risk of bias for each included study.	Supplementary Materials
Results of individual studies	19	For all outcomes, present, for each study: (a) summary statistics for each group (where appropriate) and (b) an effect estimate and its precision (e.g. confidence/credible interval), ideally using structured tables or plots.	Supplementary Materials
Results of syntheses	20a	For each synthesis, briefly summarise the characteristics and risk of bias among contributing studies.	Supplementary Materials
	20b	Present results of all statistical syntheses conducted. If meta-analysis was done, present for each the summary estimate and its precision (e.g. confidence/credible interval) and measures of statistical heterogeneity. If comparing groups, describe the direction of the effect.	N/A
	20c	Present results of all investigations of possible causes of heterogeneity among study results.	Supplementary Materials

Section and Topic	Item #	Checklist item	Location where item is reported
	20d	Present results of all sensitivity analyses conducted to assess the robustness of the synthesized results.	N/A
Reporting biases	21	Present assessments of risk of bias due to missing results (arising from reporting biases) for each synthesis assessed.	Supplementary Materials
Certainty of evidence	22	Present assessments of certainty (or confidence) in the body of evidence for each outcome assessed.	N/A
**DISCUSSION**			
Discussion	23a	Provide a general interpretation of the results in the context of other evidence.	Results
	23b	Discuss any limitations of the evidence included in the review.	Discussion
	23c	Discuss any limitations of the review processes used.	Discussion
	23d	Discuss implications of the results for practice, policy, and future research.	Discussion
**OTHER INFORMATION**			
Registration and protocol	24a	Provide registration information for the review, including register name and registration number, or state that the review was not registered.	Methods
	24b	Indicate where the review protocol can be accessed, or state that a protocol was not prepared.	Methods
	24c	Describe and explain any amendments to information provided at registration or in the protocol.	Risk of Bias Assessment
Support	25	Describe sources of financial or non-financial support for the review, and the role of the funders or sponsors in the review.	Funding Statement
Competing interests	26	Declare any competing interests of review authors.	Conflict of Interest Statement
Availability of data, code and other materials	27	Report which of the following are publicly available and where they can be found: template data collection forms; data extracted from included studies; data used for all analyses; analytic code; any other materials used in the review.	Data Availability Statement

From: Page MJ, McKenzie JE, Bossuyt PM, Boutron I, Hoffmann TC, Mulrow CD, et al. The PRISMA 2020 statement: an updated guideline for reporting systematic reviews. BMJ 2021;372:n71. doi: 10.1136/bmj.n71. This work is licensed under CC BY 4.0. To view a copy of this license, visit https://creativecommons.org/licenses/by/4.0/

Supplementary References

Abe, N., Ide, K., Watanabe, R., Hayashi, T., Iizuka, G., & Kondo, K. (2023). Social participation and incident disability and mortality among frail older adults: a JAGES longitudinal study. Journal of the American Geriatrics Society, 71(6), 1881-1890. https://doi.org/10.1111/jgs.18269

Abe, T., Seino, S., Tomine, Y., Nishi, M., Hata, T., Shinkai, S., Fujiwara, Y., & Kitamura, A. (2022). Identifying the specific associations between participation in social activities and healthy lifestyle behaviours in older adults. Maturitas, 155, 24-31. https://doi.org/10.1016/j.maturitas.2021.10.003

Akhter-Khan, S. C., Hofmann, V., Warncke, M., Tamimi, N., Mayston, R., & Prina, M. A. (2023). Caregiving, volunteering, and loneliness in middle-aged and older adults: a systematic review. Aging & Mental Health, 27(7), 1233-1245. https://doi.org/10.1080/13607863.2022.2144130

Anderson, N. D., Damianakis, T., Kröger, E., Wagner, L. M., Dawson, D. R., Binns, M. A., Bernstein, S., Caspi, E., & Cook, S. L. (2014). The benefits associated with volunteering among seniors: a critical review and recommendations for future research. Psychological bulletin, 140(6), 1505. https://doi.org/10.1037/a0037610

Ayalon, L. (2008). Volunteering as a predictor of all-cause mortality: what aspects of volunteering really matter? International Psychogeriatrics, 20(5), 1000-1013. https://doi.org/10.1017/S1041610208007096

Barron, J. S., Tan, E. J., Yu, Q., Song, M., McGill, S., & Fried, L. P. (2009). Potential for intensive volunteering to promote the health of older adults in fair health. Journal of Urban Health, 86(4), 641-653. https://doi.org/10.1007/s11524-009-9353-8

Bell, M. J., & Ferraro, K. F. (2025). Volunteering and Risk of Heart Attack in Later Life: The Moderating Role of Purpose in Life? Research on aging, 47(2), 140-150. https://doi.org/10.1177/01640275241274316

Bell, M. J., Ferraro, K. F., & Sauerteig-Rolston, M. R. (2022). Volunteer engagement and systemic inflammation: Does helping others benefit oneself? The Gerontologist, 62(10), 1477-1485. https://doi.org/10.1093/geront/gnac073

Bjälkebring, P., Henning, G., Västfjäll, D., Dickert, S., Brehmer, Y., Buratti, S., Hansson, I., & Johansson, B. (2021). Helping out or helping yourself? Volunteering and life satisfaction across the retirement transition. Psychology and aging, 36(1), 119. https://doi.org/10.1037/pag0000576

Breheny, M., Pond, R., & Lilburn, L. E. (2020). "What am I going to be like when I'm that age?": How older volunteers anticipate ageing through home visiting. Journal of Aging Studies, 53, 100848. https://doi.org/10.1016/j.jaging.2020.100848

Brown, J. W., Mefford, L., Chen, S.-l., Callen, B., & Brown, A. (2009). Health and function of older persons volunteering for Habitat for Humanity. Southern Online Journal of Nursing Research, 9(3). https://www.researchgate.net/profile/Bonnie-Callen/publication/237780071_Health_and_function_of_older_persons_volunteering_for_Habitat_for_Humanity/links/55ea139c08ae65b6389c584e/Health-and-function-of-older-persons-volunteering-for-Habitat-for-Humanity.pdf

Brydges, C. R., Carlson, M. C., Andrews, R. M., Rebok, G. W., & Bielak, A. A. (2021). Using cognitive intraindividual variability to measure intervention effectiveness: Results from the Baltimore experience corps trial. The Journals of Gerontology: Series B, 76(4), 661-670. https://doi.org/10.1093/geronb/gbaa009

Burr, J. A., Han, S. H., & Tavares, J. L. (2016). Volunteering and cardiovascular disease risk: Does helping others get "under the skin?". The Gerontologist, 56(5), 937-947. https://doi.org/10.1093/geront/gnv032

Cao, Q., Dabelko-Schoeny, H. I., White, K., Maleku, A., & Sheldon, M. (2021). I wanna help, but my hands can be a little tied: The challenges and benefits of formal volunteering among low-income diverse older adults. Journal of Gerontological Social Work, 64(4), 388-404. https://doi.org/10.1080/01634372.2021.1897723

Carlson, M. C., Kuo, J. H., Chuang, Y.-F., Varma, V. R., Harris, G., Albert, M. S., Erickson, K. I., Kramer, A. F., Parisi, J. M., & Xue, Q.-L. (2015). Impact of the Baltimore Experience Corps Trial on cortical and hippocampal volumes. Alzheimer's & Dementia, 11(11), 1340-1348. https://doi.org/10.1016/j.jalz.2014.12.005

Carney, J. M., Dobson, J. E., & Dobson, R. L. (1987). Using senior citizen volunteers in the schools. Journal of Humanistic Counseling, Education & Development. https://psycnet.apa.org/doi/10.1002/j.2164-4683.1987.tb00305.x

Carr, D. C., Kail, B. L., Matz-Costa, C., & Shavit, Y. Z. (2018). Does becoming a volunteer attenuate loneliness among recently widowed older adults? The Journals of Gerontology: Series B, 73(3), 501-510. https://doi.org/10.1093/geronb/gbx092

Celdrán, M., & Villar, F. (2007). Volunteering among older Spanish adults: does the type of organization matter? Educational Gerontology, 33(3), 237-251. https://doi.org/10.1080/03601270601161181

Chang, H.-T., Chen, H.-C., Hsu, N.-W., & Chou, P. (2022). Volunteering and self-reported health outcomes among older people living in the community: The Yilan study, Taiwan. Quality of Life Research, 1-9. https://doi.org/10.1007/s11136-021-02933-y

Chen, L.-K. (2016). Benefits and dynamics of learning gained through volunteering: A qualitative exploration guided by seniors' self-defined successful aging. Educational Gerontology, 42(3), 220-230. https://doi.org/10.1080/03601277.2015.1108150

Chen, P.-W., Chen, L.-K., Huang, H.-K., & Loh, C.-H. (2022). Productive aging by environmental volunteerism: A systematic review. Archives of Gerontology and Geriatrics, 98, 104563. https://doi.org/10.1016/j.archger.2021.104563

Cheung, C.-k., & Kwan, A. Y.-h. (2006). Inducting older adults into volunteer work to sustain their psychological well-being. Ageing International, 31, 44-58. https://doi.org/10.1007/s12126-006-1003-9

Chiao, C. (2019). Beyond health care: Volunteer work, social participation, and late-life general cognitive status in Taiwan. Social science & medicine, 229, 154-160. https://doi.org/10.1016/j.socscimed.2018.06.001

Cho, J., & Xiang, X. (2023). The relationship between volunteering and the occurrence of loneliness among older adults: a longitudinal study with 12 years of follow-up. Journal of Gerontological Social Work, 66(5), 680-693. https://doi.org/10.1080/01634372.2022.2139322

Chu, J.-T., & Koo, M. (2023). Life satisfaction and self-esteem in older adults engaging in formal volunteering: A cross-sectional study in Taiwan. International journal of environmental research and public health, 20(6), 4934. https://doi.org/10.3390/ijerph20064934

Cnaan, R. A., Handy, F., & Wadsworth, M. (1996). Defining who is a volunteer: Conceptual and empirical considerations. Nonprofit and Voluntary Sector Quarterly, 25(3), 364-383. https://doi.org/10.1177/0899764096253006

Cohen-Mansfield, J., & Jensen, B. (2017). Intergenerational programs in schools: Prevalence and perceptions of impact. Journal of Applied Gerontology, 36(3), 254-276.

Commonwealth of Australia. (2009). Appendix F: Levels of evidence and recommendation grading. https://www.nhmrc.gov.au/sites/default/files/images/appendix-f-levels-of-evidence.pdf

Connolly, S., & O'shea, E. (2015). The perceived benefits of participating in voluntary activities among older people: Do they differ by volunteer characteristics? Activities, Adaptation & Aging, 39(2), 95-108. https://doi.org/10.1080/01924788.2015.1024075

Corrêa, J. C., Ávila, M. P. W., Lucchetti, A. L. G., & Lucchetti, G. (2022). Altruism, volunteering and cognitive performance among older adults: A 2-year longitudinal study. Journal of Geriatric Psychiatry and Neurology, 35(1), 66-77. https://doi.org/10.1177/0891988720964260

Costenoble, A., Knoop, V., Debain, A., Bautmans, I., Van Laere, S., Lieten, S., Rossi, G., Verté, D., Gorus, E., & De Vriendt, P. (2023). Transitions in robust and prefrail octogenarians after 1 year: the influence of activities of daily living, social participation, and psychological resilience on the frailty state. BMC geriatrics, 23(1), 485. https://doi.org/10.1186/s12877-023-04178-5

Critical Appraisal Skills Programme. (2023). CASP Checklists. https://casp-uk.net/casp-tools-checklists/

Dávila, M. C. (2018). The relationship between social networks and volunteerism among seniors. Journal of Social Service Research, 44(1), 38-49. https://doi.org/10.1080/01488376.2017.1395382

De Souza, L. M., Lautert, L., & Hilleshein, E. F. (2011). Quality of life and voluntary work among the elderly. Revista da Escola de Enfermagem da USP, 45, 665-671. https://doi.org/10.1590/S0080-62342011000300017

Dederichs, K. (2023). Volunteering in the United Kingdom during the COVID-19 pandemic: who started and who quit? Nonprofit and Voluntary Sector Quarterly, 52(5), 1458-1474. https://doi.org/10.1177/08997640221122814

Dulin, P. L., Gavala, J., Stephens, C., Kostick, M., & McDonald, J. (2012). Volunteering predicts happiness among older Māori and non-Māori in the New Zealand health, work, and retirement longitudinal study. Aging & Mental Health, 16(5), 617-624. https://doi.org/10.1080/13607863.2011.641518

Engel, G. L. (1977). The need for a new medical model: a challenge for biomedicine. Science, 196(4286), 129-136. https://doi.org/10.1126/science.847460

Fields, N. L., Lee, K., Cassidy, J., Kunz-Lomelin, A., Stringfellow, M. K., & Feinhals, G. (2023). It gave me somebody else to think about besides myself: caring callers volunteer experiences with a telephone-based reassurance program for socially isolated older adults. Journal of Applied Gerontology, 42(1), 49-58. https://doi.org/10.1177/07334648221123302

Filges, T., Siren, A., Fridberg, T., & Nielsen, B. C. (2020). Voluntary work for the physical and mental health of older volunteers: A systematic review. Campbell Systematic Reviews, 16(4), e1124. https://doi.org/10.1002/cl2.1124

Fletcher, E., Chanti-Ketterl, M., Hokett, E., Lor, Y., Venkatesan, U., Chen, R., Bubu, O. M., Whitmer, R., Gilsanz, P., & Zlatar, Z. Z. (2025). Modeling the importance of life exposure factors on memory performance in diverse older adults: A machine learning approach. Alzheimer's & Dementia, 21(8), e70428. https://doi.org/10.1002/alz.70428

Forman, J., & Damschroder, L. (2007). Qualitative content analysis. In Empirical methods for bioethics: A primer (pp. 39-62). Emerald Group Publishing Limited.

Fraser, J., Clayton, S., Sickler, J., & Taylor, A. (2009). Belonging at the zoo: Retired volunteers, conservation activism and collective identity. Ageing & Society, 29(3), 351-368. https://doi.org/10.1017/S0144686X08007915

Fried, L. P., Carlson, M. C., Freedman, M., Frick, K. D., Glass, T. A., Hill, J., McGill, S., Rebok, G. W., Seeman, T., & Tielsch, J. (2004). A social model for health promotion for an aging population: initial evidence on the Experience Corps model. Journal of Urban Health, 81, 64-78. https://doi.org/10.1093/jurban/jth094

Fried, L. P., Carlson, M. C., McGill, S., Seeman, T., Xue, Q.-L., Frick, K., Tan, E., Tanner, E. K., Barron, J., & Frangakis, C. (2013). Experience Corps: a dual trial to promote the health of older adults and children's academic success. Contemporary clinical trials, 36(1), 1-13. https://doi.org/10.1016/j.cct.2013.05.003

Fujii, K., Lee, S., Katayama, O., Makino, K., Harada, K., Tomida, K., Morikawa, M., Yamaguchi, R., Nishijima, C., & Misu, Y. (2024). Diversity in productive activities enhances life satisfaction among Japanese community-dwelling older adults: A cross-sectional study. Geriatric Nursing, 58, 232-237. https://doi.org/10.1016/j.gerinurse.2024.05.033

Gagliardi, C., Pillemer, K., Gambella, E., Piccinini, F., & Fabbietti, P. (2020). Benefits for older people engaged in environmental volunteering and socializing activities in city parks: Preliminary results of a program in Italy. International journal of environmental research and public health, 17(11), 3772. https://doi.org/10.3390/ijerph17113772

Ge, Y., Jiang, W., Xue, M., Lyu, Y., Hong, L., & Tian, K. (2025). The mediating effect of social networks and depressive symptoms on the relationship between older adults' participation in volunteer activities and life satisfaction: a cross-sectional study involving Chinese older adults. BMC geriatrics, 25(1), 678. https://doi.org/10.1186/s12877-025-06362-1

George, D. R., & Singer, M. E. (2011). Intergenerational volunteering and quality of life for persons with mild to moderate dementia: results from a 5-month intervention study in the United States. The American journal of geriatric psychiatry, 19(4), 392-396. https://doi.org/10.1097/JGP.0b013e3181f17f20

Gil-Lacruz, M., Saz-Gil, M. I., & Gil-Lacruz, A. I. (2019). Benefits of older volunteering on wellbeing: An international comparison. Frontiers in psychology, 10, 2647. https://doi.org/10.3389/fpsyg.2019.02647

Gonzales, E., Shen, H.-W., Perry, T. E., & Wang, Y. (2019). Intersections of home, health, and social engagement in old age: formal volunteering as a protective factor to health after relocation. Research on aging, 41(1), 31-53. https://doi.org/10.1177/0164027518773125

Greenfield, E. A., & Marks, N. F. (2004). Formal volunteering as a protective factor for older adults' psychological well-being. The Journals of Gerontology Series B: Psychological Sciences and Social Sciences, 59(5), S258-S264. https://doi.org/10.1093/geronb/59.5.S258

Griep, Y., Hanson, L. M., Vantilborgh, T., Janssens, L., Jones, S. K., & Hyde, M. (2017). Can volunteering in later life reduce the risk of dementia? A 5-year longitudinal study among volunteering and non-volunteering retired seniors. PloS one, 12(3), e0173885. https://doi.org/10.1371/journal.pone.0173885

Grotz, J., Dyson, S., & Birt, L. (2020). Pandemic policy making: the health and wellbeing effects of the cessation of volunteering on older adults during the COVID-19 pandemic. Quality in Ageing and Older Adults, 21(4), 261-269. https://doi.org/10.1108/QAOA-07-2020-0032

Guiney, H., Keall, M., & Machado, L. (2021). Volunteering in older adulthood is associated with activity engagement and cognitive functioning. Aging, Neuropsychology, and Cognition, 28(2), 253-269. https://doi.org/10.1080/13825585.2020.1743230

Guiney, H., & Machado, L. (2018). Volunteering in the community: Potential benefits for cognitive aging. The Journals of Gerontology: Series B, 73(3), 399-408. https://doi.org/10.1093/geronb/gbx134

Hambisa, M. T., Dolja-Gore, X., & Byles, J. E. (2022). Determinants of driving among oldest-old Australian women. Journal of Women & Aging, 34(3), 351-371. https://doi.org/10.1080/08952841.2021.1937012

Han, A., Brown, D., & Richardson, A. (2019). Older adults' perspectives on volunteering in an activity-based social program for people with dementia. Activities, Adaptation & Aging, 43(2), 145-163. https://doi.org/10.1080/01924788.2018.1500055

Han, M., & Zhang, Y. B. (2025). Who We Are and How We Talk About Volunteering: Older Adult Volunteers' Perspective. The International Journal of Aging and Human Development, 101(1), 68-89. https://doi.org/10.1177/00914150241297867

Han, S. H., Kim, K., & Burr, J. A. (2020). Stress-buffering effects of volunteering on daily well-being: Evidence from the National Study of Daily Experiences. The Journals of Gerontology: Series B, 75(8), 1731-1740. https://doi.org/10.1093/geronb/gbz052

Han, S. H., & Park, N. (2024). Heterogeneous effects of volunteering on frailty in later life: a panel quantile regression approach. The Journals of Gerontology, Series B: Psychological Sciences and Social Sciences, 79(5). https://doi.org/10.1093/geronb/gbae033

Han, S. H., Tavares, J. L., Evans, M., Saczynski, J., & Burr, J. A. (2017). Social activities, incident cardiovascular disease, and mortality: Health behaviors mediation. Journal of aging and health, 29(2), 268-288. https://doi.org/10.1177/0898264316635565

Harris, A. H., & Thoresen, C. E. (2005). Volunteering is associated with delayed mortality in older people: analysis of the longitudinal study of aging. Journal of Health Psychology, 10(6), 739-752. https://doi.org/10.1177/1359105305057310

Hayward, R. D., & Krause, N. (2014). Voluntary leadership roles in religious groups and rates of change in functional status during older adulthood. Journal of behavioral medicine, 37, 543-552. https://doi.org/10.1007/s10865-012-9488-z

Hidalgo, M. C., Moreno-Jiménez, P., & Quiñonero, J. (2013). Positive effects of voluntary activity in old adults. Journal of Community Psychology, 41(2), 188-199. https://doi.org/10.1002/jcop.21522

Ho, E. C., Hawkley, L., Dale, W., Waite, L., & Huisingh-Scheetz, M. (2018). Social capital predicts accelerometry-measured physical activity among older adults in the US: a cross-sectional study in the National Social Life, Health, and Aging Project. BMC public health, 18, 1-11. https://doi.org/10.1186/s12889-018-5664-6

Hong, S. I., & Morrow-Howell, N. (2010). Health outcomes of Experience Corps®: A high-commitment volunteer program. Social science & medicine, 71(2), 414-420. https://doi.org/10.1016/j.socscimed.2010.04.009

Hsiao, H.-Y., Hsu, C.-T., Chen, L., Wu, J., Chang, P.-S., Lin, C.-L., Lin, M.-N., & Lin, T.-K. (2020). Environmental volunteerism for social good: A longitudinal study of older adults' health. Research on Social Work Practice, 30(2), 233-245. https://doi.org/10.1177/1049731519892620

Huang, L.-H. (2019). Well-being and volunteering: Evidence from aging societies in Asia. Social science & medicine, 229, 172-180. https://doi.org/10.1016/j.socscimed.2018.09.004

Hung, C.-S., Loh, C.-H., Hsieh, J.-G., Chen, J.-C., Lin, Y.-W., & Yen, C.-F. (2022). The potential win-win strategy for healthy aging and environmental protection: Environmental volunteering. American Journal of Health Promotion, 36(3), 510-513. https://doi.org/10.1177/08901171211055599

Hunter, K. I., & Linn, M. W. (1981). Psychosocial differences between elderly volunteers and non-volunteers. The International Journal of Aging and Human Development, 12(3), 205-213. https://doi.org/10.2190/0H6V-QPPP-7JK4-LR38

Huo, M., & Kim, K. (2022). Volunteering dynamics and life satisfaction: Self-perceptions of aging as a buffer. The Journals of Gerontology: Series B, 77(2), 321-331. https://doi.org/10.1093/geronb/gbab104

Huo, M., Kim, K., & Wang, D. (2023). Long-term psychological consequences of parental bereavement prior to midlife: Volunteering helps. Aging & Mental Health, 27(5), 992-1000. https://doi.org/10.1080/13607863.2022.2087209

Huo, M., Miller, L. M. S., Kim, K., & Liu, S. (2021). Volunteering, self-perceptions of aging, and mental health in later life. The Gerontologist, 61(7), 1131-1140. https://doi.org/10.1093/geront/gnaa164

Hwan, N. L., & Hussin, N. A. M. (2022). Volunteering Experience among older adults with end-stage renal disease (ESRD). Journal of Gerontological Social Work, 65(3), 271-289. https://doi.org/10.1080/01634372.2021.1959478

Ide, K., Tsuji, T., Kanamori, S., Watanabe, R., Iizuka, G., & Kondo, K. (2023). Frequency of social participation by types and functional decline: A six-year longitudinal study. Archives of Gerontology and Geriatrics, 112, 105018. https://doi.org/10.1016/j.archger.2023.105018

Infurna, F. J., Okun, M. A., & Grimm, K. J. (2016). Volunteering is associated with lower risk of cognitive impairment. Journal of the American Geriatrics Society, 64(11), 2263-2269. https://doi.org/10.1111/jgs.14398

International Labour Organization and United Nations Volunteers. (2025). Volunteer work among older persons: Trends and policy implications for ageing societies Retrieved October from https://knowledge.unv.org/system/files/2025-04/Research%20paper_volunteer%20work%20among%20older%20persons.pdf

Jiang, D., Hosking, D., Burns, R., & Anstey, K. J. (2019). Volunteering benefits life satisfaction over 4 years: The moderating role of social network size. Australian Journal of Psychology, 71(2), 183-192. https://doi.org/10.1111/ajpy.12217

Jiang, N. (2022). Formal volunteering and depressive symptoms among Community-Dwelling older adults in China: A longitudinal cross-level analysis. Health & Social Care in the Community, 30(6), e5673-e5684. https://doi.org/10.1111/hsc.13995

Jirovec, R. L., & Hyduk, C. A. (1999). Type of volunteer experience and health among older adult volunteers. Journal of Gerontological Social Work, 30(3-4), 29-42. https://doi.org/10.1300/J083v30n03_04

Johnson, K. J. (2013). Volunteering among surviving spouses: The impact of volunteer activity on the health of the recently widowed. Graduate Doctoral Dissertations. 114. https://scholarworks.umb.edu/doctoral_dissertations/114

Jones, R., & Reynolds, F. (2019). The contribution of charity shop volunteering to a positive experience of ageing. Journal of Occupational Science, 26(4), 524-536. https://doi.org/10.1080/14427591.2019.1592697

Jongenelis, M. I., Jackson, B., Newton, R. U., & Pettigrew, S. (2022). Longitudinal associations between formal volunteering and well-being among retired older people: follow-up results from a randomized controlled trial. Aging & Mental Health, 26(2), 368-375. https://doi.org/10.1080/13607863.2021.1884845

Jongenelis, M. I., Jackson, B., Warburton, J., Newton, R. U., & Pettigrew, S. (2022). Aspects of formal volunteering that contribute to favourable psychological outcomes in older adults. European journal of ageing, 19(1), 107-116.

Jung, J. H., Ang, S., & Malhotra, R. (2023). Volunteering, religiosity, and quality of life in later life: Evidence from Singapore. Aging & Mental Health, 27(10), 2078-2087. https://doi.org/10.1007/s10433-021-00618-6

Kail, B. L., & Carr, D. C. (2020). More than selection effects: Volunteering is associated with benefits in cognitive functioning. The Journals of Gerontology: Series B, 75(8), 1741-1746. https://doi.org/10.1093/geronb/gbaa101

Kim, E. S., Whillans, A. V., Lee, M. T., Chen, Y., & VanderWeele, T. J. (2020). Volunteering and subsequent health and well-being in older adults: An outcome-wide longitudinal approach. American Journal of Preventive Medicine, 59(2), 176-186. https://doi.org/10.1016/j.amepre.2020.03.004

Kim, S., Halvorsen, C., Potter, C., & Faul, J. (2025). Does volunteering reduce epigenetic age acceleration among retired and working older adults? Results from the Health and Retirement Study. Social science & medicine, 364, 117501. https://doi.org/10.1016/j.socscimed.2024.117501

Kim, S., & Pan, X. (2025). The interplay between biological aging and volunteering engagement in predicting cognitive performance: Evidence from the Harmonized Cognitive Assessment Protocol study. The Journals of Gerontology, Series A: Biological Sciences and Medical Sciences, 80(7), glaf097. https://doi.org/10.1093/gerona/glaf097

Kim, S., Shiba, K., & Halvorsen, C. (2025). Reducing Hypertension Through Volunteering? Investigating the Potential Causal Link Between Volunteering Frequency and Blood Pressure by Gender, Race, and Age Groups. Biopsychosocial Science and Medicine, 87(5), 322-331. https://doi.org/10.1097/psy.0000000000001392

Kim, S., & Yoon, H. (2020). Volunteering, subjective sleep quality, and chronic inflammation: A 5-year follow-up of the National Social Life, Health, and Aging Project. Research on aging, 42(9-10), 291-299. https://doi.org/10.1177/0164027520922624

Klinedinst, N. J., & Resnick, B. (2014). Volunteering and depressive symptoms among residents in a continuing care retirement community. Journal of Gerontological Social Work, 57(1), 52-71. https://doi.org/10.1080/01634372.2013.867294

Konrath, S., Fuhrel-Forbis, A., Lou, A., & Brown, S. (2012). Motives for volunteering are associated with mortality risk in older adults. Health Psychology, 31(1), 87. https://doi.org/10.1037/a0025226

Krause, N. (2009). Church-based volunteering, providing informal support at church, and self-rated health in late life. Journal of aging and health, 21(1), 63-84. https://doi.org/10.1177/0898264308328638

Kritz, M., Ntoumanis, N., Mullan, B., Stathi, A., & Thøgersen-Ntoumani, C. (2021). Volunteer motivation and retention of older peer walk leaders: A 4-month long investigation. The Gerontologist, 61(7), 1118-1130. https://doi.org/10.1093/geront/gnaa159

Kuang, K., Huisingh-Scheetz, M., Miller, M. J., Waite, L., & Kotwal, A. A. (2023). The association of gait speed and self-reported difficulty walking with social isolation: a nationally-representative study. Journal of the American Geriatrics Society, 71(8), 2549-2556. https://doi.org/10.1111/jgs.18348

Labegalini, C. M. G., Uema, R. T. B., Carreira, L., Higarashi, I. H., & Baldissera, V. D. A. (2015). O trabalho voluntário na pastoral da criança na terceira idade: repercussões pessoais. Revista de Pesquisa Cuidado é Fundamental Online, 7(3), 2726-2737. https://doi.org/10.9789/2175-5361.2015.v7i3.2726-2737

Lakomý, M. (2023). The effect of roles prescribed by active ageing on quality of life across European regions. Ageing & Society, 43(3), 664-688. https://doi.org/10.1017/S0144686X21000726

Lam, A. H., Yeung, D. Y., & Chung, E. K. (2023). Benefits of volunteerism for middle-aged and older adults: Comparisons between types of volunteering activities. Ageing & Society, 43(10), 2287-2306. https://doi.org/10.1017/S0144686X21001665

Landry, C. (2017). A phenomenological investigation of the factors that influence motivation, recruitment and retention of volunteers age 65 and over. Pepperdine University ProQuest

Dissertations & Theses. https://www.proquest.com/docview/1948788082?pq-origsite=gscholar&fromopenview=true&sourcetype=Dissertations%20&%20Theses

Larkin, E., Sadler, S. E., & Mahler, J. (2005). Benefits of volunteering for older adults mentoring at-risk youth. Journal of Gerontological Social Work, 44(3-4), 23-37. https://doi.org/10.1300/J083v44n03_03

Lee, G. (2019). The relationship between volunteerism, personality, and psychological well-being among oldest old adults. Iowa State University ProQuest Dissertations & Theses, 27666800. https://www.proquest.com/openview/edab32664078baeb517f9555e7a2e65f/1?pq-origsite=gscholar&cbl=18750&diss=y

Lee, H. H., Kim, E. S., Kim, Y., Conroy, D. E., & VanderWeele, T. J. (2025). Exploring novel determinants of exercise behavior: a lagged exposure-wide approach. Annals of Behavioral Medicine, 59(1). https://doi.org/10.1093/abm/kaae082

Lee, K., Dabelko-Schoeny, H., & Richardson, V. E. (2021). Volunteering served as a transitional role that enhances the well-being and cognitive health among older adults with cognitive impairments. Journal of Applied Gerontology, 40(11), 1568-1578. https://doi.org/10.1177/0733464820982731

Lee, S. (2023). Loneliness, volunteering, and quality of life in European older adults. Activities, Adaptation & Aging, 47(2), 250-261. https://doi.org/10.1080/01924788.2022.2148408

Lee, S. (2024). The volunteer and charity work of European older adults: findings from SHARE. Journal of Nonprofit & Public Sector Marketing, 36(1), 22-36. https://doi.org/10.1080/10495142.2022.2130498

Lee, S. H., & Kim, Y. B. (2014). Which type of social activities decrease depression in the elderly? An analysis of a population-based study in South Korea. Iranian Journal of Public Health, 43(7), 903. http://ijph.tums.ac.ir/index.php/IJPH/article/view/6421/2439

Lee, S. J., Steinman, M. A., & Tan, E. J. (2011). Volunteering, driving status, and mortality in US retirees. Journal of the American Geriatrics Society, 59(2), 274-280. https://doi.org/10.1111/j.1532-5415.2010.03265.x

Lee, Y., Kim, Y.-M., Bronstein, L., & Fox, V. (2021). Older adult volunteers in intergenerational programs in educational settings across the globe. Educational Gerontology, 47(6), 247-256. https://doi.org/10.1080/03601277.2021.1915943

Li, Y.-P., Chen, Y.-M., & Chen, C.-H. (2013). Volunteer transitions and physical and psychological health among older adults in Taiwan. Journals of Gerontology Series B: Psychological Sciences and Social Sciences, 68(6), 997-1008. https://doi.org/10.1093/geronb/gbt098

Li, Y., & Ferraro, K. F. (2005). Volunteering and depression in later life: Social benefit or selection processes? Journal of health and social behavior, 46(1), 68-84. https://doi.org/10.1177/002214650504600106

Lim, E., Peng, C., & Burr, J. A. (2023). Friendship in Later Life: A Pathway Between Volunteering Hours and Depressive Symptoms. The Journals of Gerontology: Series B, 78(4), 673-683. https://doi.org/10.1093/geronb/gbac168

Lim, E., Uechi, M., Taira, D. A., Davis, J., Ishikawa, K. M., & Kaholokula, J. K. a. (2025). Exploring the Impact of Social Connection Dimensions on Cognitive Impairment in Older Adults: Observational Cohort Study. INQUIRY: The Journal of Health Care Organization, Provision, and Financing, 62. https://doi.org/10.1177/00469580251356121

Ling, W. H. H., Lee, W. P. V., Chui, W. H., & Sin, K. M. C. (2023). Older adults and volunteering: Mental wellness, motivation, and satisfaction. Activities, Adaptation & Aging, 47(4), 482-500. https://doi.org/10.1080/01924788.2023.2182489

Liu, Y., Duan, Y., & Xu, L. (2020). Volunteer service and positive attitudes toward aging among Chinese older adults: the mediating role of health. Social science & medicine, 265, 113535. https://doi.org/10.1016/j.socscimed.2020.113535

Lühr, M., Pavlova, M. K., & Luhmann, M. (2022). They are doing well, but is it by doing good? Pathways from nonpolitical and political volunteering to subjective well-being in age comparison. Journal of Happiness Studies, 23(5), 1969-1989. https://doi.org/10.1007/s10902-021-00480-4

Lum, T. Y., & Lightfoot, E. (2005). The effects of volunteering on the physical and mental health of older people. Research on aging, 27(1), 31-55. https://doi.org/10.1177/0164027504271349

Lyons, A., Alba, B., Waling, A., Minichiello, V., Hughes, M., Fredriksen-Goldsen, K. I., Edmonds, S., Blanchard, M., & Irlam, C. (2021). Volunteering among older lesbian and gay adults: Associations with mental, physical and social well-being. Journal of aging and health, 33(1-2), 3-13. https://doi.org/10.1177/0898264320952910

Matsuda, Y., Baba, A., Sugawara, I., Son, B. K., & Iijima, K. (2024). Multifaceted well-being experienced by community dwelling older adults engaged in volunteering activities of frailty prevention in Japan. Geriatrics & gerontology international, 24, 273-278. https://doi.org/10.1111/ggi.14826

Matthews, K., & Nazroo, J. (2021). The impact of volunteering and its characteristics on well-being after state pension age: Longitudinal evidence from the English Longitudinal Study of Ageing. The Journals of Gerontology: Series B, 76(3), 632-641. https://doi.org/10.1093/geronb/gbaa146

Mayers, T., Eto, S., Maki, N., Araki, A., & Matsuda, H. (2024). Volunteering and Its Association with Depression, Loneliness, and Lifestyle of Older Adults: Insights from a Japanese Cross-Sectional Study. Healthcare, 12(21), 2187. https://doi.org/10.3390/healthcare12212187

Mechakra-Tahiri, S.-D., Zunzunegui, M., Préville, M., & Dubé, M. (2010). Gender, social relationships and depressive disorders in adults aged 65 and over in Quebec. Health Promotion and Chronic Disease Prevention in Canada, 30(2). https://doi.org/10.24095/hpcdp.30.2.04

Melnyk, B. M., & Fineout-Overholt, E. (2022). Evidence-based practice in nursing & healthcare: A guide to best practice. Lippincott Williams & Wilkins.

Milbourn, B., Saraswati, J., & Buchanan, A. (2018). The relationship between time spent in volunteering activities and quality of life in adults over the age of 50 years: A systematic review. British Journal of Occupational Therapy, 81(11), 613-623. https://doi.org/10.1177/0308022618777219

Misener, K., Doherty, A., & Hamm-Kerwin, S. (2010). Learning from the experiences of older adult volunteers in sport: A serious leisure perspective. Journal of Leisure Research, 42(2), 267-289. https://doi.org/10.1080/00222216.2010.11950205

Moncayo-Hernández, B. A., Dueñas-Suarez, E. P., & Reyes-Ortiz, C. A. (2024). Relationship between social participation, children’s support, and social frailty with falls among older adults in Colombia. Annals of geriatric medicine and research, 28(3), 342. https://doi.org/10.4235/agmr.24.0059

Monserud, M. A. (2025). Social Leisure Activities and Cognitive Functioning among Married and Unmarried Older Men and Women in Mexico. Research on aging, 47(5-6), 282-296. https://doi.org/10.1177/01640275251315894

Moore, A., Motagh, S., Sadeghirad, B., Begum, H., Riva, J. J., Gaber, J., & Dolovich, L. (2021). Volunteer impact on health-related outcomes for seniors: A systematic review and meta-analysis. Canadian Geriatrics Journal, 24(1), 44. https://doi.org/10.5770/cgj.24.434

Morrow-Howell, N., Hong, S.-I., & Tang, F. (2009). Who benefits from volunteering? Variations in perceived benefits. The Gerontologist, 49(1), 91-102. https://doi.org/10.1093/geront/gnp007

Morrow-Howell, N., Kinnevy, S., & Mann, M. (1999). The perceived benefits of participating in volunteer and educational activities. Journal of Gerontological Social Work, 32(2), 65-80. https://doi.org/10.1300/J083v32n02_06

Mukherjee, D. (2010). An exploratory study of older adults' engagement with virtual volunteerism. Journal of Technology in Human Services, 28(3), 188-196. https://doi.org/10.1080/15228835.2010.508368

Musick, M. A., Herzog, A. R., & House, J. S. (1999). Volunteering and mortality among older adults: Findings from a national sample. The Journals of Gerontology Series B: Psychological Sciences and Social Sciences, 54(3), S173-S180. https://doi.org/10.1093/geronb/54B.3.S173

Musick, M. A., & Wilson, J. (2003). Volunteering and depression: The role of psychological and social resources in different age groups. Social science & medicine, 56(2), 259-269. https://doi.org/10.1016/S0277-9536(02)00025-4

Myers, D. R., Wolfer, T. A., & Sherr, M. (2013). Faith-outcomes for older adult volunteers in religious congregations. Social Work and Christianity, 40(4), 384. https://www.researchgate.net/profile/Adria-Navarro/publication/262567839_Evolving_pastoral_care_A_congregants'_transportation_ministry/links/00b4953bdaa775d77b000000/Evolving-pastoral-care-A-congregants-transportation-ministry.pdf#page=22

Nakamura, J. S., Kwok, C., Huang, A., Strecher, V. J., Kim, E. S., & Cole, S. W. (2023). Reduced epigenetic age in older adults who volunteer. Psychoneuroendocrinology, 148, 106000. https://doi.org/10.1016/j.psyneuen.2022.106000

Nakamura, J. S., Shiba, K., Shi, B., Leong, R. S., VanderWeele, T. J., & Kim, E. S. (2025). How is volunteering associated with reduced mortality? A mediator-wide approach. Health Psychology, 44(5), 518. https://doi.org/10.1037/hea0001429

Newman, S. (1983). The Experience of Senior Citizen Volunteers in Intergenerational Programs in Schools and the Relationship to Their Life Satisfaction. Final Report. https://eric.ed.gov/?id=ED263034

Newman, S., Karip, E., & Faux, R. B. (1995). Everyday memory function of older adults: the impact of intergenerational school volunteer programs. Educational Gerontology, 21(6), 569-580. https://doi.org/10.1080/0360127950210603

Newman, S., Vasudev, J., & Onawola, R. (1985). Older volunteers' perceptions of impacts of volunteering on their psychological well-being. Journal of Applied Gerontology, 4(2), 123-127. https://doi.org/10.1177/073346488500400215

Nichol, B., Wilson, R., Rodrigues, A., & Haighton, C. (2024). Exploring the effects of volunteering on the social, mental, and physical health and well-being of volunteers: an umbrella review. Voluntas: international journal of voluntary and nonprofit organizations, 35(1), 97-128. https://doi.org/10.1007/s11266-023-00573-z

Nonaka, K., Suzuki, H., Murayama, H., Hasebe, M., Koike, T., Kobayashi, E., & Fujiwara, Y. (2017). For how many days and what types of group activities should older Japanese adults be involved in to maintain health? A 4-year longitudinal study. PloS one, 12(9), e0183829. https://doi.org/10.1371/journal.pone.0183829

O'Reilly, D., Rosato, M., Ferry, F., Moriarty, J., & Leavy, G. (2017). Caregiving, volunteering or both? Comparing effects on health and mortality using census-based records from almost 250,000 people aged 65 and over. Age and ageing, 46(5), 821-826. https://doi.org/10.1093/ageing/afx017

O'Shea, E. (2006). An economic and social evaluation of the Senior Help Line in Ireland. Ageing & Society, 26(2), 267-284. https://doi.org/10.1017/S0144686X05004241

OCEBM Levels of Evidence Working Group Oxford. (2011). The Oxford 2011 Levels of Evidence. Oxford Centre for Evidence-Based Medicine. http://www.cebm.net/index.aspx?o=5653

Oman, D., Thoresen, C. E., & McMahon, K. (1999). Volunteerism and mortality among the community-dwelling elderly. Journal of Health Psychology, 4(3), 301-316. https://doi.org/10.1177/135910539900400301

Onyx, J., & Warburton, J. (2003). Volunteering and health among older people: A review. Australasian Journal on Ageing, 22(2), 65-69. https://doi.org/10.1111/j.1741-6612.2003.tb00468.x

Pardasani, M. (2018). Motivation to volunteer among senior center participants. Journal of Gerontological Social Work, 61(3), 313-333. https://doi.org/10.1080/01634372.2018.1433259

Parisi, J. M., Kuo, J., Rebok, G. W., Xue, Q.-L., Fried, L. P., Gruenewald, T. L., Huang, J., Seeman, T. E., Roth, D. L., & Tanner, E. K. (2015). Increases in lifestyle activities as a result of experience Corps® participation. Journal of Urban Health, 92, 55-66. https://doi.org/10.1007/s11524-014-9918-z

Parkinson, L., Warburton, J., Sibbritt, D., & Byles, J. (2010). Volunteering and older women: Psychosocial and health predictors of participation. Aging and Mental Health, 14(8), 917-927. https://doi.org/10.1080/13607861003801045

Pavlova, M. K., & Silbereisen, R. K. (2012). Participation in voluntary organizations and volunteer work as a compensation for the absence of work or partnership? Evidence from two German samples of younger and older adults. Journals of Gerontology Series B: Psychological Sciences and Social Sciences, 67(4), 514-524. https://doi.org/10.1093/geronb/gbs051

Pettigrew, S., Jongenelis, M. I., Jackson, B., Warburton, J., & Newton, R. U. (2020). A randomized controlled trial and pragmatic analysis of the effects of volunteering on the health and well-being of older people. Aging clinical and experimental research, 32, 711-721. https://doi.org/10.1007/s40520-019-01241-3

Proulx, C. M., Curl, A. L., & Ermer, A. E. (2018). Longitudinal associations between formal volunteering and cognitive functioning. The Journals of Gerontology: Series B, 73(3), 522-531. https://doi.org/10.1093/geronb/gbx110

Resnick, B., Klinedinst, J., Dorsey, S., Holtzman, L., & Abuelhiga, L. S. (2013). Volunteer behavior and factors that influence volunteering among residents in continuing care retirement communities. Journal of Housing for the Elderly, 27(1-2), 161-176. https://doi.org/10.1007/s40520-019-01241-3

Rogers, N. T., Demakakos, P., Taylor, M. S., Steptoe, A., Hamer, M., & Shankar, A. (2016). Volunteering is associated with increased survival in able-bodied participants of the English Longitudinal Study of Ageing. J Epidemiol Community Health, 70(6), 583-588. https://doi.org/10.1136/jech-2015-206305

Rook, K. S., & Sorkin, D. H. (2003). Fostering social ties through a volunteer role: Implications for older-adults' psychological health. The International Journal of Aging and Human Development, 57(4), 313-337. https://doi.org/10.2190/NBBN-EU3H-4Q1N-UXHR

Rowe, J. W., & Kahn, R. L. (1997). Successful aging. The Gerontologist, 37(4), 433-440. https://doi.org/10.1093/geront/37.4.433

Ryu, J., & Heo, J. (2018). Relationships between leisure activity types and well-being in older adults. Leisure Studies, 37(3), 331-342. https://doi.org/10.1080/02614367.2017.1370007

Sabin, E. P. (1993). Social relationships and mortality among the elderly. Journal of Applied Gerontology, 12(1), 44-60. https://doi.org/10.1177/073346489301200105

Sánchez-García, J., Lima, M. L., Marques, S., Gil-Lacruz, A. I., & Gil-Lacruz, M. (2025). National Perceptions of Over-70s' Status as a Moderator in the Link Between Volunteering and Subjective

Well-Being Among Older Adults in 29 European Countries. Journal of Applied Social Psychology, 55(6), 413-428. https://doi.org/10.1111/jasp.13099

Seeman, T., Merkin, S. S., Goldwater, D., & Cole, S. W. (2020). Intergenerational mentoring, eudaimonic well-being and gene regulation in older adults: A pilot study. Psychoneuroendocrinology, 111, 104468. https://doi.org/10.1016/j.psyneuen.2019.104468

Sellon, A. (2023). The importance of meaningful participation: health benefits of volunteerism for older adults with mobility-limiting disabilities. Ageing & Society, 43(4), 878-901. https://doi.org/10.1017/S0144686X21000842

Sellon, A. M. (2018). Volunteerism among older adults with mobility-limiting disabilities: an exploratory study, University of Kansas. https://hdl.handle.net/1808/27049

Serrat-Graboleda, E., González-Carrasco, M., Casas Aznar, F., Malo Cerrato, S., Cámara Liebana, D., & Roqueta-Vall-Llosera, M. (2021). Factors favoring and hindering volunteering by older adults and their relationship with subjective well-being: a mixed-method approach. International journal of environmental research and public health, 18(13), 6704. https://doi.org/10.3390/ijerph18136704

Sharifi, S., Khorzoughi, K. B., & Rahmati, M. (2024). The relationship between volunteering and cognitive performance in older adults: A systematic review. Geriatric Nursing, 55, 89-96. https://doi.org/10.1016/j.gerinurse.2023.10.020

Shimanuki, H., Honda, H., Ito, T., Kasai, T., Takato, J., Sakamoto, Y., Inuzuka, G., Ito, Y., Arayama, N., & Ueki, S. (2007). Relationships between volunteerism and social-physical health and QOL with community-dwelling elderly participating in a long-term care prevention programme. [Nihon Koshu Eisei Zasshi] Japanese Journal of Public Health, 54(11), 749-759. https://pubmed.ncbi.nlm.nih.gov/18186232/

Shmotkin, D., Blumstein, T., & Modan, B. (2003). Beyond keeping active: concomitants of being a volunteer in old-old age. Psychology and aging, 18(3), 602. https://doi.org/10.1037/0882-7974.18.3.602

Sneed, R. S., & Cohen, S. (2013). A prospective study of volunteerism and hypertension risk in older adults. Psychology and aging, 28(2), 578. https://doi.org/10.1037/a0032718

Stathi, A., Withall, J., Agyapong-Badu, S., Barrett, E., Kritz, M., Wills, D., Thogersen-Ntoumani, C., & Fox, K. R. (2021). Mobilising people as assets for active ageing promotion: a multi-stakeholder perspective on peer volunteering initiatives. BMC public health, 21, 1-12. https://doi.org/10.1186/s12889-020-10136-2

Stewart, K. E., Hand, C., Rudman, D. L., McGrath, C., McFarland, J., Gilliland, J., & Kinghorn, W. (2024). Invisible, unrecognised and undervalued: examining stories of unpaid work performed by older adults in their local neighbourhoods. Ageing & Society, 44(9), 2038-2064. https://doi.org/10.1017/S0144686X2200126X

Sun, P. C., Morrow-Howell, N., & Click, M. V. (2025). Variations in Benefits of Intergenerational Tutoring in the "New Normal". Journal of Applied Gerontology, 44(1), 126-135. https://doi.org/10.1093/geroni/igac059.2896

Sung, P., Chia, A., Chan, A., & Malhotra, R. (2023). Reciprocal relationship between lifelong learning and volunteering among older adults. The Journals of Gerontology: Series B, 78(5), 902-912. https://doi.org/10.1093/geronb/gbad003

Šupak Smolčić, V. (2013). Salami publication: Definitions and examples. Biochemia medica, 23(3), 237-241. https://doi.org/10.11613/BM.2013.030

Tamura, M., Hattori, S., Tsuji, T., Kondo, K., Hanazato, M., Tsuno, K., & Sakamaki, H. (2021). Community-level participation in volunteer groups and individual depressive symptoms in Japanese

older people: a three-year longitudinal multilevel analysis using JAGES data. International journal of environmental research and public health, 18(14), 7502. https://doi.org/10.3390/ijerph18147502

Tamura, M., Nakagomi, A., Ide, K., Kondo, K., Ojima, T., Takasugi, T., & Shiba, K. (2024). Volunteer group participation and subsequent health and well-being among older adults in Japan: An outcome-wide longitudinal approach. Archives of Gerontology and Geriatrics, 126, 105537. https://doi.org/10.1016/j.archger.2024.105537

Tan, E. J., Rebok, G. W., Yu, Q., Frangakis, C. E., Carlson, M. C., Wang, T., Ricks, M., Tanner, E. K., McGill, S., & Fried, L. P. (2009). The long-term relationship between high-intensity volunteering and physical activity in older African American women. Journals of Gerontology: Series B, 64(2), 304-311. https://doi.org/10.1093/geronb/gbn023

Tan, E. J., Xue, Q.-L., Li, T., Carlson, M. C., & Fried, L. P. (2006). Volunteering: a physical activity intervention for older adults—the experience Corps® program in Baltimore. Journal of Urban Health, 83(5), 954-969. https://doi.org/10.1007/s11524-006-9060-7

Tang, F. (2009). Late-life volunteering and trajectories of physical health. Journal of Applied Gerontology, 28(4), 524-533. https://doi.org/10.1177/0733464808327454

Taylor, D., Tucker, G., Wilson, D., Inacio, M., & Visvanathan, R. (2024). Understanding the important characteristics of neighbourhoods to facilitate ageing in place and longevity. Australasian Journal on Ageing, 43(3), 636-644. https://doi.org/10.1111/ajag.13318

Tiittanen, U., & Turjamaa, R. (2022). Social Inclusion and Communality of Volunteering: A Focus Group Study of Older People’s Experiences. International journal of environmental research and public health, 19(9), 5141. https://doi.org/10.3390/ijerph19095141

Tomioka, K., Kurumatani, N., & Hosoi, H. (2016). Association between social participation and instrumental activities of daily living among community-dwelling older adults. Journal of epidemiology, 26(10), 553-561. https://doi.org/10.2188/jea.JE20150253

Torres, Z., Martínez-Gregorio, S., & Oliver, A. (2023). Senior volunteers: Addressing loneliness in times of COVID-19. European journal of ageing, 20(1), 40. https://doi.org/10.1007/s10433-023-00788-5

United Nations Volunteers. (2018). The Scope and Scale of Global Volunteering: Current Estimates and Next Steps. Retrieved October from https://www.unv.org/sites/default/files/The%20Scope%20and%20Scale%20SWVR2018%20final.pdf

Varma, V. R., Carlson, M. C., Parisi, J. M., Tanner, E. K., McGill, S., Fried, L. P., Song, L. H., & Gruenewald, T. L. (2015). Experience Corps Baltimore: Exploring the stressors and rewards of high-intensity civic engagement. The Gerontologist, 55(6), 1038-1049. https://doi.org/10.1093/geront/gnu011

Varma, V. R., Tan, E. J., Gross, A. L., Harris, G., Romani, W., Fried, L. P., Rebok, G. W., & Carlson, M. C. (2016). Effect of community volunteering on physical activity: a randomized controlled trial. American Journal of Preventive Medicine, 50(1), 106-110. https://doi.org/10.1016/j.amepre.2015.06.015

Voloshina, I., Koroleva, K. Y., Elnikova, G., & Svishcheva, I. (2023). Volunteering as a resource for strengthening the mental health of the elderly. Problems of Social Hygiene, Public Health and History of Medicine, 31(S3), 696-700. https://doi.org/10.32687/0869-866X-2023-31-s1-696-700

Von Bonsdorff, M. B., & Rantanen, T. (2011). Benefits of formal voluntary work among older people. A review. Aging clinical and experimental research, 23, 162-169. https://doi.org/10.1007/BF03337746

Wang, Y., Li, Z., Gao, S., & Fu, C. (2021). Volunteer activity and depression among the elderly in China: A study on rural-urban differences. Medicine, 100(39), e27315. https://doi.org/10.1097/MD.0000000000027315

Warburton, J., & Peel, N. M. (2008). Volunteering as a productive ageing activity: the association with fall-related hip fracture in later life. European journal of ageing, 5, 129-136. https://doi.org/10.1007/s10433-008-0081-9

Warburton, J., Terry, D. J., Rosenman, L. S., & Shapiro, M. (2001). Differences between older volunteers and nonvolunteers: Attitudinal, normative, and control beliefs. Research on aging, 23(5), 586-605. https://doi.org/10.1177/0164027501235004

Warburton, J., & Winterton, R. (2017). A far greater sense of community: The impact of volunteer behaviour on the wellness of rural older Australians. Health & Place, 48, 132-138. https://doi.org/10.1016/j.healthplace.2017.10.005

Ward, M., Briggs, R., & Kenny, R. A. (2024). Social disconnection correlates of a "Wish to Die" among a large community-dwelling cohort of older adults. Frontiers in Public Health, 12, 1436218. https://doi.org/10.3389/fpubh.2024.1436218

Warner, L. M., Yeung, D. Y.-l., Jiang, D., Choi, N. G., Ho, R. T. H., Kwok, J. Y. Y., & Chou, K.-L. (2024). Effects of volunteering over six months on loneliness, social and mental health outcomes among older adults: The HEAL-HOA Dual Randomized Controlled Trial. The American journal of geriatric psychiatry, 32(5), 598-610. https://doi.org/10.1016/j.jagp.2023.12.022

Webster, N. J., Ajrouch, K. J., & Antonucci, T. C. (2021). Volunteering and health: The role of social network change. Social science & medicine, 285, 114274. https://doi.org/10.1016/j.socscimed.2021.114274

Weziak-Bialowolska, D., Skiba, R., & Bialowolski, P. (2024). Longitudinal reciprocal associations between volunteering, health and well-being: evidence for middle-aged and older adults in Europe. European Journal of Public Health, 34(3), 473-481. https://doi.org/10.1093/eurpub/ckae014

White, J., Falcioni, D., Thomacos, N., Mackenzie, L., Noble, N., & Boyes, A. (2025). Social Connection, Loneliness, and Solutions: Perceptions of Older Adults. Activities, Adaptation & Aging, 1-25. https://doi.org/10.1080/01924788.2025.2492991

Windsor, T. D., Anstey, K. J., & Rodgers, B. (2008). Volunteering and psychological well-being among young-old adults: How much is too much? The Gerontologist, 48(1), 59-70. https://doi.org/10.1093/geront/48.1.59

Withall, J., Thompson, J. L., Fox, K. R., Davis, M., Gray, S., De Koning, J., Lloyd, L., Parkhurst, G., & Stathi, A. (2018). Participant and public involvement in refining a peer-volunteering active aging intervention: Project ACE (Active, Connected, Engaged). The Gerontologist, 58(2), 362-375. https://doi.org/10.1093/geront/gnw148

World Health Organization. (2022). Ageing and health. https://www.who.int/news-room/fact-sheets/detail/ageing-and-health

World Health Organization. (2023). Progress report on the United Nations Decade of Healthy Ageing,

2021-2023. https://iris.who.int/bitstream/handle/10665/374192/9789240079694-eng.pdf?sequence=1

World Health Organization. (2024). Civic Participation and Employment. https://extranet.who.int/agefriendlyworld/age-friendly-practices/civic-participation-and-employment/

World Health Organization. (2025). Depressive disorder (depression). https://www.who.int/news-room/fact-sheets/detail/depression

Wu, A. M., Tang, C. S., & Yan, E. C. (2005). Post-retirement voluntary work and psychological functioning among older Chinese in Hong Kong. Journal of Cross-Cultural Gerontology, 20, 27-45. https://doi.org/10.1007/s10823-005-3796-5

Xi, Y., Mielenz, T. J., Andrews, H. F., Hill, L. L., Strogatz, D., DiGuiseppi, C., Betz, M. E., Jones, V., Eby, D. W., & Molnar, L. J. (2025). Prevalence of depression in older adults and the potential protective role of volunteering: Findings from the LongROAD study. Journal of the American Geriatrics Society, 73(4), 1041-1048. https://doi.org/10.1111/jgs.19349

Xie, L., & Han, W. (2024). The different roles of productive aging activities in the life satisfaction of older adults in urban and rural China. International Social Work, 67(1), 136-150. https://doi.org/10.1177/00208728221147612

Yamazaki, T., Sugawara, Y., Sone, T., & Tsuji, I. (2021). Subgroup characteristics of the association between volunteering and the risk of functional disability among older Japanese people: The Tsurugaya project. Archives of Gerontology and Geriatrics, 96, 104465. https://doi.org/10.1016/j.archger.2021.104465

Yang, J. (2020). Formal volunteering buffers the negative impact of unemployment among older workers: A longitudinal analysis. Journal of Gerontological Social Work, 63(3), 189-208. https://doi.org/10.1080/01634372.2020.1744057

Yasunaga, M., Murayama, Y., Takahashi, T., Ohba, H., Suzuki, H., Nonaka, K., Kuraoka, M., Sakurai, R., Nishi, M., & Sakuma, N. (2016). Multiple impacts of an intergenerational program in J apan: Evidence from the Research on Productivity through Intergenerational Sympathy Project. Geriatrics & gerontology international, 16, 98-109. https://doi.org/10.1111/ggi.12770

Yeung, D. Y.-L., Jiang, D., Warner, L. M., Choi, N. G., Ho, R. T. H., Kwok, J. Y. Y., & Chou, K.-L. (2025). The effects of volunteering on loneliness among lonely older adults: the HEAL-HOA dual randomised controlled trial. The Lancet Healthy Longevity, 6(1). https://doi.org/10.1016/j.lanhl.2024.100664

Yuen, H. K., Huang, P., Burik, J. K., & Smith, T. G. (2008). Impact of participating in volunteer activities for residents living in long-term-care facilities. The American Journal of Occupational Therapy, 62(1), 71-76. https://doi.org/10.5014/ajot.62.1.71
